# Nuclear lncRNA NORSF reduces E2 release in granulosa cells by sponging the endogenous small activating RNA miR-339

**DOI:** 10.1186/s12915-023-01731-x

**Published:** 2023-10-20

**Authors:** Miaomiao Wang, Yang Wang, Liu Yang, Xing Du, Qifa Li

**Affiliations:** College of Animal Science and Technology, Nanjing Agricultural University, Nanjing, 210095 China

**Keywords:** NORSF, ceRNA, miR-339, CYP19A1, saRNA, E2 release

## Abstract

**Background:**

Functioning as a competing endogenous RNA (ceRNA) is the main action mechanism of most cytoplasmic lncRNAs. However, it is not known whether this mechanism of action also exists in the nucleus.

**Results:**

We identified four nuclear lncRNAs that are presented in granulosa cells (GCs) and were differentially expressed during sow follicular atresia. Notably, similar to cytoplasmic lncRNAs, these nuclear lncRNAs also sponge miRNAs in the nucleus of GCs through direct interactions. Furthermore, NORSF (non-coding RNA involved in sow fertility), one of the nuclear lncRNA acts as a ceRNA of miR-339. Thereby, it relieves the regulatory effect of miR-339 on CYP19A1 encoding P450arom, a rate-limiting enzyme for E2 synthesis in GCs. Interestingly, miR-339 acts as a saRNA that activates CYP19A1 transcription and enhances E2 release by GCs through altering histone modifications in the promoter by directly binding to the CYP19A1 promoter. Functionally, NORSF inhibited E2 release by GCs via the miR-339 and CYP19A1 axis.

**Conclusions:**

Our findings highlight an unappreciated mechanism of nuclear lncRNAs and show it acts as a ceRNA, which may be a common lncRNA function in the cytoplasm and nucleus. We also identified a potential endogenous saRNA for improving female fertility and treating female infertility.

**Supplementary Information:**

The online version contains supplementary material available at 10.1186/s12915-023-01731-x.

## Background

In recent decades, non-coding RNAs (ncRNAs) have been the focus of biological research because of their abundance, wide distribution, and strong regulatory functions. Among ncRNAs, microRNAs (miRNAs) and long ncRNAs (lncRNAs) were discovered relatively early, and their mechanisms of action are ireasonably clear [1, 2]. miRNAs generally interact with the 3'-untranslated region (UTR) of target mRNAs, to post-transcriptionally repress mRNAs in the cytoplasm through mRNA decay and translation inhibition by RNA-RNA interactions [1]. Additionally, a few miRNAs in the cytoplasm regulate mRNA stability by interacting with the 5'-UTR of target mRNAs [3, 4]. Interestingly, a systematic analysis of miRNA subcellular distribution has shown that most miRNAs are present in both the nuclear and cytoplasmic compartments [5], with some miRNAs (e.g., miR-29b and miR-2337) being mainly expressed in the nucleus [6, 7]. However, few studies have focused on miRNAs in the nucleus. Unlike the cytoplasmic miRNAs, miRNAs in the nucleus usually activate target transcription through an RNA activation (RNAa) mechanism [7–10]. More recently, a report showed that miR-23a in the nucleus induces the transcription of NORHA, a follicular atresia-related lncRNA, by interacting with its promoter via an RNA–DNA interaction mechanism; thereby, it altered the granulosa cells (GCs) response to oxidative stress and controlled female fertility [11]. These results suggest that the mechanism of action of miRNAs is directly related to their subcellular localization (nucleus or cytoplasm).

Similar to miRNAs, subcellular localization is a critical determinant of the functions and mechanisms of lncRNAs [12]. However, unlike miRNAs, the mechanism of action of lncRNAs is more complex and diverse in both the nucleus and cytoplasm [13, 14]. In the nucleus, for instance, lncRNAs control the transcription of targets through recruiting chromatin remodeling factors and transcription factors by forming a DNA-RNA triplex with the enhancers and promoters of targets [15, 16], establishing nuclear structural domains by acting as scaffolds for nuclear complexes [17]. In the cytoplasm, lncRNAs affect the post-translational control of target mRNAs through sponging miRNAs and mRNAs by acting as competing endogenous RNAs (ceRNAs) [18], recruiting RNA-binding proteins (RBPs) to the 3'-UTRs of target mRNAs [19], or keeping RBPs away from recognition elements in target mRNAs [20]. In addition, lncRNAs retained in the cytoplasm also mediate protein–protein interactions and influence the nuclear translocation of target proteins [21, 22]. New mechanisms of action of lncRNAs in the nucleus and cytoplasm are being discovered. A recent study has suggested that phase separation is a widely used mechanism of action of lncRNAs [23].

A ceRNA mechanism between lncRNAs and miRNAs in the cytoplasm was the earliest discovered and most common mechanism of action of lncRNAs. In this study, we noticed an interesting phenomenon that may have been ignored, that is, both lncRNAs and miRNAs are distributed in the nucleus [7, 15]. Therefore, a ceRNA mechanism could exist between lncRNAs and miRNAs in the nucleus. Here, we validated this idea using lncRNAs distributed only in the nucleus (here, this type of lncRNA was defined as nuclear lncRNA).

## Results

### Identification of nuclear DELs during sow follicular atresia

A total of 228 lncRNAs in sow follicles were identified utilizing our previous RNA-seq data [24] with a higher criteria (reads ≥ 50 in healthy or early atretic follicles) (Additional file 1: Table S1). Next, prediction of the subcellular localization showed that 98 lncRNAs (42.98%) were discovered to be more abundant in the cytoplasm, 44 (19.30%) in the nucleus, and 86 (37.72%) in the other compartments such as the ribosome, cytosol, and exosome (Additional file 1: Table S2). Interestingly, the predicted subcellular localizations of three cytoplasmic lncRNAs, namely LOC102167901 (also known as NORHA), LOC102157709 (also known as BRE-AS), and LOC102162300 (also known as lnc2300), are consistent with our previous nuclear-cytoplasmic fractionation experiments in sow GCs [24–26].

**Fig. 1:**
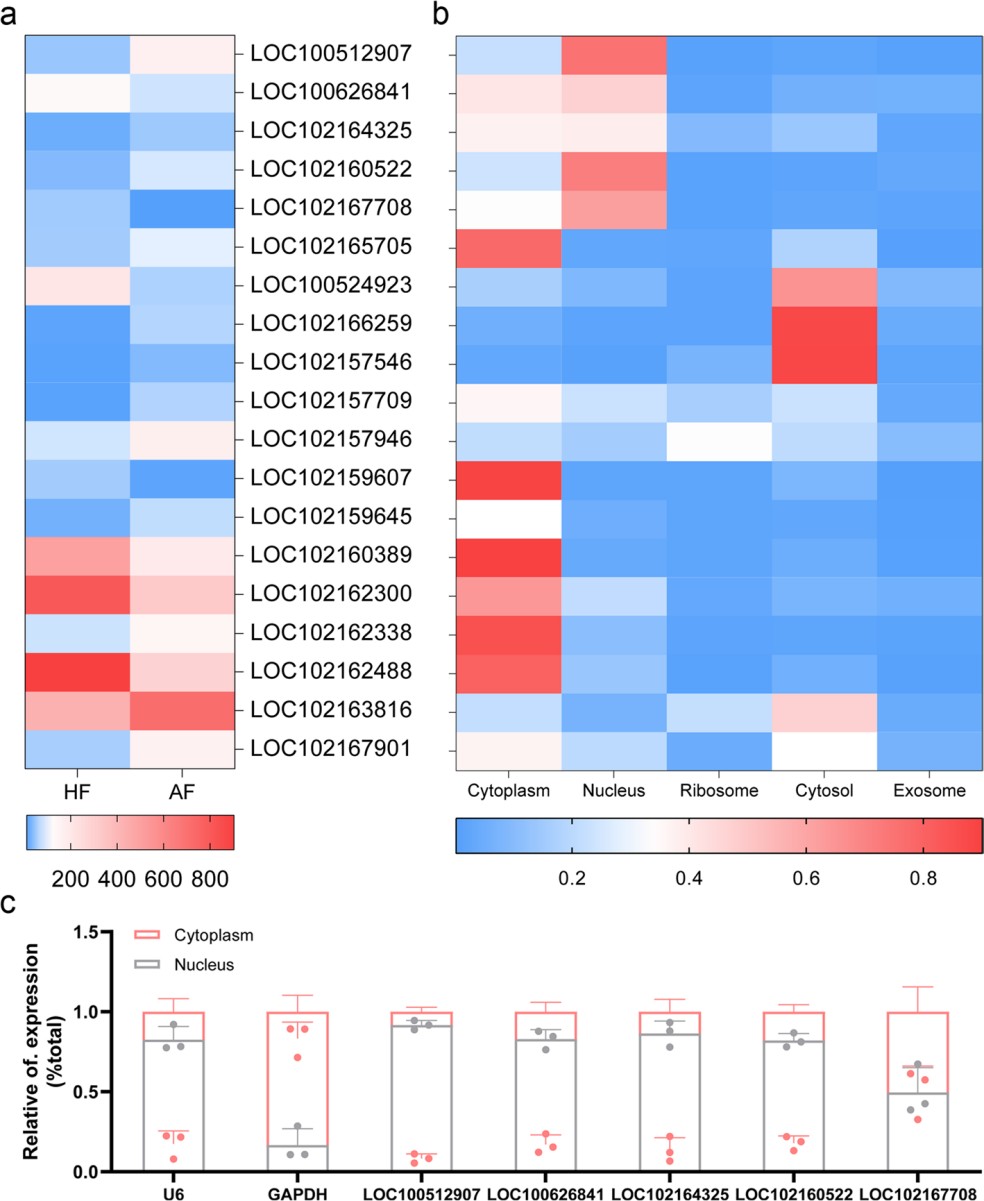
Identification of nuclear DELs during sow follicular atresia. **a** Heatmap of DELs in follicles during sow follicular atresia. RNA-seq data of sow follicles (3–5 mm diameter) were obtained from our previous study [24], and the screening of DELs add a new criterion, that is, reads ≥ 50 in healthy follicles or early atretic follicles. HF, healthy follicle. AF, early atretic follicle.** b** Prediction of subcellular localization of DELs. Expression scores of DELs in different compartments including cytoplasm, nucleus, ribosome, cytosol, and exosome were predicted using an online tool lncLocator (http://www.csbio.sjtu.edu.cn/bioinf/lncLocator/), and Heatmap was made by Graphpad prism v8.0 software. **c** Subcellular localization of DELs predicted to be more abundant in the nucleus in sow GCs. Levels of DELs in nuclear and cytoplasm fractions isolated from sow GCs were detected by qPCR. GAPDH and U6 were used for marker genes in the cytoplasm and nucleus, respectively. *n* = 3. Values are means ± SEM

Among the 228 follicular lncRNAs, 19 differentially expressed lncRNAs (DELs) during follicular atresia were identified (Fig. 1a). Of them, 47.37% (9 out of 19) of DELs were predicted to be more abundant in the cytoplasm, and 26.32% (5 out of 19) in the nucleus, 26.32% (5 out of 19) in the other compartments (Fig. 1b). In sow follicles, these five DELs predicted to be predominantly expressed in the nucleus were distributed in somatic cells, including GCs (Additional file 2: Fig. S1). Furthermore, nuclear-cytoplasmic fractionation assays of the five DELs predicted to be predominantly expressed in the nucleus and showed that four DELs, namely LOC100512907, LOC100626841, LOC102164325, and LOC102160522, are localized in the nucleus of sow GCs, and LOC102167708 was distributed in both the nucleus (50.47%) and the cytoplasm (49.52%) (Fig. 1c). These data delineated the nuclear-cytoplasmic distribution profile of lncRNAs in sow follicles, and identified four nuclear lncRNAs that were presented exclusively in the nucleus of sow GCs.

### Nuclear lncRNAs direct interact with miRNAs in the nucleus of GCs

**Fig. 2:**
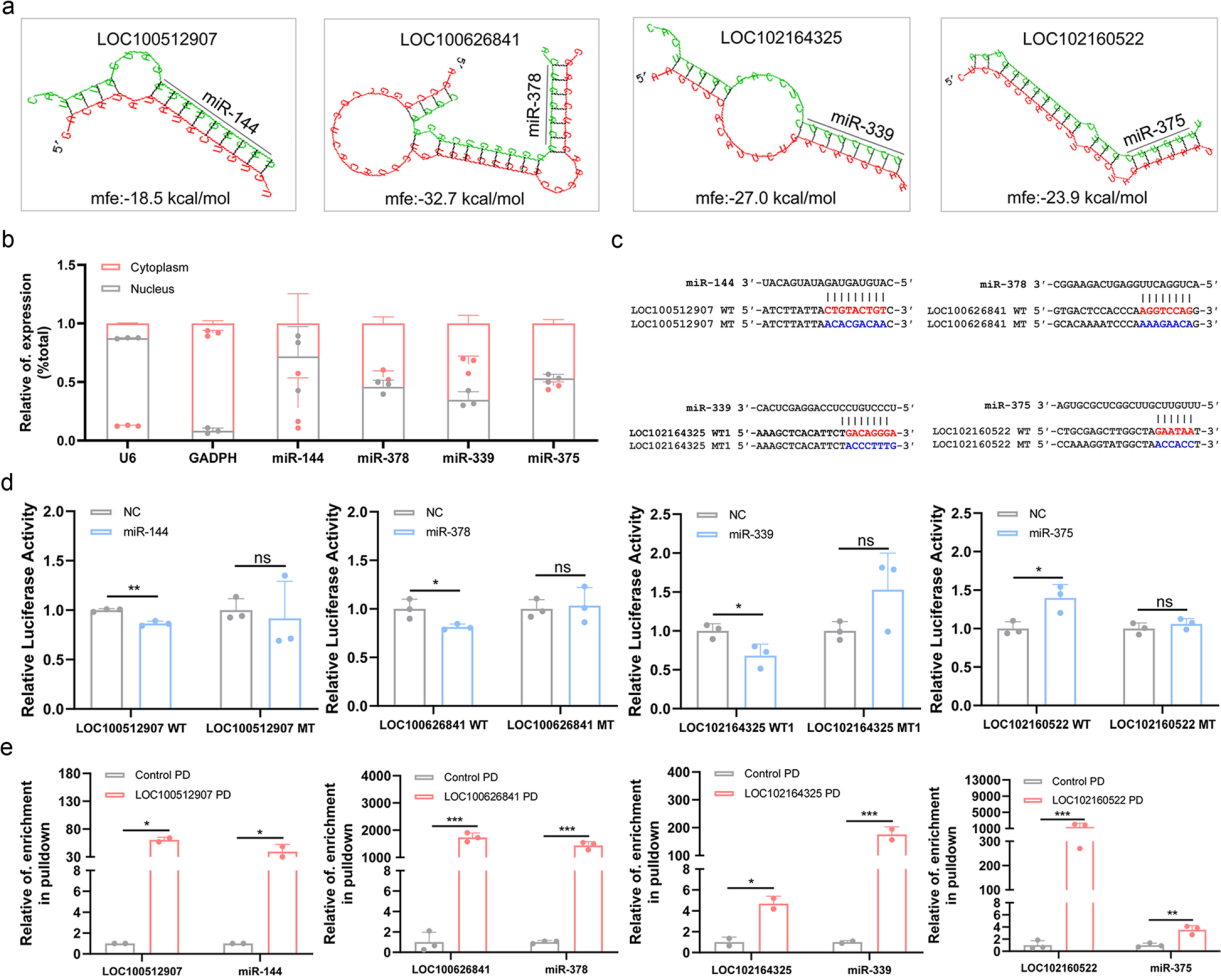
Nuclear lncRNAs direct interact with miRNAs in the nucleus of sow GCs. **a** miRNA response elements (MREs) of four miRNAs in the corresponding nuclear lncRNAs were predicted by RNAhybrid. For LOC102164325, three MRE motifs (MRE1, MRE2 and MRE3) of miR-339 were detected, and the minimum free energy (mfe) of MRE2 and MRE3 motifs binding to LOC102164325 are shown in Additional file 2: Fig. S3a, b. **b** Subcellular localization of miRNAs in sow GCs. Levels of miRNAs in nuclear and cytoplasm fractions isolated from sow GCs were detected by qPCR. GAPDH and U6 were used as marker genes in the cytoplasm and nucleus, respectively. *n* = 3. **c** Schematic showing the construction of reporter vector. LncRNAs containing MRE motif was amplified and cloned into a pGL3-basic vector. Diagram of reporter vectors of LOC102164325 with the other two MRE motifs are shown in Additional file 2: Fig. S3c. WT, wild-type. MT, mutant-type. WT1, wild-typed MRE1 of miR-339. MT1, mutant-typed MRE1 of miR-339.** d** Luciferase assay. GC line KGN were co-transfected with reporter vectors and the mimics for the corresponding miRNAs, and luciferase activity was detected. Luciferase activity of reporter vectors of LOC102164325 with MRE2 and MRE3 motifs are shown in Additional file 2: Fig. S3c. *n* = 3. **e** RNA pull-down assay. The physical interaction between lncRNAs and the corresponding miRNAs was detected. lncRNA-RNA complexes were pulled down from nuclear RNAs in sow GCs by using biotinylated probes for lncRNAs, levels of lncRNAs and miRNAs were determined by qPCR. PD, pull-down. Data for miR-378 experiments were analyzed using Moses Extreme Reactions in SPSS v26.0 software. *n* = 3 for LOC100626841 and LOC102160522, *n* = 2 for LOC100512907 and LOC102164325. Values are means ± SEM. *, *P* < 0.05; **, *P* < 0.01; ***, *P* < 0.001; ns, no significant

To investigate whether the four nuclear lncRNAs act as ceRNAs of miRNAs, putative miRNAs that interact with these nuclear lncRNAs were predicted using the online tool miRDB. Putative miRNA response elements (MREs) of 22, 4, 2, and 2 miRNAs were identified in LOC100512907, LOC100626841, LOC102164325, and LOC102160522 (Additional file 1: Table S3), respectively. Among them, miR-144, miR-378, miR-339, and miR-375 are related to sow ovarian functions [26–29] (Additional file 2: Fig. S2) and were predicted to have a strong binding ability with LOC100512907, LOC100626841, LOC102164325, and LOC102160522,using RNAhybrid (Fig. 2a; Additional file 2: Fig. S3a, b); therefore, they were selected as the candidate miRNAs for further analysis.

Importantly, subcellular localization assays revealed that these four miRNAs (miR-144, miR-378, miR-339, and miR-375) were distributed in the nucleus of sow GCs (Fig. 2b). To determine whether the four nuclear lncRNAs directly interacted with the four corresponding miRNAs in the nucleus, reporter constructs of lncRNAs were synthesized (Fig. 2c; Additional file 2: Fig. S3c). As expected, the luciferase activity of the reporter constructs of lncRNAs with wild-type MRE motifs, but not the mutated MRE motifs, were altered by the corresponding miRNAs (Fig. 2d; Additional file 2: Fig. S3d), indicating that the four nuclear lncRNAs interact with the corresponding miRNAs via the respective MRE motifs. Furthermore, RNA pull-down experiments performed using biotin-labeled lncRNA antisense-specific probes showed that the MRE motifs in nuclear lncRNAs directly interacted with the corresponding miRNAs in GCs (Fig. 2e). Taken together, these results suggest that nuclear lncRNAs directly interact with miRNAs in the nucleus of sow GCs. We speculated that nuclear lncRNAs may as adopt a ceRNA mechanism to sponge miRNAs in the nucleus, similar to lncRNAs and miRNAs co-located in the cytoplasm.

### Nuclear NORSF is an endogenous ceRNA of miR-339 in GCs

**Fig. 3:**
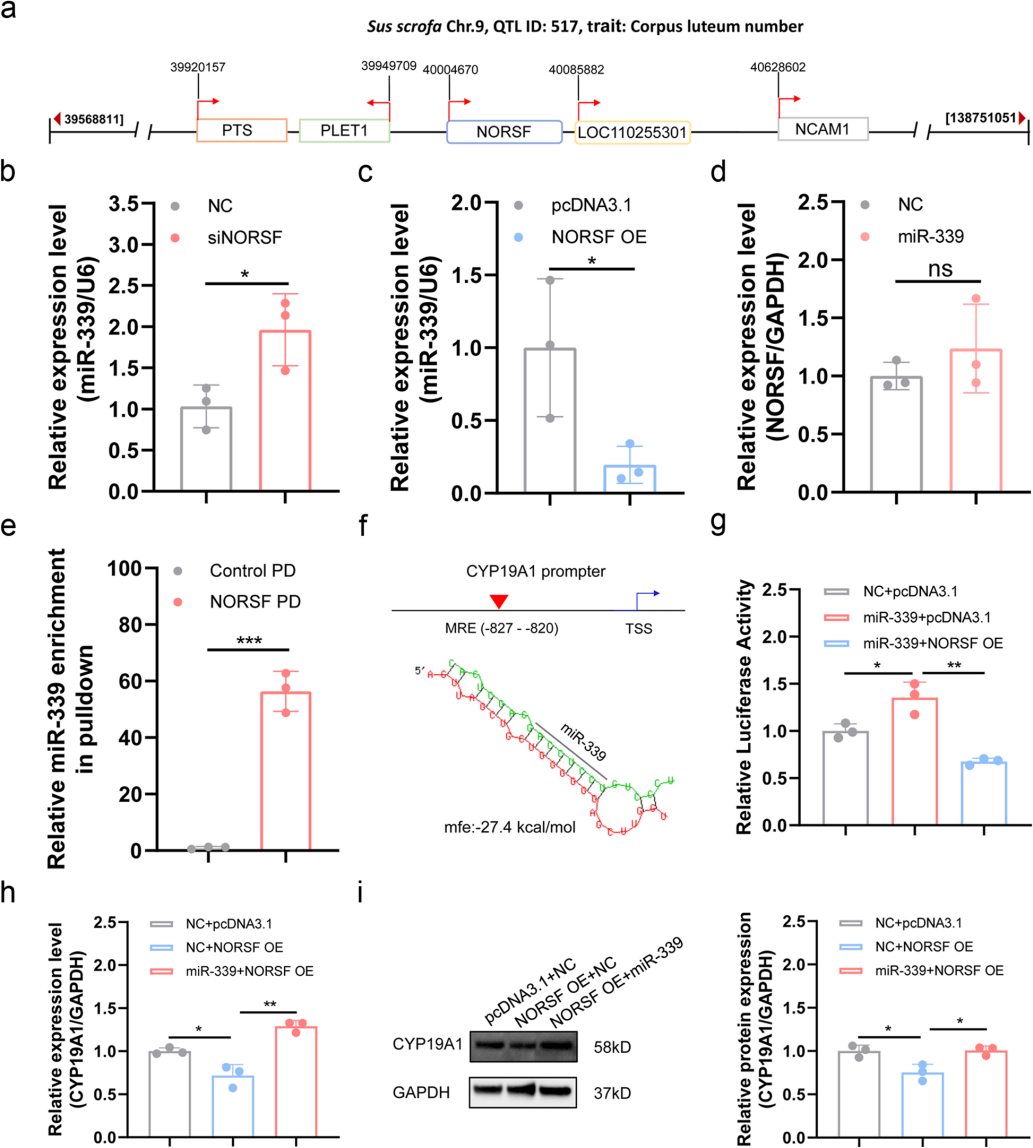
Nuclear NORSF is an endogenous ceRNA of miR-339 in sow GCs. **a** LOC102164325 is located in a QTL region for sow fertility trait. The information on this QTL is obtained from the pig QTLdb database (https://www.animalgenome.org/cgi-bin/QTLdb/SS/index). **b**-**c** miR-339 levels in sow GCs treated with a NORSF-siRNA (**b**) and an overexpression vector pcDNA3.1-NORSF (**c**), were determined by qPCR. Data were normalized to U6 levels. *n* = 3. **d** NORSF levels in GCs treated with miR-339 mimics were determined by qPCR. Data were normalized to GAPDH mRNA levels. *n* = 3. **e** In vivo RNA pull-down assay. GCs were cross-linked, NORSF-RNA complexes were pulled down by using biotinylated probes for NORSF, and miR-339 levels were determined by qPCR. The group without the addition of biotin-labeled probes is used as negative control. PD, pull-down. *n* = 3. **f** Schematic showing miR-339 MRE in the pig CYP19A1 PII promoter. The mfe is evaluated by RNAhybrid. **g** NORSF induces CYP19A1 promoter activity via miR-339. Luciferase assays were performed using a DLR™ assay system. *n* = 3. **h**-**i** NORSF induces CYP19A1 transcription in GCs via miR-339. After pcDNA3.1-NORSF and miR-339 mimics co-transferred into GCs for 48 h, CYP19A1 mRNA (**h**) and protein (**i**) levels were detected by qPCR and western blotting, and normalized by GAPDH mRNA and protein levels, respectively. *n* = 3. Values are means ± SEM. *, *P* < 0.05; **, *P* < 0.01; ***, *P* < 0.001; ns, no significant

Next, we further took LOC102164325 and miR-339 as an example to address the above hypothesis that nuclear lncRNAs could function as ceRNAs to sponge miRNAs in the nucleus of sow GCs, because they have the strongest relative binding activity among the four nuclear lncRNAs and their corresponding miRNAs (Additional file 1: Table S4). Rapid amplification of cDNA ends (RACE) assays showed that the LOC102164325 transcript was 748 bp in length, without canonical polyadenylation signal and polyadenylation tail at the 3′ end, and protein-coding potential (Additional file 2: Fig. S4a–e). Furthermore, LOC102164325 exhibits an evolutionary conservation with human lncRNA LOC124906953, both in sequence (with 75% nucleotide identity in partial sequences) and genome location (between two protein-coding genes PLET1 and NCAM1) (Additional file 2: Fig. S4f–g). Interestingly, LOC102164325 is located in a quantitative trait locus (QTL) for the sow fertility trait (QTL ID: 517) on pig chromosome 9 (Fig. 3a); therefore, we renamed it non-coding RNA involved in sow fertility or NORSF.

In sow GCs, miR-339 levels were significantly increased upon NORSF depletion, but significantly decreased upon NORSF overexpression (Fig. 3b, c), indicating that NORSF may sponge miR-339 in GCs via its MRE1 and MRE3 motifs, combined with luciferase assays (Fig. 2d; Additional file 2: Fig. S3d). In contrast, miR-339 had no inhibitory effect on NORSF expression in GCs (Fig. 3d). In addition, we verified the binding of NORSF and miR-339 by in vivo RNA pull-down assay, which showed that miR-339 was significantly enriched by NORSF in sow GCs in vivo (Fig. 3e). Together with RNA pull-down experiments in vitro (Fig. 2e), our findings suggest that nuclear NORSF acts as a ceRNA to sponge miR-339 in the nucleus of sow GCs in vitro and in vivo.

To confirm this conclusion, we investigated whether nuclear NORSF regulates the expression of the target of miR-339 in GCs. In the nucleus, miRNAs usually activate target transcription by interacting with promoters [30]. Interestingly, when we previously characterized the porcine CYP19A1 promoter, we detected an MRE motif of miR-339 in its PII promoter, which is an ovarian-specific promoter (Fig. 3f). Luciferase assay confirmed that NORSF overexpression reduced CYP19A1 transcriptional activity activated by miR-339 (Fig. 3g). Furthermore, miR-339 reversed the reduction of CYP19A1 levels caused by NORSF overexpression (Fig. 3h, i; Additional file 2: Fig. S5a–d). Collectively, these results support the observation that nuclear NORSF acts as a ceRNA to competently bind miR-339 to the CYP19A1 promoter in GCs.

### miR-339 acts as a saRNA to activate CYP19A1 transcription in GCs

**Fig. 4:**
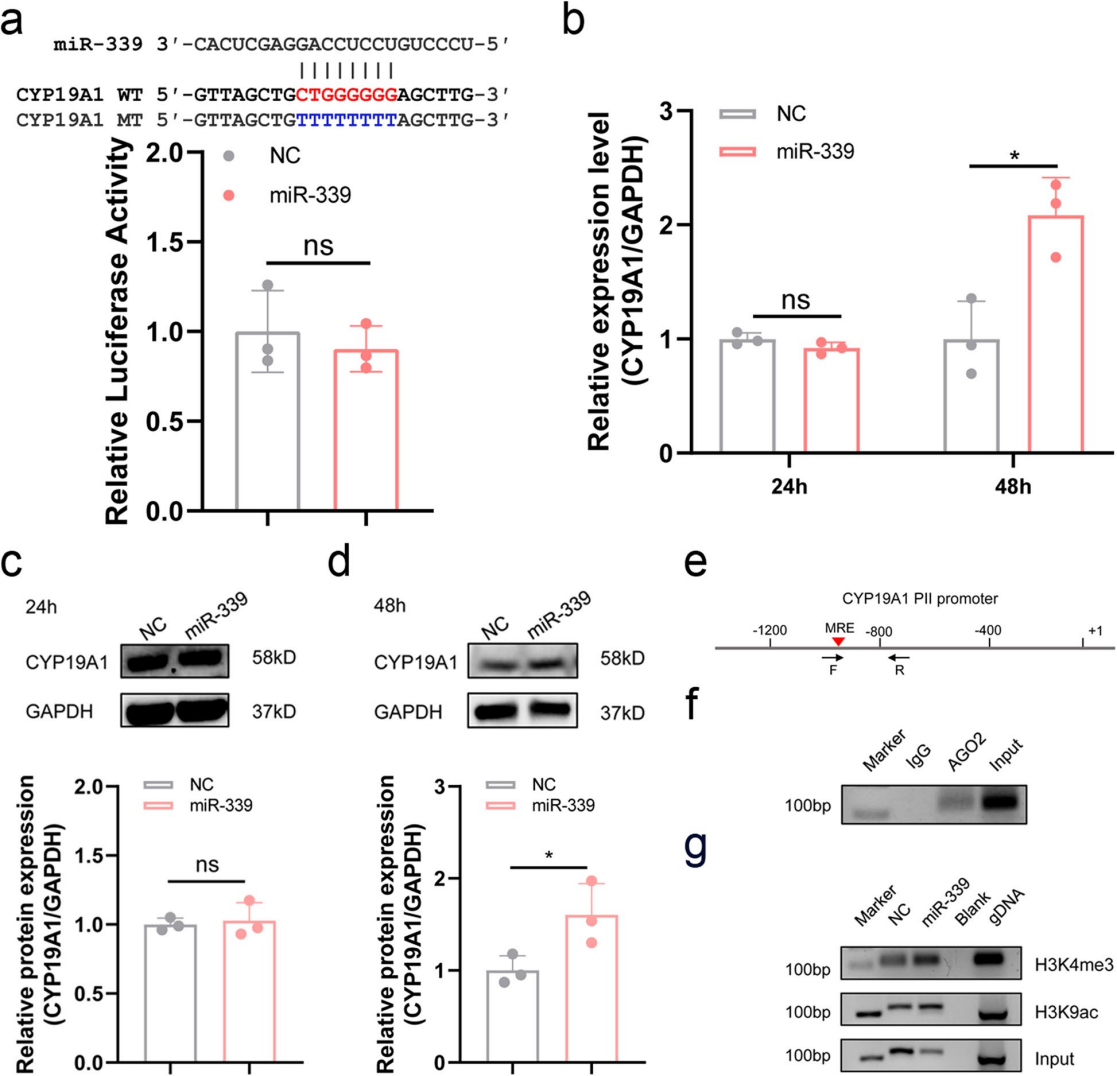
miR-339 activates CYP19A1 transcription in sow GCs by acting as an endogenous saRNA. **a** miR-339 does not influence the activity of CYP19A1 promoter with the miR-339 MRE was mutated. A reporter vector of the mutant CYP19A1 promoter containing the MRE motif of miR-339 was generated, and co-treated with miR-339 mimics, and luciferase activity was measured. *n* = 3. **b** miR-339 activates CYP19A1 transcription in GCs. qPCR was performed to detect the CYP19A1 mRNA levels in GCs after treated with miR-339 mimics for 24 h, and 48 h. GAPDH was used as an internal reference. *n* = 3. **c**-**d** miR-339 increases CYP19A1 protein levels in GCs. Western blotting was performed to detect the CYP19A1 protein levels in GCs after treated with miR-339 mimics for 24 h (**c**), and 48 h (**d**). GAPDH was used as an internal reference. *n* = 3. **e**–**g** ChIP assays. Specific primers for amplifying CYP19A1 promoter containing miR-339 MRE motif are designed (**e**) to amplify DNA products enriched by specific antibodies against AGO2 (**f**), and H3K4me and H3K9ac (**g**) in GCs. F, forward primer. R, reverse primer. Values are means ± SEM. *, *P* < 0.05; ns, no significant

Our results demonstrated that miR-339 induces CYP19A1 transcription in sow GCs, which is consistent with one of the five main characteristics of saRNAs, that is, the activation of target transcription. We also observed that miR-339 did not activate the CYP19A1 PII promoter with a mutated MRE motif (Fig. 4a). These results suggest that miR-339 activates CYP19A1 transcription in sow GCs via its MRE motif within the PII promoter, which is in accordance with the second characteristic of saRNAs, that is, binding to target promoters.

Next, we investigated whether miR-339 conforms to other saRNA characteristics. In sow GCs cultured in vitro, miR-339 activated CYP19A1 transcription 48 h after treatment with miR-339 mimics, rather than 24 h for the RNAi mechanism of miRNAs (Fig. 4b), which is in accordance with the third characteristic of saRNA, that is, delayed effect. Similarly, CYP19A1 protein levels were significantly altered 48 h after treatment, but not at 24 h, in GCs (Fig. 4c, d). Notably, we detected the enrichment of AGO2, a core component of the RNA-induced transcriptional activation (RITA) complex [31], at the miR-339 MRE motif of the CYP19A1 PII promoter in sow GCs (Fig. 4e, f), which is in accordance with the fourth characteristic of saRNAs, that is, dependence on the RITA complex. Finally, Chromatin immunoprecipitation (ChIP) assays using specific antibodies showed that the enrichment of histone modifiers, including H3K4me3 and H3K9ac, increased at the miR-339 MRE motif of the CYP19A1 PII promoter in sow GCs treated with miR-339 mimics (Fig. 4g). This was in accordance with the fifth characteristic of saRNAs, that is, altering histone modifications. In summary, our data revealed that miR-339 conforms to the basic characteristics of an endogenous saRNA that activates CYP19A1 transcription in sow GCs.

### NORSF suppresses E2 release through impairing miR-339/CYP19A1 axis

**Fig. 5:**
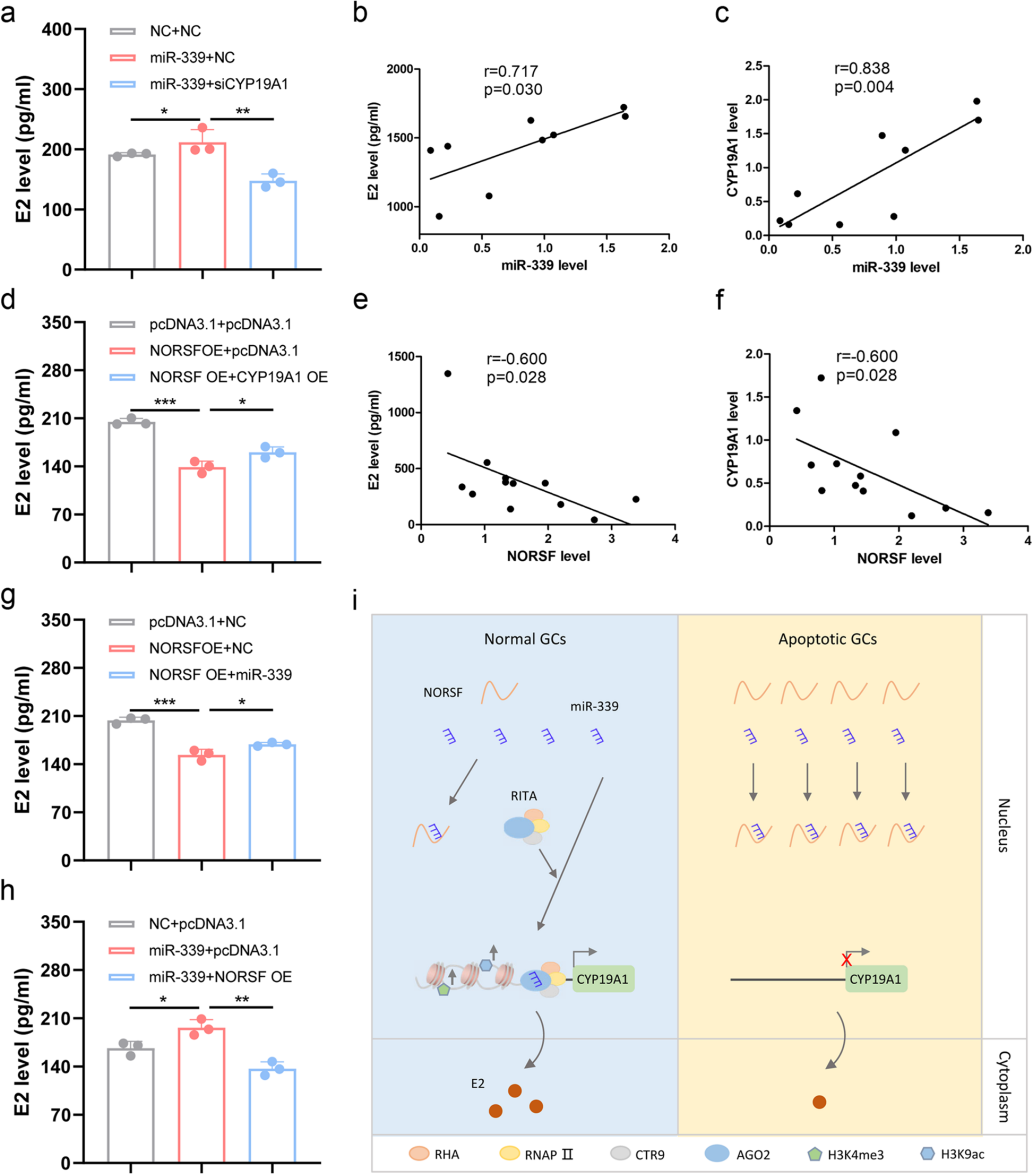
NORSF reduces E2 release in sow GCs through the miR-339/CYP19A1 axis. **a** miR-339 induces E2 release in sow GCs via CYP19A1. GCs were co-treated with miR-339 mimics and pcDNA3.1-CYP19A1, E2 concentration in the culture medium was measured by ELISA. *n* = 3. **b-c** Correlation analysis between miR-339 levels in sow follicles and E2 concentration in follicular fluid (**b**), or CYP19A1 mRNA levels in follicles (**c**). *n* = 9. **d** NORSF reduces E2 release in CGs via CYP19A1. *n* = 3. **e**–**f** Correlation analysis between NORSF levels in sow follicles and E2 concentration in follicular fluid (**e**), or CYP19A1 mRNA levels in follicles (**f**). *n* = 12. **g** miR-339 mediates NORSF reduction of E2 release by GCs. GCs were co-treated with pcDNA3.1-NORSF and miR-339 mimics, E2 concentration in culture medium was measured by ELISA. *n* = 3. **h** NORSF suppresses E2 release induced by miR-339 in GCs. GCs were co-treated with miR-339 mimics and pcDNA3.1-NORSF, E2 concentration in culture medium was measured by ELISA. *n* = 3. **i** Working model of lncRNA NORSF. As an ceRNA of miR-339, NORSF with low abundance cannot sponge miR-339, the latter binds to CYP19A1 promoter to activate its transcription and induces E2 secretion by GCs through acting as an endogenous saRNA. Values are means ± SEM. *, *P* < 0.05; **, *P* < 0.01; ***, *P* < 0.001

P450arom, encoded by CYP19A1 is necessary for catalyzing the final step of E2 biosynthesis. Therefore, we investigated the role of miR-339 in E2 release from sow GCs. ELISA assay showed that miR-339 induced E2 release by GCs (Fig. 5a). Furthermore, CYP19A1 depletion suppressed the E2 release induced by miR-339 (Fig. 5a), indicating that miR-339 induces E2 release by GCs via CYP19A1. Additionally, a significant positive correlation between sow follicular miR-339 levels and E2 concentration in the follicular fluid and follicular CYP19A1 levels was observed (Fig. 5b, c), suggesting that miR-339 is strongly involved in E2 release in sow GCs in vitro and in vivo.

Next, we investigated whether NORSF was also involved in E2 release by sow GCs. In sow GCs cultured in vitro, E2 concentration in the culture medium was significantly decreased by NORSF overexpression (Fig. 5d). Furthermore, CYP19A1 overexpression suppressed NORSF-induced E2 reduction (Fig. 5d), suggesting that NORSF reduces E2 release by GCs through suppressing CYP19A1 expression. Additionally, a significant negative correlation between follicular NORSF levels and E2 concentration in the follicular fluid, and follicular CYP19A1 levels was observed (Fig. 5e, f), suggesting that NORSF is also strongly involved in E2 release in sow GCs in vitro and in vivo.

Finally, we tested whether miR-339 mediated NORSF reduction of E2 release in sow GCs. Co-transfection experiments showed that miR-339 ameliorated the reduction in E2 release caused by NORSF (Fig. 5g), indicating that miR-339 mediates NORSF reduction of E2 release in GCs. Additionally, NORSF suppressed E2 release induced by miR-339 (Fig. 5h). Together, these data suggest that NORSF suppresses E2 release through impairing the miR-339/CYP19A1 axis, which further confirmed that NORSF is an endogenous ceRNA of miR-339 in controlling E2 release by sow GCs.

## Discussion

LncRNAs are well-known class of epigenetic factors that control gene expression at multiple levels (e.g. transcriptional, post-transcriptional, and post-translational levels) [32–35]. In the nucleus, lncRNAs mainly regulate gene transcription by recruiting various modulators (e.g. transcription factors, epigenetic regulators, and histone-modified complexes) to the regulatory regions such as enhancer, promoter, and introns near the 5'-end [36–38]. lncMREF, for instance, an important regulator of muscle regeneration, recruits SMARCA5 (a chromatin remodeler and an SWI/SNF protein) to the promoters of myogenic regulators (e.g. MyoD), thereby facilitating the genomic binding of the histone-modified complexes p300/CBP/H3K27ac to control the transcription of these myogenic regulators [36]. A more recent study showed that an antisense lncRNA TGFB2-AS1 suppresses TGFB2 transcription in breast cancer cells through sequestering SMARCA4 (a SWI/SNF complex catalytic subunit) away from the TGFB2 promoter [39]. Here, we showed a novel mechanism of action of nucleus-localized lncRNAs, by which they function as ceRNAs to sponge miRNAs in the nucleus of sow GCs. Furthermore, we used the nuclear lncRNA NORSF to demonstrate that it prevented miR-339 from interacting with the CYP19A1 PII promoter, thereby affecting CYP19A1 transcription in sow GCs. It would be reasonable to speculate that this ceRNA mode of nuclear lncRNAs is not unique to sow GCs, but crucial for nuclear lncRNAs in other cell types of other organisms including humans, to play a regulatory role. We counted 28217 lncRNAs confirmed by nucleoplasmic localization assays in 15 human cell types and showed that the proportion of lncRNAs that were more abundant in the nucleus was high in all cell types, ranging from 57.90% (H1.hESC) to 77.69% (SK.MEL.5 cells) (Additional file 2: Fig. S6). Furthermore, 34 putative miRNAs in the nucleus were predicted to interact with these nuclear lncRNAs in these cell types (Additional file 2: Fig. S7 and Additional file 1: Table S5). Next, we selected two well-known nuclear lncRNAs, MALAT1 [40] and NEAT1 [41], to validate the ceRNA mechanism of nuclear lncRNAs using RNA pull-down. Both MALAT1 and NEAT1 were confirmed to be nuclear lncRNAs and sponged multiple nucleus-expressed miRNAs, including miR-24, miR-26, miR-29b and miR-373 in the human GC line KGN (Additional file 2: Fig. S8). Altogether, our findings uncover an underappreciated action model of nuclear lncRNAs, which not only enriches the mechanism of action of lncRNAs, but also establishes a new form of interaction between lncRNAs and miRNAs in the nucleus.

P450arom encoded by CYP19A1, catalyzes the final step of the E2 synthesis pathway. E2 secreted by target cells controls various biological processes of health and disease in organisms through classical genomic signaling, mediated by nuclear receptors including estrogen receptor α and β, and non-genomic signaling mediated by membrane receptors (i.e., G protein-coupled receptor 30, GPR30) [42–44]. Due to the importance of E2 in female reproduction, the regulation of its synthesis has always been a topic of interest, and P450arom and CYP19A1 have become important targets for regulating various normal physiological processes and treating diseases [45–47]. Several miRNAs targeting the CYP19A1 3'-UTR have been shown to inhibit its expression in normal and pathological cells [45, 48]. In this study, we revealed a new mode by which miRNAs activate CYP19A1 transcription and induce E2 secretion in sow GCs. Nucleus-expressed miR-339 acts as a saRNA to directly bind to the CYP19A1 promoter. saRNAs are a class of double-stranded ncRNAs with a length similar to that of miRNAs, and they have proven to be potential RNA-based drugs for RNA-based therapeutics. They include some coding mRNAs and other ncRNAs such as siRNAs, RNA aptamers, and RNA guides [49–51]. In 2008, miR-373 was the first miRNA that was demonstrated to act as an endogenous saRNA and directly interact with the promoters of target genes (including E-cadherin and CSDC2) to activate their transcription through an RNA activation (RNAa) mechanism [9]. Subsequently, multiple endogenous RNAs have been identified in multiple species, including miR-155 in human umbilical vein endothelial cells [52], let-7i in mouse CD4 + T-cells [53], lin-4 in *Caenorhabditis elegans* hypodermal seam cells [54], and miR-23a in sow GCs [11]. In summary, we identified, for the first time, an endogenous saRNA that induces E2 synthesis in GCs via the activation of CYP19A1 transcription. Our next step will be to evaluate whether miR-339 can serve as a small molecule non-hormone RNA drug for regulating sow reproduction, potentially replacing hormone drugs (such as GnRH) used for controlling follicular development and ovulation in sow production, and reducing the side effects of hormone drugs. Additionally, we also showed that the miR-339 sequence in mammals (including humans, mice, and pigs) is relatively conserved, and an MRE motif was identified in the human CYP19A1 promoter (Additional file 2: Fig. S9), indicating that miR-339 is a potential saRNA that targets CYP19A1 gene and controls E2 synthesis in humans. Furthermore, multiple MRE motifs of miR-339 were discovered in human LOC124906953 (Additional file 2: Fig. S10). This may have some significance in developing a potential small-molecule non-hormonal regulator to regulate female reproduction and improve female fertility in farm animals or treat estrogen-related diseases in humans. With the development of in vivo delivery systems, some saRNAs such as MTL-CEBPA [55] have been used in clinical practice to treat human diseases. In the future, saRNA therapy may be applicable to a wide range of diseases [56]. In conclusion, we determined the subcellular localization profiles of lncRNAs in sow follicles and DELs during follicular atresia, and identified four nuclear lncRNAs in sow GCs. Notably, we identified a novel mechanism of action of nuclear lncRNAs, by which they act as ceRNAs to sponge miRNAs in the nucleus. Furthermore, we defined a new signaling pathway underlying the nuclear lncRNA NORSF that controls E2 release in GCs; NORSF sponges endogenous miR-339 in the nucleus of GCs, preventing it from binding to and activating the CYP19A1 promoter, thereby inhibiting CYP19A1 transcription and E2 synthesis (Fig. 5i).

## Conclusions

Our findings uncover an underappreciated mode of action of nuclear lncRNAs. i.e., nuclear lncRNAs can act as ceRNAs to sponge miRNAs in the nucleus. Furthermore, we identified a novel signaling pathway of nuclear lncRNA NORSF/miR-339/CYP19A1 that controls E2 release in GCs. This study enriches our understanding of the action mechanisms of lncRNAs and provides a potential endogenous saRNA for controlling female reproduction.

## Methods

### Sows

For GC collection and culture, ovary samples of 210 Duroc-Landrace-Yorkshire sows with sexually mature and healthy were collected from the Lukou abattoir (Nanjing, China).

### Bioinformatic analysis

Online prediction of subcellular localization was performed using lncLocator (http://www.csbio.sjtu.edu.cn/bioinf/lncLocator/). The minimum free energy (MFE) of miRNA binding was evaluated using RNAhybrid (http://bibiserv.techfak.uni-bielefeld.de/rnahybrid/). MRE motifs were detected using miRDB (http://mirdb.org/). The subcellular localization data for lncRNAs and miRNAs in human cells were downloaded from lncATLAS (https://lncatlas.crg.eu/) and RNALOCATE (http://www.rna-society.org/rna-locate/), respectively. LncBook 2.0 (https://ngdc.cncb.ac.cn/lncbook/) was used to predict the miRNAs that interact with lncRNAs. QTL-related data were obtained from the Pig QTL Database (https://www.animalgenome.org/cgi-bin/QTLdb/SS/index).

### Collection of primary GCs and theca cells

Follicles with diameters ranging 3–5 mm were isolated from fresh ovaries using a sterilized scalpel. The follicular cavity was extracted with a syringe and centrifuged to collect GCs. For collecting theca cells, the follicular membrane was repeatedly scraped with forceps to remove mural GCs, then digested with 0.3 mg/mL collagenase I (#BS163, Biosharp, Guangzhou, China) at 37 °C for 1.5 h, filtered with a 100 μm cell sieve, and purified by Percoll gradients (#BS012, Biosharp).

### Nuclear-cytoplasmic fractionation assays

Sow GCs were resuspended using a 4 °C lysis buffer (1 mM RNase inhibitor, 10 mM Tris, 1 mM NP-40, and 0.1 mM EDTA), and lysed at 4 °C for 10 min followed by centrifugation at 10,000 × g for 4 min. The supernatant was the cytoplasmic extract and the precipitate was the nuclear lysate. The precipitate was resuspended in 4 °C DEPC water (containing 1 mM RNase inhibitor) for 3 min, and centrifuged at 10,000 × g and 4 °C for 4 min; subsequently, the supernatant was removed to obtain the bottom nuclear extract. The TRIzol reagent (#RG-51001A; Angle Gene, Beijing, China) was used to isolate total RNA from the cytoplasm and nucleus. GAPDH and U6 were used as cytoplasmic and nuclear markers, respectively. Primers are listed in Additional file 1: Table S6.

### RNA preparation and qPCR

TRIzol reagent (#RG-51001A, Angle Gene) was used to extract and purify total RNA. HiScript II Q Select RT SuperMix (#R232-01, Vazyme, Nanjing, China) and HiScript III 1st Strand cDNA Synthesis Kit (#R312-01, Vazyme) were used to reverse transcribe total RNA to cDNAs to quantify non-miRNAs genes and miRNAs, respectively. qPCR was executed using an AceQ qPCR SYBR Green Master Mix (#Q111-02, Vazyme), and the reaction parameters were as follows: hold stage 95 °C for 5 min; PCR stage 40 cycles at 95 °C for 10 s, 60 °C annealing for 30 s, and 95 °C for 15 s; melt curve stage 60 °C for 1 min and 95 °C for 1 s. 2^−ΔΔCT^ method was used to estimate the relative transcript levels, and GAPDH (for non-miRNA genes) and U6 (for miRNAs) were the internal controls. Primers are listed in Additional file 1: Table S6.

### Plasmid construction

Reporter vectors for lncRNAs or the CYP19A1 promoter containing the wild-type or mutated MRE motifs of the corresponding miRNAs were synthesized by Tsingke (Shanghai, China). For overexpression vector, cDNA fragment containing the full-length NORSF transcript was isolated from sow GCs, and inserted into the pcDNA3.1 vector (#V790-20, Invitrogen, USA). Primers are listed in Additional file 1: Table S6.

### Cell culture and transfection

GCs were inoculated into cell culture plates filling with DMEM/F12 medium (containing 15% FBS and 1% PS) (#11320033, Gibco, CA, USA) and then transferred into in an incubator at 37 °C with 5% CO_2_. The human GC line KGN was cultured in the RPMI 1640 medium (#22400105, Gibco) containing 10% FBS and 1% PS. Once the cell density exceeded 80%, plasmids or oligonucleotides were transfected with the Lipofectamine 3000 (#L3000015, Invitrogen). All the oligonucleotides (Additional file 1: Table S7) were generated by GenePharma (Shanghai, China).

### Luciferase assay

After 24 h of transfection, cells were collected, and detection of luciferase activity was performed using a DLR™ assay system (#E1910, Promega). The ratio of *firefly* and *Renilla* luciferase activities is taken as the relative luciferase activity.

### RNA pull-down in vitro

20 µg of total RNA from the nucleus of GCs or KGN cells was incubated overnight with 5 µg of biotin-labeled probes at 4 °C. Streptavidin magnetic beads (#LSKMAGTO2, Merck Millipore, GER) were added and incubated for 2 h at 25 °C to pull down the lncRNA-RNA complexes. LncRNAs and miRNAs in the pull-down products were quantified by qPCR. Biotin-labeled probes for lncRNAs were synthesized by Sangon (Nanjing, China) and their sequences are shown in Additional file 1: Table S8.

### RNA pull-down in vivo

Sow GCs were digested with trypsin (#25200056, Gibco) and cross-linked by shaking with 1% glutaraldehyde (#R20509, Shanghai Yuanye, China) at 25 °C for 10 min. After washing with PBS and subsequent lysis, GCs were exposed to biotin-labeled probes for NORSF and incubated at room temperature for 4 h. To pull down the NORSF-RNA complexes, streptavidin magnetic beads (#LSKMAGTO2) were added to the culture medium and incubated at 25 °C for 2 h. miR-339 in the pull-down products was quantified by qPCR.

### RACE assays

To obtain the full-length sequence of the NORSF transcript, RACE assays were performed using the SMARTer® RACE 5′/3′ kit (#634858, Clontech Laboratories, Mountain View, USA). Briefly, 1 μg of high-integrity total RNA from sow GCs was used as a template to synthesize first-strand cDNA. Gene-specific primers were prepared as follows: 5′-CCG CCT CGG CTT CCT ACT AAA TCA CC-3′ (the reversed primer for 5′-RACE assay) and 5′-AGG GGG TGA TTT AGT AGG AAG CCG AGG-3′ (the forward primer for 3′-RACE assay). The 5′-RACE nested reverse primer is 5′-TCG CTT CCC TGT CAG AAT GTG-3′. The 3′-RACE nested forward primer is 5′-CAA CGC AGA GAA GAC CGA AAG-3′. PCR products were isolated using with a 1.5% agarose gel, and purified using a DNA Gel Extraction kit (#GE0101-200; TsingKe); subsequently, they were inserted into pClone007 vector (#TSV-007S; TsingKe) for sequencing.

### Protein isolation and western blotting

RIPA lysis buffer (#AC23181, Bioworld, Nanjing, China) containing 0.1% PMSF (v/v) was used for isolating total protein from GCs. Proteins were denatured and placed on 12% SDS-PAGE gel loading wells and separated by electrophoresis, following which they were transferred to PVDF membranes, and blocked by 5% skim milk at room temperature for 1.5 h. Then, primary antibodies were used for incubation at 4 °C overnight, followed by incubation with secondary antibody at room temperature for 1 h. The primary antibodies used were anti-CYP19A1 (#A2161, ABclonal, 1:1000,), and anti-GAPDH (#TA802519, ORIGENE, 1:3000), and the secondary antibody was horseradish peroxidase-conjugated mouse anti-rabbit IgG (#D110065, Sangon Biotech, 1:1000). Raw images of the high-resolution blots were obtained using ImageQuant LAS-4000 (GE Healthcare, Chicago, IL, USA), and grayscale values were analyzed using ImageJ software.

### ChIP assays

ChIP assays were performed as previously described [57]. Antibodies, including anti-H3K4me3 (#9751S, anti-H3K9ac (#9649S), and IgG antibody (#2985S) were purchased from Cell Signaling Technology (Danvers, MA, USA), and anti-AGO2 (#D121540) was purchased from Sangon. Enrichment of target DNA fragments was determined by qPCR and untreated chromatin was used as the NC and input control. Primers are listed in Additional file 1: Table S6.

### E2 concentration determination

Follicular fluid was extracted using a syringe, and the supernatant was collected post centrifugation at 10,000 × g for 3 min. After treatment for 48 h, the GC culture medium was centrifuged at 10,000 × g for 3 min to collect the supernatant. The E2 concentration in the supernatant was measured by ELISA using a Detection Kit for Estrodial (E2) (#E9096-E2, North Biotech, Beijing, China), according to the manufacturer's instructions.

### Statistics

Experiments were performed in three biological replicates unless otherwise indicated, and statistically analyzed using SPSS v26.0 (IBM-SPSS, IL, USA) and GraphPad v8.0 (San Diego, CA, USA) software. Significance was assessed using the Student's t-test and one-way analysis of variance with the Tukey's Honest Significant Difference. Before conducting multiple comparisons, the normality of the data and the homogeneity of variance have been confirmed using SPSS v26.0 software (IBM-SPSS). The correlation coefficient was calculated using Pearson’s test.

### Supplementary Information

**Additional file 1:** **Table S1.** LncRNAs in sow follicles. **Table S2.** Pedication of subcellular localization of sow follicular lncRNAs. **Table S3.** miRNAs potentially interacting with nuclear lncRNAs in sow GCs. **Table S4.** The relative binding activity between nuclear lncRNAs and their corresponding miRNAs. **Table S5.** miRNAs in the nucleus potentially interacting with nuclear lncRNAs. **Table S6.** Primers designed for reverse-transcription, qPCR, ChIP and plasmid construction. **Table S7.** Oligonucleotide sequences used in this study. **Table S8.** Biotin-labeled antisense probes.

**Additional file 2:** **Fig. S1.** The RNA expression profile of five DELs in sow follicular theca cells and GCs. **Fig. S2.** Heatmap of miR-144, miR-378, miR-339 and miR-375 signals in sow follicles and GCs. **Fig. S3.** Other two potential MREs of miR-339 in LOC102164325. **Fig. S4.** Identification of lncRNA NORSF. **Fig. S5.** NORSF negatively regulates CYP19A1 expression in GCs. **Fig. S6.** Percentage of lncRNA nucleoplasm in multiple human cell types. **Fig. S7.** Interaction networks of lncRNAs and miRNAs in the nucleus of multiple human cell types. **Fig. S8.** Nuclear lncRNAs MALAT1 and NEAT1 interact with multiple miRNAs in the nucleus of human GCs. **Fig. S9.** miR-339 mature sequences are highly conserved among vertebrates. **Fig. S10.** The MRE motifs of miR-339 in human LOC124906953.

**Additional file 3:** **Table S1.** Subcellular localization data. **Table S2.** Luciferase activity assay data. **Table S3.** RNA-pulldown in vitro data. **Table S4.** Quantitative real-time PCR raw data. **Table S5.** RNA-pulldown in vivo data. **Table S6.** E2 assay raw data. **Table S7.** Correlation analysis data.

**Additional file 4:** **Original image 1.** Electrophoresis original images. **Original image 2.** RACE original image. **Original image 3.** Western blotting original images. **Original image 4.** CHIP original images.

## Data Availability

All data generated or analysed during this study are included in this published article and its supplementary information files. Additional information in support of the results of this study is also available from the corresponding author upon request.

## Abbreviations

ceRNA: competing endogenous RNA
saRNAs: small activating RNAs
NORSF: non-coding RNA involved in sow fertility
ncRNAs: non-coding RNAs
miRNAs: microRNAs
lncRNAs: long non-coding RNAs
UTR: untranslated region
RNAa: RNA activation
RBPs: RNA-binding proteins
DELs: differentially expressed lncRNAs
MREs: miRNA response elements
RACE: Rapid amplification of cDNA ends
QTL: quantitative trait locus
RITA: RNA-induced transcriptional activation
ChIP: Chromatin immunoprecipitation
MFE: minimum free energy
