# Supplementary material for: Nuclear lncRNA NORSF reduces E2 release in granulosa cells by sponging the endogenous small activating RNA miR-339
Source: BMC Biol. 2023 Oct 20;21:221. doi: 10.1186/s12915-023-01731-x (PMC10588145; doi:10.1186/s12915-023-01731-x)
Supplement: Supplementary file 1 — Additional file 1: Table S1. LncRNAs in sow follicles. Table S2. Pedication of subcellular localization of sow follicular lncRNAs. Table S3. miRNAs potentially interacting with nuclear lncRNAs in sow GCs. Table S4. The relative binding activity between nuclear lncRNAs and their corresponding miRNAs. Table S5. miRNAs in the nucleus potentially interacting with nuclear lncRNAs. Table S6. Primers designed for reverse-transcription, qPCR, ChIP and plasmid construction. Table S7. Oligonucleotide sequences used in this study. Table S8. Biotin-labeled antisense probes. [file 12915_2023_1731_MOESM1_ESM.docx]

**Supplementary tables**

**Table S1 LncRNAs in sow follicles**

| LncRNAs | HF1 | HF2 | HF3 | AF1 | AF2 | AF3 | Coding probability^a^ |
| --- | --- | --- | --- | --- | --- | --- | --- |
| LOC100152407 | 1615 | 1055 | 1711 | 1231 | 1343 | 1021 | 0.0715 |
| LOC100510918 | 64 | 27 | 43 | 41 | 44 | 50 | 0.2417 |
| LOC100510923 | 144 | 86 | 125 | 58 | 87 | 56 | 0.0751 |
| LOC100512560 | 603 | 331 | 655 | 239 | 353 | 373 | 0.0112 |
| LOC100512700 | 1596 | 1098 | 2014 | 1413 | 2140 | 1796 | 0.0822 |
| LOC100512907 | 41 | 64 | 33 | 248 | 116 | 116 | 0.4650 |
| LOC100513133 | 9192 | 7792 | 8164 | 15875 | 8039 | 7763 | 0.0761 |
| LOC100513188 | 122 | 146 | 155 | 270 | 128 | 232 | 0.1131 |
| LOC100513397 | 1247 | 1419 | 1788 | 902 | 1676 | 1182 | 0.1912 |
| LOC100514147 | 238 | 221 | 294 | 335 | 288 | 326 | 0.0539 |
| LOC100514807 | 170 | 127 | 213 | 74 | 178 | 95 | 0.0410 |
| LOC100514834 | 184 | 347 | 636 | 256 | 272 | 68 | 0.2400 |
| LOC100514924 | 914 | 695 | 1058 | 529 | 977 | 594 | 0.0647 |
| LOC100515112 | 49 | 28 | 51 | 89 | 68 | 50 | 0.2327 |
| LOC100515492 | 23 | 59 | 58 | 22 | 58 | 58 | 0.1246 |
| LOC100515792 | 430 | 358 | 467 | 284 | 403 | 235 | 0.0648 |
| LOC100516105 | 345 | 277 | 380 | 191 | 454 | 193 | 0.0176 |
| LOC100516145 | 218 | 302 | 349 | 233 | 306 | 354 | 0.0295 |
| LOC100516649 | 51 | 117 | 75 | 177 | 61 | 104 | 0.0212 |
| LOC100516669 | 36 | 67 | 23 | 58 | 63 | 54 | 0.0071 |
| LOC100516731 | 517 | 545 | 761 | 730 | 786 | 703 | 0.1250 |
| LOC100517116 | 996 | 686 | 1615 | 478 | 1833 | 528 | 0.2339 |
| LOC100517727 | 103 | 69 | 86 | 37 | 55 | 44 | 0.1185 |
| LOC100518120 | 54 | 59 | 30 | 56 | 48 | 41 | 0.0109 |
| LOC100519283 | 1919 | 1206 | 2721 | 599 | 1378 | 814 | 0.0426 |
| LOC100520275 | 204 | 188 | 96 | 94 | 103 | 114 | 0.0252 |
| LOC100520518 | 32 | 39 | 56 | 62 | 34 | 63 | 0.2094 |
| LOC100521322 | 100 | 113 | 114 | 111 | 92 | 123 | 0.1784 |
| LOC100521518 | 2916 | 1815 | 4277 | 1604 | 3898 | 2008 | 0.2229 |
| LOC100521937 | 45 | 41 | 87 | 28 | 59 | 27 | 0.0976 |
| LOC100522143 | 655 | 649 | 738 | 443 | 753 | 515 | 0.0414 |
| LOC100522281 | 86 | 80 | 70 | 31 | 71 | 31 | 0.2569 |
| LOC100522818 | 273 | 228 | 321 | 326 | 236 | 268 | 0.0834 |
| LOC100522935 | 70 | 45 | 124 | 55 | 103 | 35 | 0.0514 |
| LOC100523870 | 40 | 28 | 52 | 40 | 54 | 26 | 0.1041 |
| LOC100524915 | 366 | 209 | 498 | 145 | 377 | 180 | 0.3652 |
| LOC100524923 | 252 | 191 | 162 | 78 | 24 | 69 | 0.1156 |
| LOC100525078 | 122 | 125 | 111 | 94 | 120 | 97 | 0.4763 |
| LOC100525571 | 172 | 145 | 195 | 92 | 148 | 77 | 0.1371 |
| LOC100525590 | 4705 | 3242 | 5897 | 4172 | 5153 | 4362 | 0.0211 |
| LOC100525935 | 1372 | 1074 | 1875 | 933 | 1845 | 1204 | 0.0510 |
| LOC100526084 | 575 | 633 | 790 | 628 | 715 | 745 | 0.0462 |
| LOC100621235 | 114 | 83 | 83 | 59 | 78 | 78 | 0.0065 |
| LOC100621455 | 16737 | 14571 | 21294 | 16776 | 27836 | 12892 | 0.0758 |
| LOC100622037 | 701 | 330 | 750 | 284 | 493 | 316 | 0.1837 |
| LOC100622481 | 1136 | 849 | 1579 | 1150 | 1509 | 1165 | 0.0195 |
| LOC100623096 | 1527 | 2119 | 2052 | 1794 | 2497 | 2568 | 0.2866 |
| LOC100624113 | 7885 | 6065 | 11988 | 8236 | 9064 | 9416 | 0.0544 |
| LOC100624406 | 50 | 51 | 38 | 33 | 31 | 34 | 0.0827 |
| LOC100625214 | 216 | 155 | 314 | 114 | 261 | 140 | 0.2958 |
| LOC100626258 | 137 | 146 | 182 | 120 | 233 | 160 | 0.0106 |
| LOC100626841 | 1488 | 1117 | 1658 | 421 | 952 | 510 | 0.0462 |
| LOC100627868 | 961 | 822 | 1191 | 918 | 1196 | 1371 | 0.0244 |
| LOC100627892 | 799 | 391 | 1354 | 669 | 1499 | 827 | 0.0189 |
| LOC100627949 | 1900 | 1175 | 2381 | 652 | 2563 | 890 | 1.0000 |
| LOC100628140 | 308 | 182 | 319 | 165 | 189 | 183 | 0.1887 |
| LOC100736638 | 670 | 628 | 806 | 391 | 755 | 408 | 0.0347 |
| LOC100736972 | 183 | 133 | 227 | 80 | 178 | 115 | 0.2975 |
| LOC100737186 | 90 | 84 | 64 | 88 | 77 | 70 | 0.0758 |
| LOC100737511 | 323 | 211 | 368 | 139 | 349 | 227 | 0.0506 |
| LOC100737961 | 33 | 50 | 31 | 25 | 57 | 39 | 0.1102 |
| LOC100738812 | 84 | 87 | 50 | 95 | 107 | 90 | 0.3824 |
| LOC100739121 | 386 | 283 | 451 | 139 | 338 | 127 | 0.2190 |
| LOC100739565 | 1560 | 809 | 1698 | 341 | 1295 | 537 | 0.0243 |
| LOC100739688 | 79 | 57 | 44 | 35 | 28 | 22 | 0.0619 |
| LOC100739791 | 1377 | 860 | 1994 | 363 | 1654 | 690 | 0.0681 |
| LOC102157424 | 108 | 148 | 84 | 68 | 76 | 47 | 0.1040 |
| LOC102157454 | 52 | 79 | 61 | 42 | 56 | 37 | 0.0409 |
| LOC102157484 | 118 | 111 | 66 | 63 | 124 | 102 | 0.1860 |
| LOC102157546 | 12 | 7 | 18 | 56 | 11 | 36 | 0.1578 |
| LOC102157581 | 133 | 145 | 130 | 141 | 186 | 150 | 0.1368 |
| LOC102157630 | 2434 | 2587 | 2027 | 703 | 1750 | 860 | 0.0897 |
| LOC102157665 | 66 | 62 | 34 | 33 | 59 | 23 | 0.1840 |
| LOC102157672 | 523 | 397 | 682 | 580 | 677 | 468 | 0.0365 |
| LOC102157709 | 10 | 11 | 16 | 86 | 23 | 66 | 0.0329 |
| LOC102157766 | 65 | 30 | 72 | 33 | 69 | 26 | 0.0060 |
| LOC102157854 | 312 | 215 | 219 | 185 | 254 | 212 | 0.0599 |
| LOC102157897 | 533 | 417 | 676 | 337 | 589 | 442 | 0.0462 |
| LOC102157946 | 71 | 109 | 45 | 235 | 58 | 206 | 0.0383 |
| LOC102158087 | 149 | 187 | 173 | 139 | 112 | 127 | 0.0431 |
| LOC102158315 | 87 | 65 | 67 | 63 | 51 | 52 | 0.0317 |
| LOC102158334 | 51 | 40 | 46 | 31 | 56 | 23 | 0.1131 |
| LOC102158335 | 606 | 35 | 536 | 24 | 402 | 175 | 0.1624 |
| LOC102158363 | 149 | 103 | 136 | 73 | 148 | 69 | 0.0517 |
| LOC102158467 | 146 | 65 | 160 | 130 | 136 | 71 | 0.0183 |
| LOC102158524 | 65 | 103 | 127 | 142 | 116 | 130 | 0.3466 |
| LOC102158553 | 77 | 102 | 59 | 53 | 62 | 43 | 0.0609 |
| LOC102158687 | 75 | 62 | 85 | 44 | 62 | 58 | 0.0261 |
| LOC102158750 | 48 | 28 | 60 | 35 | 17 | 23 | 0.1087 |
| LOC102158821 | 76 | 39 | 75 | 48 | 60 | 46 | 0.0538 |
| LOC102158857 | 69 | 85 | 85 | 50 | 89 | 72 | 0.1252 |
| LOC102158889 | 51 | 31 | 82 | 15 | 108 | 33 | 0.0504 |
| LOC102159050 | 41 | 37 | 51 | 20 | 42 | 41 | 0.0135 |
| LOC102159054 | 85 | 101 | 90 | 71 | 61 | 80 | 0.0604 |
| LOC102159128 | 49 | 67 | 56 | 59 | 51 | 56 | 0.0262 |
| LOC102159203 | 32 | 38 | 37 | 41 | 56 | 46 | 0.0809 |
| LOC102159434 | 157 | 112 | 162 | 136 | 199 | 120 | 0.1867 |
| LOC102159502 | 52 | 42 | 76 | 46 | 56 | 39 | 0.1989 |
| LOC102159588 | 237 | 261 | 232 | 192 | 166 | 98 | 0.0581 |
| LOC102159607 | 50 | 43 | 61 | 13 | 15 | 15 | 0.2309 |
| LOC102159627 | 76 | 112 | 91 | 69 | 77 | 77 | 0.0524 |
| LOC102159645 | 23 | 25 | 44 | 81 | 34 | 100 | 0.2599 |
| LOC102159707 | 95 | 117 | 81 | 186 | 59 | 66 | 0.0548 |
| LOC102159960 | 45 | 79 | 65 | 63 | 83 | 33 | 0.1887 |
| LOC102159985 | 47 | 66 | 44 | 54 | 27 | 52 | 0.0100 |
| LOC102160096 | 108 | 116 | 103 | 39 | 120 | 52 | 0.2753 |
| LOC102160118 | 91 | 98 | 103 | 127 | 74 | 51 | 0.0109 |
| LOC102160243 | 83 | 58 | 57 | 25 | 63 | 39 | 0.0945 |
| LOC102160321 | 61 | 60 | 92 | 52 | 74 | 69 | 0.3889 |
| LOC102160340 | 123 | 184 | 130 | 215 | 167 | 117 | 0.1847 |
| LOC102160389 | 614 | 405 | 476 | 110 | 315 | 128 | 0.2076 |
| LOC102160506 | 61 | 36 | 53 | 22 | 78 | 43 | 0.0250 |
| LOC102160517 | 133 | 116 | 156 | 124 | 109 | 117 | 0.0218 |
| LOC102160522 | 52 | 35 | 17 | 81 | 24 | 129 | 0.0781 |
| LOC102160564 | 218 | 158 | 217 | 146 | 142 | 122 | 0.0417 |
| LOC102160572 | 55 | 78 | 54 | 30 | 52 | 41 | 0.1887 |
| LOC102160630 | 73 | 63 | 72 | 13 | 64 | 53 | 0.0313 |
| LOC102160652 | 137 | 121 | 115 | 150 | 107 | 143 | 0.1743 |
| LOC102160712 | 144 | 102 | 137 | 64 | 108 | 83 | 0.2697 |
| LOC102160748 | 443 | 586 | 292 | 556 | 363 | 425 | 0.0433 |
| LOC102161115 | 1682 | 1131 | 2080 | 990 | 1543 | 869 | 0.0760 |
| LOC102161477 | 57 | 42 | 36 | 22 | 34 | 18 | 0.1302 |
| LOC102161480 | 35 | 30 | 21 | 26 | 54 | 25 | 0.0151 |
| LOC102161741 | 424 | 416 | 495 | 303 | 436 | 321 | 0.0201 |
| LOC102161744 | 32 | 48 | 59 | 38 | 88 | 55 | 0.2497 |
| LOC102161888 | 226 | 193 | 240 | 159 | 180 | 150 | 0.0446 |
| LOC102161909 | 654 | 646 | 783 | 415 | 866 | 407 | 0.1605 |
| LOC102161912 | 62 | 48 | 66 | 34 | 50 | 54 | 0.2327 |
| LOC102161921 | 1237 | 464 | 1289 | 249 | 918 | 466 | 0.0676 |
| LOC102161969 | 38 | 72 | 50 | 72 | 67 | 56 | 0.0482 |
| LOC102161978 | 251 | 177 | 282 | 275 | 307 | 250 | 0.0566 |
| LOC102161992 | 39 | 56 | 69 | 35 | 47 | 45 | 0.2004 |
| LOC102162007 | 192 | 160 | 120 | 187 | 136 | 168 | 0.0252 |
| LOC102162074 | 458 | 296 | 759 | 270 | 701 | 284 | 0.1454 |
| LOC102162209 | 9183 | 7713 | 11303 | 3494 | 6985 | 4236 | 0.0487 |
| LOC102162214 | 89 | 72 | 107 | 44 | 69 | 58 | 0.5705 |
| LOC102162288 | 42 | 79 | 68 | 29 | 51 | 53 | 0.0208 |
| LOC102162300 | 1063 | 619 | 716 | 260 | 498 | 216 | 0.2948 |
| LOC102162338 | 59 | 121 | 58 | 205 | 93 | 171 | 0.2651 |
| LOC102162389 | 99 | 152 | 106 | 106 | 75 | 98 | 0.0352 |
| LOC102162428 | 82 | 95 | 130 | 48 | 85 | 58 | 0.3439 |
| LOC102162462 | 51 | 37 | 30 | 28 | 31 | 28 | 0.0171 |
| LOC102162488 | 1049 | 796 | 863 | 233 | 437 | 190 | 0.0993 |
| LOC102162566 | 190 | 243 | 135 | 105 | 130 | 119 | 0.1349 |
| LOC102162605 | 261 | 304 | 261 | 211 | 281 | 246 | 0.0544 |
| LOC102162623 | 126 | 128 | 114 | 110 | 95 | 79 | 0.0057 |
| LOC102162669 | 54 | 47 | 62 | 52 | 95 | 40 | 0.0410 |
| LOC102162684 | 165 | 136 | 178 | 128 | 128 | 110 | 0.0272 |
| LOC102162727 | 33 | 60 | 44 | 55 | 34 | 41 | 0.1920 |
| LOC102162878 | 19 | 59 | 39 | 33 | 38 | 36 | 0.1638 |
| LOC102162952 | 77 | 62 | 112 | 39 | 96 | 28 | 0.1428 |
| LOC102162978 | 47 | 50 | 89 | 65 | 92 | 60 | 0.0136 |
| LOC102163153 | 144 | 88 | 68 | 61 | 61 | 83 | 0.2280 |
| LOC102163267 | 569 | 516 | 887 | 461 | 923 | 536 | 0.1493 |
| LOC102163278 | 112 | 83 | 141 | 74 | 191 | 78 | 0.0646 |
| LOC102163402 | 58 | 36 | 50 | 47 | 68 | 45 | 0.0265 |
| LOC102163571 | 86 | 94 | 24 | 38 | 46 | 30 | 0.1177 |
| LOC102163670 | 76 | 80 | 62 | 73 | 82 | 71 | 0.0461 |
| LOC102163680 | 112 | 116 | 125 | 73 | 123 | 80 | 0.0271 |
| LOC102163685 | 111 | 87 | 111 | 47 | 81 | 54 | 0.0144 |
| LOC102163687 | 176 | 198 | 188 | 151 | 174 | 141 | 0.0367 |
| LOC102163793 | 24 | 15 | 23 | 50 | 10 | 31 | 0.1707 |
| LOC102163816 | 640 | 608 | 247 | 1322 | 493 | 712 | 0.2823 |
| LOC102163894 | 47 | 52 | 40 | 79 | 43 | 63 | 0.0348 |
| LOC102164066 | 123 | 161 | 160 | 113 | 132 | 133 | 0.1160 |
| LOC102164131 | 48 | 28 | 62 | 27 | 29 | 26 | 0.8837 |
| LOC102164161 | 79 | 104 | 117 | 108 | 119 | 90 | 0.9719 |
| LOC102164231 | 190 | 233 | 165 | 246 | 185 | 213 | 0.2367 |
| LOC102164323 | 27 | 19 | 39 | 23 | 80 | 38 | 0.4633 |
| LOC102164325 | 26 | 19 | 23 | 47 | 44 | 54 | 0.0477 |
| LOC102164379 | 75 | 41 | 120 | 48 | 57 | 57 | 0.0078 |
| LOC102164427 | 116 | 165 | 134 | 177 | 161 | 158 | 0.3454 |
| LOC102164588 | 168 | 158 | 196 | 99 | 161 | 127 | 0.0237 |
| LOC102164640 | 430 | 455 | 419 | 243 | 416 | 325 | 0.1433 |
| LOC102164657 | 59 | 60 | 79 | 38 | 44 | 58 | 0.0475 |
| LOC102164659 | 99 | 72 | 144 | 100 | 116 | 94 | 0.0132 |
| LOC102164693 | 705 | 680 | 880 | 328 | 781 | 284 | 0.1202 |
| LOC102164703 | 829 | 1235 | 1759 | 937 | 3899 | 1957 | 0.0629 |
| LOC102164818 | 187 | 209 | 145 | 95 | 197 | 109 | 0.3291 |
| LOC102164851 | 48 | 59 | 58 | 46 | 61 | 49 | 0.0226 |
| LOC102165023 | 141 | 133 | 207 | 85 | 143 | 122 | 0.0555 |
| LOC102165044 | 95 | 83 | 126 | 64 | 99 | 40 | 0.0175 |
| LOC102165098 | 38 | 67 | 49 | 33 | 61 | 55 | 0.0451 |
| LOC102165122 | 84 | 80 | 110 | 67 | 115 | 50 | 0.0447 |
| LOC102165211 | 390 | 457 | 377 | 370 | 359 | 326 | 0.3139 |
| LOC102165479 | 77 | 68 | 73 | 56 | 49 | 37 | 0.0275 |
| LOC102165633 | 548 | 608 | 592 | 608 | 528 | 488 | 0.1456 |
| LOC102165705 | 19 | 48 | 86 | 88 | 45 | 123 | 0.1042 |
| LOC102165730 | 57 | 93 | 47 | 91 | 41 | 78 | 0.1024 |
| LOC102165781 | 37 | 21 | 52 | 20 | 69 | 27 | 0.0114 |
| LOC102165783 | 96 | 78 | 146 | 81 | 64 | 59 | 0.0266 |
| LOC102165892 | 32 | 36 | 84 | 27 | 46 | 26 | 0.0866 |
| LOC102165926 | 42 | 28 | 47 | 18 | 26 | 54 | 0.0248 |
| LOC102165933 | 47 | 40 | 65 | 29 | 100 | 52 | 0.1246 |
| LOC102165975 | 36 | 18 | 55 | 27 | 86 | 17 | 0.1566 |
| LOC102166056 | 73 | 88 | 73 | 41 | 61 | 89 | 0.0914 |
| LOC102166108 | 46 | 36 | 44 | 14 | 52 | 26 | 0.0147 |
| LOC102166211 | 103 | 85 | 97 | 110 | 99 | 117 | 0.0257 |
| LOC102166340 | 53 | 66 | 26 | 49 | 26 | 45 | 0.4481 |
| LOC102166382 | 98 | 122 | 123 | 87 | 141 | 99 | 0.0145 |
| LOC102166393 | 65 | 58 | 31 | 102 | 60 | 48 | 0.1192 |
| LOC102166397 | 84 | 53 | 72 | 45 | 53 | 57 | 0.0296 |
| LOC102166500 | 308 | 226 | 337 | 143 | 388 | 164 | 0.2152 |
| LOC102166536 | 61 | 58 | 64 | 28 | 43 | 24 | 0.0462 |
| LOC102166710 | 47 | 40 | 45 | 33 | 50 | 18 | 0.2535 |
| LOC102166930 | 79 | 60 | 73 | 58 | 60 | 42 | 0.0802 |
| LOC102166957 | 36 | 61 | 24 | 28 | 37 | 50 | 0.3936 |
| LOC102167065 | 80 | 92 | 80 | 62 | 75 | 43 | 0.0686 |
| LOC102167177 | 83 | 51 | 73 | 71 | 106 | 56 | 0.0247 |
| LOC102167199 | 57 | 49 | 52 | 45 | 51 | 34 | 0.1991 |
| LOC102167294 | 61 | 79 | 75 | 55 | 65 | 52 | 0.2179 |
| LOC102167309 | 652 | 329 | 779 | 268 | 702 | 345 | 0.0329 |
| LOC102167359 | 319 | 401 | 300 | 472 | 279 | 275 | 0.0976 |
| LOC102167397 | 358 | 262 | 356 | 184 | 355 | 198 | 0.2952 |
| LOC102167472 | 76 | 56 | 30 | 51 | 44 | 38 | 0.1221 |
| LOC102167529 | 38 | 19 | 52 | 36 | 40 | 31 | 0.0517 |
| LOC102167554 | 110 | 79 | 142 | 27 | 92 | 48 | 0.0790 |
| LOC102167618 | 38 | 33 | 58 | 34 | 32 | 28 | 0.0515 |
| LOC102167692 | 92 | 51 | 46 | 66 | 65 | 50 | 0.0516 |
| LOC102167708 | 2 | 137 | 11 | 13 | 15 | 3 | 0.2319 |
| LOC102167727 | 658 | 605 | 763 | 473 | 743 | 495 | 0.1817 |
| LOC102167796 | 62 | 50 | 79 | 56 | 60 | 37 | 0.0257 |
| LOC102167899 | 71 | 54 | 70 | 37 | 45 | 36 | 0.0495 |
| LOC102167901 | 39 | 70 | 51 | 200 | 74 | 193 | 0.4916 |
| LOC102167943 | 82 | 59 | 97 | 55 | 91 | 75 | 0.2094 |
| LOC102168027 | 44 | 74 | 76 | 54 | 56 | 60 | 0.1808 |
| LOC102168077 | 88 | 105 | 72 | 94 | 88 | 66 | 0.4459 |
| LOC102168193 | 137 | 172 | 192 | 207 | 171 | 185 | 0.1522 |

a: Coding probability was predicated by CPC2

**Table S2 Pedication of subcellular localization of sow follicular lncRNAs**

| LncRNAs | Cytoplasm | Nucleus | Ribosome | Cytosol | Exosome | Subcellular localization |
| --- | --- | --- | --- | --- | --- | --- |
| LOC100152407 | 0.1371 | 0.6842 | 0.0611 | 0.0220 | 0.0955 | Nucleus |
| LOC100510918 | 0.1286 | 0.0804 | 0.0945 | 0.6460 | 0.0505 | Cytosol |
| LOC100510923 | 0.0630 | 0.0394 | 0.0805 | 0.7531 | 0.0640 | Cytosol |
| LOC100512560 | 0.2176 | 0.6385 | 0.0375 | 0.0242 | 0.0822 | Nucleus |
| LOC100512700 | 0.1133 | 0.1547 | 0.0353 | 0.0606 | 0.6361 | Exosome |
| LOC100512907 | 0.2190 | 0.7497 | 0.0056 | 0.0179 | 0.0078 | Nucleus |
| LOC100513133 | 0.6717 | 0.2251 | 0.0163 | 0.0324 | 0.0545 | Cytoplasm |
| LOC100513188 | 0.2481 | 0.1012 | 0.2334 | 0.2960 | 0.1213 | Cytosol |
| LOC100513397 | 0.8810 | 0.0630 | 0.0147 | 0.0333 | 0.0080 | Cytoplasm |
| LOC100514147 | 0.2993 | 0.1408 | 0.2932 | 0.2552 | 0.0115 | Cytoplasm |
| LOC100514807 | 0.1938 | 0.6471 | 0.0208 | 0.1061 | 0.0321 | Nucleus |
| LOC100514834 | 0.3329 | 0.4371 | 0.0812 | 0.0869 | 0.0619 | Nucleus |
| LOC100514924 | 0.2929 | 0.1340 | 0.0626 | 0.4552 | 0.0553 | Cytosol |
| LOC100515112 | 0.7331 | 0.1077 | 0.0291 | 0.0994 | 0.0307 | Cytoplasm |
| LOC100515492 | 0.6615 | 0.0517 | 0.0206 | 0.2431 | 0.0232 | Cytoplasm |
| LOC100515792 | 0.0475 | 0.0188 | 0.1447 | 0.7560 | 0.0329 | Cytosol |
| LOC100516105 | 0.1833 | 0.2284 | 0.1139 | 0.0964 | 0.3779 | Exosome |
| LOC100516145 | 0.4335 | 0.1216 | 0.0745 | 0.3618 | 0.0086 | Cytoplasm |
| LOC100516649 | 0.5662 | 0.1918 | 0.0846 | 0.1069 | 0.0504 | Nucleus |
| LOC100516669 | 0.1108 | 0.1684 | 0.5088 | 0.1810 | 0.0310 | Ribosome |
| LOC100516731 | 0.8848 | 0.0231 | 0.0192 | 0.0711 | 0.0018 | Cytoplasm |
| LOC100517116 | 0.1660 | 0.3075 | 0.2037 | 0.3120 | 0.0106 | Cytosol |
| LOC100517727 | 0.2289 | 0.0592 | 0.0150 | 0.5931 | 0.1038 | Cytosol |
| LOC100518120 | 0.4644 | 0.1308 | 0.0806 | 0.1560 | 0.1683 | Cytoplasm |
| LOC100519283 | 0.3177 | 0.3046 | 0.0447 | 0.1828 | 0.1502 | Cytoplasm |
| LOC100520275 | 0.4860 | 0.3182 | 0.0704 | 0.0980 | 0.0274 | Cytoplasm |
| LOC100520518 | 0.1591 | 0.0225 | 0.0532 | 0.2649 | 0.5003 | Exosome |
| LOC100521322 | 0.3093 | 0.2293 | 0.0192 | 0.4201 | 0.0222 | Cytosol |
| LOC100521518 | 0.2952 | 0.1841 | 0.1856 | 0.1331 | 0.2021 | Cytoplasm |
| LOC100521937 | 0.4576 | 0.4997 | 0.0056 | 0.0183 | 0.0187 | Nucleus |
| LOC100522143 | 0.2196 | 0.2031 | 0.0339 | 0.3115 | 0.2320 | Cytosol |
| LOC100522281 | 0.0599 | 0.0063 | 0.5765 | 0.3240 | 0.0334 | Ribosome |
| LOC100522818 | 0.4146 | 0.4438 | 0.0178 | 0.0395 | 0.0843 | Nucleus |
| LOC100522935 | 0.0950 | 0.3759 | 0.2667 | 0.2373 | 0.0251 | Nucleus |
| LOC100523870 | 0.1396 | 0.8315 | 0.0022 | 0.0097 | 0.0170 | Nucleus |
| LOC100524915 | 0.2056 | 0.3365 | 0.0962 | 0.0822 | 0.2795 | Nucleus |
| LOC100524923 | 0.1634 | 0.0844 | 0.0146 | 0.6507 | 0.0870 | Cytosol |
| LOC100525078 | 0.0576 | 0.0935 | 0.0054 | 0.8174 | 0.0261 | Cytosol |
| LOC100525571 | 0.2919 | 0.1005 | 0.3422 | 0.2374 | 0.0279 | Ribosome |
| LOC100525590 | 0.1173 | 0.5228 | 0.2517 | 0.0775 | 0.0308 | Nucleus |
| LOC100525935 | 0.1012 | 0.8575 | 0.0041 | 0.0051 | 0.0321 | Nucleus |
| LOC100526084 | 0.4343 | 0.2682 | 0.0908 | 0.1577 | 0.0490 | Cytoplasm |
| LOC100621235 | 0.5071 | 0.0677 | 0.0637 | 0.2409 | 0.1207 | Exosome |
| LOC100621455 | 0.0379 | 0.9334 | 0.0038 | 0.0051 | 0.0198 | Nucleus |
| LOC100622037 | 0.0707 | 0.0238 | 0.0361 | 0.7907 | 0.0786 | Cytosol |
| LOC100622481 | 0.2351 | 0.5829 | 0.0405 | 0.0521 | 0.0894 | Nucleus |
| LOC100623096 | 0.0818 | 0.0294 | 0.2382 | 0.6367 | 0.0139 | Cytosol |
| LOC100624113 | 0.1113 | 0.0228 | 0.1757 | 0.1959 | 0.4944 | Exosome |
| LOC100624406 | 0.8992 | 0.0276 | 0.0137 | 0.0569 | 0.0026 | Cytoplasm |
| LOC100625214 | 0.1397 | 0.0373 | 0.0746 | 0.6432 | 0.1052 | Cytosol |
| LOC100626258 | 0.1483 | 0.6035 | 0.1049 | 0.0569 | 0.0864 | Nucleus |
| LOC100626841 | 0.4038 | 0.4665 | 0.0155 | 0.0570 | 0.0573 | Nucleus |
| LOC100627868 | 0.2021 | 0.7246 | 0.0085 | 0.0223 | 0.0424 | Nucleus |
| LOC100627892 | 0.5694 | 0.3537 | 0.0121 | 0.0274 | 0.0374 | Cytoplasm |
| LOC100627949 | 0.4934 | 0.3235 | 0.0159 | 0.1100 | 0.0572 | Cytoplasm |
| LOC100628140 | 0.0148 | 0.0042 | 0.0246 | 0.9056 | 0.0508 | Cytosol |
| LOC100736638 | 0.0392 | 0.0088 | 0.0349 | 0.7912 | 0.1258 | Cytosol |
| LOC100736972 | 0.7489 | 0.1986 | 0.0118 | 0.0253 | 0.0154 | Cytoplasm |
| LOC100737186 | 0.8948 | 0.0308 | 0.0193 | 0.0532 | 0.0020 | Cytoplasm |
| LOC100737511 | 0.1911 | 0.7716 | 0.0033 | 0.0118 | 0.0221 | Nucleus |
| LOC100737961 | 0.6613 | 0.0586 | 0.0376 | 0.0730 | 0.1694 | Cytoplasm |
| LOC100738812 | 0.0320 | 0.0223 | 0.0446 | 0.8727 | 0.0285 | Cytosol |
| LOC100739121 | 0.1997 | 0.0502 | 0.0247 | 0.6214 | 0.1040 | Cytosol |
| LOC100739565 | 0.0650 | 0.0116 | 0.0132 | 0.8501 | 0.0602 | Cytosol |
| LOC100739688 | 0.3917 | 0.5469 | 0.0112 | 0.0394 | 0.0108 | Nucleus |
| LOC100739791 | 0.1705 | 0.0317 | 0.1085 | 0.3301 | 0.3593 | Exosome |
| LOC102157424 | 0.0410 | 0.0060 | 0.0103 | 0.8990 | 0.0437 | Cytosol |
| LOC102157454 | 0.2116 | 0.0705 | 0.0198 | 0.5689 | 0.1293 | Cytosol |
| LOC102157484 | 0.5074 | 0.3953 | 0.0095 | 0.0378 | 0.0500 | Cytoplasm |
| LOC102157546 | 0.0263 | 0.0061 | 0.0680 | 0.8820 | 0.0175 | Cytosol |
| LOC102157581 | 0.3714 | 0.0731 | 0.1458 | 0.1825 | 0.2272 | Cytoplasm |
| LOC102157630 | 0.8889 | 0.0772 | 0.0114 | 0.0178 | 0.0047 | Cytoplasm |
| LOC102157665 | 0.0505 | 0.0171 | 0.0108 | 0.9001 | 0.0215 | Cytosol |
| LOC102157672 | 0.4396 | 0.0618 | 0.1286 | 0.1960 | 0.1741 | Cytoplasm |
| LOC102157709 | 0.3552 | 0.2269 | 0.1603 | 0.2267 | 0.0310 | Cytoplasm |
| LOC102157766 | 0.7984 | 0.0623 | 0.0261 | 0.0689 | 0.0443 | Cytoplasm |
| LOC102157854 | 0.4963 | 0.0572 | 0.0575 | 0.2814 | 0.1076 | Cytoplasm |
| LOC102157897 | 0.6398 | 0.1824 | 0.0321 | 0.0923 | 0.0533 | Cytoplasm |
| LOC102157946 | 0.2093 | 0.1532 | 0.3353 | 0.2042 | 0.0979 | Ribosome |
| LOC102158087 | 0.6040 | 0.1312 | 0.0770 | 0.0390 | 0.1488 | Cytoplasm |
| LOC102158315 | 0.2091 | 0.0427 | 0.4279 | 0.3024 | 0.0180 | Ribosome |
| LOC102158334 | 0.0967 | 0.0152 | 0.0480 | 0.8155 | 0.0246 | Cytosol |
| LOC102158335 | 0.2607 | 0.1872 | 0.2980 | 0.1468 | 0.1073 | Ribosome |
| LOC102158363 | 0.5903 | 0.1300 | 0.0610 | 0.0893 | 0.1294 | Cytoplasm |
| LOC102158467 | 0.2977 | 0.4119 | 0.0867 | 0.0921 | 0.1117 | Nucleus |
| LOC102158524 | 0.8036 | 0.0796 | 0.0234 | 0.0830 | 0.0104 | Cytoplasm |
| LOC102158553 | 0.3718 | 0.4582 | 0.0104 | 0.0900 | 0.0697 | Nucleus |
| LOC102158687 | 0.5728 | 0.3445 | 0.0109 | 0.0126 | 0.0591 | Cytoplasm |
| LOC102158750 | 0.8871 | 0.0278 | 0.0220 | 0.0619 | 0.0011 | Cytoplasm |
| LOC102158821 | 0.6638 | 0.0643 | 0.0684 | 0.1904 | 0.0131 | Cytoplasm |
| LOC102158857 | 0.0723 | 0.0149 | 0.0697 | 0.7268 | 0.1162 | Cytosol |
| LOC102158889 | 0.1930 | 0.0797 | 0.1693 | 0.4526 | 0.1053 | Cytosol |
| LOC102159050 | 0.3550 | 0.0687 | 0.0801 | 0.4609 | 0.0353 | Cytosol |
| LOC102159054 | 0.1418 | 0.0068 | 0.1378 | 0.6768 | 0.0369 | Cytosol |
| LOC102159128 | 0.8242 | 0.1349 | 0.0108 | 0.0197 | 0.0104 | Cytoplasm |
| LOC102159203 | 0.1156 | 0.5589 | 0.0085 | 0.0195 | 0.2975 | Nucleus |
| LOC102159434 | 0.1674 | 0.1334 | 0.0579 | 0.5621 | 0.0792 | Cytosol |
| LOC102159502 | 0.4504 | 0.4517 | 0.0090 | 0.0427 | 0.0462 | Nucleus |
| LOC102159588 | 0.3925 | 0.0758 | 0.0809 | 0.4263 | 0.0245 | Cytosol |
| LOC102159607 | 0.8860 | 0.0176 | 0.0190 | 0.0757 | 0.0017 | Cytoplasm |
| LOC102159627 | 0.6472 | 0.2832 | 0.0147 | 0.0382 | 0.0167 | Cytoplasm |
| LOC102159645 | 0.9076 | 0.0488 | 0.0173 | 0.0235 | 0.0028 | Cytoplasm |
| LOC102159707 | 0.3044 | 0.4800 | 0.0078 | 0.1662 | 0.0416 | Nucleus |
| LOC102159960 | 0.4311 | 0.5221 | 0.0092 | 0.0161 | 0.0215 | Nucleus |
| LOC102159985 | 0.7033 | 0.1673 | 0.0221 | 0.0522 | 0.0551 | Cytoplasm |
| LOC102160096 | 0.2425 | 0.7029 | 0.0075 | 0.0351 | 0.0120 | Nucleus |
| LOC102160118 | 0.0769 | 0.1997 | 0.3802 | 0.2938 | 0.0494 | Ribosome |
| LOC102160243 | 0.2285 | 0.2285 | 0.0257 | 0.1502 | 0.3670 | Exosome |
| LOC102160321 | 0.0196 | 0.0136 | 0.0319 | 0.9117 | 0.0232 | Cytosol |
| LOC102160340 | 0.0538 | 0.0078 | 0.0791 | 0.5588 | 0.3005 | Cytosol |
| LOC102160389 | 0.8963 | 0.0345 | 0.0186 | 0.0450 | 0.0055 | Cytoplasm |
| LOC102160506 | 0.1144 | 0.8662 | 0.0015 | 0.0087 | 0.0092 | Nucleus |
| LOC102160517 | 0.1922 | 0.2282 | 0.2443 | 0.2343 | 0.1010 | Ribosome |
| LOC102160522 | 0.2316 | 0.7129 | 0.0096 | 0.0165 | 0.0293 | Nucleus |
| LOC102160564 | 0.6482 | 0.1358 | 0.0487 | 0.0195 | 0.1478 | Cytoplasm |
| LOC102160572 | 0.7301 | 0.1690 | 0.0195 | 0.0354 | 0.0460 | Cytoplasm |
| LOC102160630 | 0.7621 | 0.0557 | 0.0328 | 0.0659 | 0.0836 | Cytoplasm |
| LOC102160652 | 0.0948 | 0.5218 | 0.0977 | 0.1253 | 0.1603 | Nucleus |
| LOC102160712 | 0.0295 | 0.0115 | 0.0119 | 0.9034 | 0.0438 | Cytosol |
| LOC102160748 | 0.7033 | 0.2460 | 0.0119 | 0.0326 | 0.0062 | Cytoplasm |
| LOC102161115 | 0.2168 | 0.5436 | 0.0150 | 0.0623 | 0.1623 | Nucleus |
| LOC102161477 | 0.0972 | 0.0216 | 0.0735 | 0.5716 | 0.2360 | Cytosol |
| LOC102161480 | 0.7722 | 0.1213 | 0.0171 | 0.0420 | 0.0474 | Cytoplasm |
| LOC102161741 | 0.3788 | 0.1007 | 0.1831 | 0.2864 | 0.0510 | Cytoplasm |
| LOC102161744 | 0.2241 | 0.0270 | 0.0489 | 0.6282 | 0.0717 | Cytosol |
| LOC102161888 | 0.3493 | 0.0381 | 0.0815 | 0.5055 | 0.0255 | Cytosol |
| LOC102161909 | 0.0689 | 0.0171 | 0.1589 | 0.7455 | 0.0096 | Cytosol |
| LOC102161912 | 0.2169 | 0.1401 | 0.0198 | 0.4351 | 0.1881 | Cytosol |
| LOC102161921 | 0.3929 | 0.1514 | 0.0193 | 0.4143 | 0.0221 | Cytosol |
| LOC102161969 | 0.9054 | 0.0321 | 0.0133 | 0.0469 | 0.0023 | Cytoplasm |
| LOC102161978 | 0.4123 | 0.1028 | 0.0981 | 0.3050 | 0.0818 | Cytoplasm |
| LOC102161992 | 0.3035 | 0.4978 | 0.0324 | 0.0239 | 0.1424 | Nucleus |
| LOC102162007 | 0.8898 | 0.0208 | 0.0180 | 0.0695 | 0.0019 | Cytoplasm |
| LOC102162074 | 0.0250 | 0.0241 | 0.0567 | 0.8505 | 0.0436 | Cytosol |
| LOC102162209 | 0.8955 | 0.0433 | 0.0116 | 0.0454 | 0.0043 | Cytoplasm |
| LOC102162214 | 0.0951 | 0.0189 | 0.0877 | 0.4233 | 0.3749 | Cytosol |
| LOC102162288 | 0.6914 | 0.0838 | 0.0633 | 0.0312 | 0.1303 | Cytoplasm |
| LOC102162300 | 0.6382 | 0.2084 | 0.0285 | 0.0708 | 0.0541 | Cytoplasm |
| LOC102162338 | 0.8496 | 0.1042 | 0.0152 | 0.0185 | 0.0125 | Cytoplasm |
| LOC102162389 | 0.2248 | 0.0622 | 0.3434 | 0.2406 | 0.1290 | Ribosome |
| LOC102162428 | 0.2499 | 0.1471 | 0.2944 | 0.0885 | 0.2201 | Ribosome |
| LOC102162462 | 0.3625 | 0.1375 | 0.2210 | 0.1425 | 0.1365 | Cytoplasm |
| LOC102162488 | 0.8003 | 0.1297 | 0.0087 | 0.0543 | 0.0070 | Cytoplasm |
| LOC102162566 | 0.6422 | 0.2677 | 0.0142 | 0.0488 | 0.0271 | Cytoplasm |
| LOC102162605 | 0.4111 | 0.4525 | 0.0123 | 0.0155 | 0.1085 | Nucleus |
| LOC102162623 | 0.1372 | 0.3604 | 0.2071 | 0.2542 | 0.0411 | Nucleus |
| LOC102162669 | 0.2547 | 0.1363 | 0.1232 | 0.3010 | 0.1847 | Cytosol |
| LOC102162684 | 0.7245 | 0.1191 | 0.0414 | 0.0655 | 0.0496 | Cytoplasm |
| LOC102162727 | 0.2892 | 0.1541 | 0.1069 | 0.3953 | 0.0546 | Cytosol |
| LOC102162878 | 0.2200 | 0.0616 | 0.3558 | 0.3418 | 0.0207 | Ribosome |
| LOC102162952 | 0.8799 | 0.0245 | 0.0251 | 0.0640 | 0.0065 | Cytoplasm |
| LOC102162978 | 0.3543 | 0.3676 | 0.0117 | 0.0329 | 0.2334 | Nucleus |
| LOC102163153 | 0.8237 | 0.0615 | 0.0152 | 0.0962 | 0.0034 | Cytoplasm |
| LOC102163267 | 0.7341 | 0.1721 | 0.0136 | 0.0658 | 0.0144 | Cytoplasm |
| LOC102163278 | 0.0859 | 0.0132 | 0.0968 | 0.6772 | 0.1269 | Cytosol |
| LOC102163402 | 0.5406 | 0.0499 | 0.0360 | 0.3263 | 0.0472 | Cytoplasm |
| LOC102163571 | 0.0262 | 0.0197 | 0.0258 | 0.8531 | 0.0751 | Cytosol |
| LOC102163670 | 0.2760 | 0.5521 | 0.0070 | 0.0858 | 0.0791 | Nucleus |
| LOC102163680 | 0.4411 | 0.2949 | 0.0464 | 0.1025 | 0.1151 | Cytoplasm |
| LOC102163685 | 0.8042 | 0.1501 | 0.0131 | 0.0200 | 0.0126 | Cytoplasm |
| LOC102163687 | 0.5094 | 0.0624 | 0.0542 | 0.1750 | 0.1990 | Cytoplasm |
| LOC102163793 | 0.2654 | 0.1693 | 0.0557 | 0.2308 | 0.2789 | Exosome |
| LOC102163816 | 0.2137 | 0.0640 | 0.2158 | 0.4661 | 0.0404 | Cytosol |
| LOC102163894 | 0.6462 | 0.2562 | 0.0199 | 0.0632 | 0.0144 | Cytoplasm |
| LOC102164066 | 0.8537 | 0.0778 | 0.0152 | 0.0396 | 0.0137 | Cytoplasm |
| LOC102164131 | 0.1776 | 0.2090 | 0.0704 | 0.0139 | 0.5291 | Exosome |
| LOC102164161 | 0.0831 | 0.0126 | 0.2037 | 0.3067 | 0.3940 | Exosome |
| LOC102164231 | 0.3635 | 0.5470 | 0.0066 | 0.0582 | 0.0246 | Nucleus |
| LOC102164323 | 0.0307 | 0.0052 | 0.0431 | 0.8208 | 0.1001 | Cytosol |
| LOC102164325 | 0.3703 | 0.3833 | 0.0915 | 0.1344 | 0.0205 | Nucleus |
| LOC102164379 | 0.3968 | 0.1541 | 0.1662 | 0.0646 | 0.2183 | Cytoplasm |
| LOC102164427 | 0.0231 | 0.0084 | 0.0100 | 0.9314 | 0.0271 | Cytosol |
| LOC102164588 | 0.4486 | 0.2083 | 0.0533 | 0.0377 | 0.2521 | Cytoplasm |
| LOC102164640 | 0.8672 | 0.0686 | 0.0109 | 0.0495 | 0.0038 | Cytoplasm |
| LOC102164657 | 0.5311 | 0.3853 | 0.0152 | 0.0321 | 0.0364 | Cytoplasm |
| LOC102164659 | 0.1288 | 0.1075 | 0.1255 | 0.1844 | 0.4539 | Exosome |
| LOC102164693 | 0.1647 | 0.6492 | 0.0213 | 0.0339 | 0.1310 | Nucleus |
| LOC102164703 | 0.6488 | 0.1216 | 0.0268 | 0.0711 | 0.1317 | Cytoplasm |
| LOC102164818 | 0.2638 | 0.0419 | 0.0409 | 0.6477 | 0.0056 | Cytosol |
| LOC102164851 | 0.6712 | 0.1994 | 0.0223 | 0.0898 | 0.0174 | Cytoplasm |
| LOC102165023 | 0.8948 | 0.0349 | 0.0138 | 0.0550 | 0.0015 | Cytoplasm |
| LOC102165044 | 0.8127 | 0.1217 | 0.0208 | 0.0258 | 0.0191 | Cytoplasm |
| LOC102165098 | 0.9038 | 0.0469 | 0.0127 | 0.0332 | 0.0034 | Cytoplasm |
| LOC102165122 | 0.6100 | 0.1780 | 0.0376 | 0.1498 | 0.0247 | Cytoplasm |
| LOC102165211 | 0.0094 | 0.0039 | 0.0089 | 0.9679 | 0.0099 | Cytosol |
| LOC102165479 | 0.7894 | 0.1314 | 0.0230 | 0.0266 | 0.0296 | Cytoplasm |
| LOC102165633 | 0.8864 | 0.0426 | 0.0124 | 0.0566 | 0.0021 | Cytoplasm |
| LOC102165705 | 0.7758 | 0.0236 | 0.0196 | 0.1765 | 0.0045 | Cytoplasm |
| LOC102165730 | 0.1591 | 0.0280 | 0.0763 | 0.7161 | 0.0205 | Cytosol |
| LOC102165781 | 0.2732 | 0.0818 | 0.3408 | 0.2459 | 0.0583 | Ribosome |
| LOC102165783 | 0.0468 | 0.0235 | 0.5838 | 0.3347 | 0.0113 | Ribosome |
| LOC102165892 | 0.2038 | 0.0376 | 0.2144 | 0.5326 | 0.0116 | Cytosol |
| LOC102165926 | 0.4720 | 0.4207 | 0.0114 | 0.0235 | 0.0723 | Cytoplasm |
| LOC102165933 | 0.1380 | 0.0521 | 0.2470 | 0.4280 | 0.1349 | Cytosol |
| LOC102165975 | 0.2001 | 0.0270 | 0.1240 | 0.5643 | 0.0845 | Cytosol |
| LOC102166056 | 0.9049 | 0.0416 | 0.0137 | 0.0375 | 0.0022 | Cytoplasm |
| LOC102166108 | 0.5463 | 0.2678 | 0.0377 | 0.0825 | 0.0656 | Cytoplasm |
| LOC102166211 | 0.5155 | 0.2199 | 0.0879 | 0.0986 | 0.0781 | Cytoplasm |
| LOC102166340 | 0.1998 | 0.1931 | 0.2604 | 0.1369 | 0.2098 | Ribosome |
| LOC102166382 | 0.6694 | 0.1330 | 0.0221 | 0.0369 | 0.1386 | Cytoplasm |
| LOC102166393 | 0.3654 | 0.4672 | 0.0371 | 0.1003 | 0.0300 | Nucleus |
| LOC102166397 | 0.5022 | 0.2135 | 0.0610 | 0.2102 | 0.0132 | Cytoplasm |
| LOC102166500 | 0.3144 | 0.4376 | 0.0102 | 0.1612 | 0.0766 | Nucleus |
| LOC102166536 | 0.8394 | 0.0943 | 0.0241 | 0.0354 | 0.0068 | Nucleus |
| LOC102166710 | 0.0389 | 0.0071 | 0.0404 | 0.8699 | 0.0436 | Cytosol |
| LOC102166930 | 0.5185 | 0.1734 | 0.0484 | 0.0531 | 0.2067 | Cytoplasm |
| LOC102166957 | 0.5265 | 0.2871 | 0.0174 | 0.1032 | 0.0658 | Cytoplasm |
| LOC102167065 | 0.8201 | 0.1512 | 0.0083 | 0.0154 | 0.0051 | Cytoplasm |
| LOC102167177 | 0.2587 | 0.6883 | 0.0056 | 0.0315 | 0.0159 | Nucleus |
| LOC102167199 | 0.3895 | 0.2452 | 0.0531 | 0.2652 | 0.0470 | Cytoplasm |
| LOC102167294 | 0.2829 | 0.2476 | 0.0124 | 0.0997 | 0.3574 | Exosome |
| LOC102167309 | 0.4427 | 0.3369 | 0.0481 | 0.0272 | 0.1451 | Cytoplasm |
| LOC102167359 | 0.0228 | 0.0133 | 0.0082 | 0.9314 | 0.0243 | Cytosol |
| LOC102167397 | 0.3129 | 0.0286 | 0.3366 | 0.1307 | 0.1911 | Ribosome |
| LOC102167472 | 0.4291 | 0.0448 | 0.0705 | 0.4254 | 0.0301 | Cytoplasm |
| LOC102167529 | 0.0153 | 0.0088 | 0.5674 | 0.3994 | 0.0091 | Ribosome |
| LOC102167554 | 0.8371 | 0.0758 | 0.0166 | 0.0471 | 0.0235 | Cytoplasm |
| LOC102167618 | 0.4803 | 0.1595 | 0.0285 | 0.1622 | 0.1695 | Cytoplasm |
| LOC102167692 | 0.0908 | 0.0112 | 0.1567 | 0.3676 | 0.3737 | Exosome |
| LOC102167708 | 0.3354 | 0.6173 | 0.0086 | 0.0226 | 0.0161 | Nucleus |
| LOC102167727 | 0.4196 | 0.1919 | 0.0507 | 0.2962 | 0.0415 | Cytoplasm |
| LOC102167796 | 0.6989 | 0.1826 | 0.0218 | 0.0509 | 0.0457 | Cytoplasm |
| LOC102167899 | 0.6753 | 0.2881 | 0.0070 | 0.0160 | 0.0135 | Cytoplasm |
| LOC102167901 | 0.3637 | 0.2001 | 0.0425 | 0.3299 | 0.0638 | Cytoplasm |
| LOC102167943 | 0.8880 | 0.0482 | 0.0192 | 0.0362 | 0.0084 | Cytoplasm |
| LOC102168027 | 0.8888 | 0.0408 | 0.0155 | 0.0486 | 0.0064 | Cytoplasm |
| LOC102168077 | 0.0233 | 0.0041 | 0.0176 | 0.9425 | 0.0125 | Cytosol |
| LOC102168193 | 0.3588 | 0.1059 | 0.1363 | 0.3489 | 0.0501 | Cytoplasm |

**Table S3 miRNAs potentially interacting with nuclear lncRNAs in sow GCs.**

| Nuclear lncRNAs | Potential miRNAs | MRE position | Minimum free energy/kcal/mol |
| --- | --- | --- | --- |
| LOC100512907 | miR-10a | 1245-1252 | -25.7 |
|  | miR-95 | 2320-2326 | -21.6 |
|  | miR-144 | 2711-2719 | -18.5 |
|  | miR-10b | 1246-1253 | -23.6 |
|  | miR-17 | 1071-1081 | -28.2 |
|  | miR-195-3p | 81-91 | -22.9 |
|  | miR-216 | 2585-2591 | -25.5 |
|  | miR-676 | 2544-2557 | -25.6 |
|  | miR-106a | 1070-1081 | -31.1 |
|  | miR-34a | 1399-1401 | -29.3 |
|  | miR-196 | 4223-4230 | -29.0 |
|  | miR-432 | 1055-1063 | -25.2 |
|  | miR-371 | 3674-3683 | -30.4 |
|  | miR-20a | 1071-1081 | -26.3 |
|  | miR-20b | 1071-1081 | -30.3 |
|  | miR-361 | 2451-2462 | -28.6 |
|  | miR-374a | 1855-1861 | -17.2 |
|  | miR-130a | 4886-4892 | -22.4 |
|  | miR-326 | 2874-2883 | -32.2 |
|  | miR-185 | 3018-3025 | -30.5 |
|  | miR-500 | 2751-2758 | -27.9 |
|  | miR-139 | 3244-3251 | -31.7 |
| LOC102160522 | miR-664 | 1692-1697 | -23.4 |
|  | miR-149 | 246-253 | -28.8 |
|  | miR-499 | 453-462 | -17.8 |
|  | miR-375 | 200-206 | -23.9 |
| LOC102164325 | miR-339 | 76-83 (MRE1) | -27.0 |
|  |  | 152-161 (MRE2) | -30.7 |
|  |  | 107-176 (MRE3) | -19.2 |
|  | miR-331 | 261-267 | -22.5 |
| LOC100626841 | miR-24 | 717-723 | -24.5 |
|  | miR-378 | 746-753 | -32.7 |

**Table S4 The relative binding activity between nuclear lncRNAs and their corresponding miRNAs.**

| Nuclear lncRNAs | Corresponding miRNAs | Relative binding activitya |
| --- | --- | --- |
| LOC100512907 | miR-144 | 0.6506 |
| LOC100626841 | miR-378 | 0.8288 |
| LOC102164325 | miR-339 | 37.4793 |
| LOC102160522 | miR-375 | 0.0025 |

a: The relative binding activity is the ratio of the level of miRNAs that interact with lncRNAs pulled down by the probe to the level of lncRNAs that pulled down by the probe.

**Table S5 miRNAs in the nucleus potentially interacting with nuclear lncRNAs**

| Cell types | Nuclear lncRNAs | miRNAs | MRE-start | MRE-end | Minimum free energy kcal/mol | Algorithms |
| --- | --- | --- | --- | --- | --- | --- |
| A549 | LINC01600 | hsa-miR-326 | 685 | 704 | -34.52 | miRanda,TargetScan,RNAhybrid |
| A549 | LINC01600 | hsa-miR-326 | 622 | 641 | -34.52 | miRanda,TargetScan,RNAhybrid |
| A549 | LINC01270 | hsa-miR-326 | 60 | 79 | -34.19 | miRanda,TargetScan,RNAhybrid |
| A549 | LINC01270 | hsa-miR-326 | 49 | 68 | -34.19 | miRanda,TargetScan,RNAhybrid |
| A549 | LINC01270 | hsa-miR-326 | 20 | 39 | -34.19 | miRanda,TargetScan,RNAhybrid |
| A549 | MEG3 | hsa-miR-331-3p | 235 | 255 | -31.44 | miRanda,TargetScan,RNAhybrid |
| A549 | DIO3OS | hsa-miR-331-3p | 188 | 208 | -28.62 | miRanda,TargetScan,RNAhybrid |
| A549 | DIO3OS | hsa-miR-331-3p | 274 | 294 | -28.62 | miRanda,TargetScan,RNAhybrid |
| A549 | FAM157C | hsa-miR-331-3p | 1169 | 1189 | -30.43 | miRanda,TargetScan,RNAhybrid |
| A549 | FAM157C | hsa-miR-331-3p | 1006 | 1026 | -30.43 | miRanda,TargetScan,RNAhybrid |
| A549 | FAM157C | hsa-miR-331-3p | 1111 | 1131 | -30.43 | miRanda,TargetScan,RNAhybrid |
| A549 | FAM157C | hsa-miR-331-3p | 490 | 510 | -30.43 | miRanda,TargetScan,RNAhybrid |
| A549 | FAM157C | hsa-miR-331-3p | 21 | 41 | -30.43 | miRanda,TargetScan,RNAhybrid |
| A549 | LINC00511 | hsa-miR-331-3p | 201 | 220 | -27.07 | miRanda,TargetScan,RNAhybrid |
| A549 | LINC00511 | hsa-miR-331-3p | 334 | 353 | -27.07 | miRanda,TargetScan,RNAhybrid |
| A549 | LINC00511 | hsa-miR-331-3p | 122 | 141 | -27.07 | miRanda,TargetScan,RNAhybrid |
| A549 | LINC00511 | hsa-miR-331-3p | 473 | 492 | -27.07 | miRanda,TargetScan,RNAhybrid |
| A549 | LINC00511 | hsa-miR-331-3p | 595 | 614 | -27.07 | miRanda,TargetScan,RNAhybrid |
| A549 | NARF-IT1 | hsa-miR-331-3p | 281 | 301 | -36.15 | miRanda,TargetScan,RNAhybrid |
| A549 | LINC00313 | hsa-miR-331-3p | 208 | 228 | -30.15 | miRanda,TargetScan,RNAhybrid |
| A549 | LINC00313 | hsa-miR-331-3p | 1138 | 1158 | -30.15 | miRanda,TargetScan,RNAhybrid |
| A549 | LINC00313 | hsa-miR-331-3p | 592 | 612 | -30.15 | miRanda,TargetScan,RNAhybrid |
| A549 | MIR222HG | hsa-miR-574-5p | 290 | 312 | -31.92 | miRanda,TargetScan,RNAhybrid |
| A549 | PTOV1-AS2 | hsa-miR-612 | 205 | 234 | -37.47 | miRanda,TargetScan,RNAhybrid |
| A549 | PTOV1-AS2 | hsa-miR-612 | 216 | 245 | -37.47 | miRanda,TargetScan,RNAhybrid |
| A549 | LINC00313 | hsa-miR-650 | 1560 | 1580 | -34.19 | miRanda,TargetScan,RNAhybrid |
| A549 | LINC01126 | hsa-miR-663a | 459 | 481 | -37.78 | miRanda,TargetScan,RNAhybrid |
| A549 | A1BG-AS1 | hsa-miR-1204 | 67 | 89 | -32.56 | miRanda,TargetScan,RNAhybrid |
| A549 | A1BG-AS1 | hsa-miR-1204 | 81 | 101 | -23.54 | miRanda,TargetScan,RNAhybrid |
| A549 | LINC01118 | hsa-miR-1303 | 77 | 98 | -37.75 | miRanda,TargetScan,RNAhybrid |
| A549 | LINC01118 | hsa-miR-1303 | 60 | 81 | -37.75 | miRanda,TargetScan,RNAhybrid |
| A549 | SNHG4 | hsa-miR-3170 | 252 | 273 | -27.25 | miRanda,TargetScan,RNAhybrid |
| A549 | SNHG12 | hsa-miR-3178 | 297 | 313 | -31.38 | miRanda,TargetScan,RNAhybrid |
| A549 | SNHG12 | hsa-miR-3178 | 441 | 457 | -31.38 | miRanda,TargetScan,RNAhybrid |
| A549 | SNHG12 | hsa-miR-3178 | 544 | 560 | -31.38 | miRanda,TargetScan,RNAhybrid |
| A549 | SNHG12 | hsa-miR-3178 | 612 | 628 | -31.38 | miRanda,TargetScan,RNAhybrid |
| A549 | SNHG12 | hsa-miR-3178 | 724 | 740 | -31.38 | miRanda,TargetScan,RNAhybrid |
| A549 | SNHG12 | hsa-miR-3178 | 932 | 948 | -31.38 | miRanda,TargetScan,RNAhybrid |
| A549 | SNHG12 | hsa-miR-3178 | 1255 | 1271 | -31.38 | miRanda,TargetScan,RNAhybrid |
| A549 | SNHG12 | hsa-miR-3178 | 213 | 229 | -31.38 | miRanda,TargetScan,RNAhybrid |
| A549 | SNHG12 | hsa-miR-3178 | 47 | 63 | -31.38 | miRanda,TargetScan,RNAhybrid |
| A549 | SNHG12 | hsa-miR-3178 | 186 | 202 | -31.38 | miRanda,TargetScan,RNAhybrid |
| A549 | SNHG12 | hsa-miR-3178 | 194 | 210 | -31.38 | miRanda,TargetScan,RNAhybrid |
| A549 | SNHG12 | hsa-miR-3178 | 205 | 221 | -31.38 | miRanda,TargetScan,RNAhybrid |
| A549 | SNHG12 | hsa-miR-3178 | 206 | 222 | -31.38 | miRanda,TargetScan,RNAhybrid |
| A549 | SNHG12 | hsa-miR-3178 | 209 | 225 | -31.38 | miRanda,TargetScan,RNAhybrid |
| A549 | SNHG12 | hsa-miR-3178 | 224 | 240 | -31.38 | miRanda,TargetScan,RNAhybrid |
| A549 | SNHG12 | hsa-miR-3178 | 225 | 241 | -31.38 | miRanda,TargetScan,RNAhybrid |
| A549 | SNHG12 | hsa-miR-3178 | 230 | 246 | -31.38 | miRanda,TargetScan,RNAhybrid |
| A549 | SNHG12 | hsa-miR-3178 | 15 | 31 | -31.38 | miRanda,TargetScan,RNAhybrid |
| A549 | SNHG12 | hsa-miR-3178 | 170 | 186 | -31.38 | miRanda,TargetScan,RNAhybrid |
| A549 | SNHG12 | hsa-miR-3178 | 149 | 165 | -31.38 | miRanda,TargetScan,RNAhybrid |
| A549 | SNHG12 | hsa-miR-3178 | 219 | 235 | -31.38 | miRanda,TargetScan,RNAhybrid |
| A549 | SNHG12 | hsa-miR-3178 | 223 | 239 | -31.38 | miRanda,TargetScan,RNAhybrid |
| A549 | CAHM | hsa-miR-3178 | 35 | 53 | -32.32 | miRanda,TargetScan,RNAhybrid |
| A549 | LINC00605 | hsa-miR-3178 | 102 | 118 | -33.1 | miRanda,TargetScan,RNAhybrid |
| A549 | LINC00605 | hsa-miR-3178 | 159 | 175 | -33.1 | miRanda,TargetScan,RNAhybrid |
| A549 | MMP25-AS1 | hsa-miR-3178 | 867 | 883 | -36.22 | miRanda,TargetScan,RNAhybrid |
| A549 | MMP25-AS1 | hsa-miR-3178 | 840 | 856 | -36.22 | miRanda,TargetScan,RNAhybrid |
| A549 | ASB16-AS1 | hsa-miR-3178 | 851 | 867 | -32.15 | miRanda,TargetScan,RNAhybrid |
| A549 | LINC00106 | hsa-miR-3178 | 111 | 127 | -28.79 | miRanda,TargetScan,RNAhybrid |
| A549 | PAX8-AS1 | hsa-miR-320e | 15 | 32 | -25.81 | miRanda,TargetScan,RNAhybrid |
| A549 | ZMIZ1-AS1 | hsa-miR-3195 | 601 | 617 | -35.09 | miRanda,TargetScan,RNAhybrid |
| A549 | NR2F2-AS1 | hsa-miR-3195 | 76 | 92 | -28.24 | miRanda,TargetScan,RNAhybrid |
| A549 | NR2F2-AS1 | hsa-miR-3195 | 26 | 42 | -28.24 | miRanda,TargetScan,RNAhybrid |
| A549 | NR2F2-AS1 | hsa-miR-3195 | 74 | 90 | -28.24 | miRanda,TargetScan,RNAhybrid |
| A549 | NR2F2-AS1 | hsa-miR-3195 | 55 | 71 | -28.24 | miRanda,TargetScan,RNAhybrid |
| A549 | NR2F2-AS1 | hsa-miR-3195 | 87 | 103 | -28.24 | miRanda,TargetScan,RNAhybrid |
| A549 | SNAI3-AS1 | hsa-miR-3195 | 75 | 91 | -31.24 | miRanda,TargetScan,RNAhybrid |
| A549 | GATA6-AS1 | hsa-miR-3195 | 119 | 136 | -35.28 | miRanda,TargetScan,RNAhybrid |
| A549 | LINC00894 | hsa-miR-3195 | 908 | 924 | -33.3 | miRanda,TargetScan,RNAhybrid |
| A549 | DNAH17-AS1 | hsa-miR-3197 | 182 | 204 | -33.96 | miRanda,TargetScan,RNAhybrid |
| A549 | DNAH17-AS1 | hsa-miR-3197 | 169 | 191 | -33.96 | miRanda,TargetScan,RNAhybrid |
| A549 | DNAH17-AS1 | hsa-miR-3197 | 140 | 162 | -33.96 | miRanda,TargetScan,RNAhybrid |
| A549 | DIO3OS | hsa-miR-3198 | 71 | 92 | -37.9 | miRanda,TargetScan,RNAhybrid |
| A549 | DIO3OS | hsa-miR-3198 | 152 | 173 | -37.9 | miRanda,TargetScan,RNAhybrid |
| A549 | DIO3OS | hsa-miR-3198 | 270 | 291 | -37.9 | miRanda,TargetScan,RNAhybrid |
| A549 | DIO3OS | hsa-miR-3198 | 281 | 302 | -37.9 | miRanda,TargetScan,RNAhybrid |
| A549 | DIO3OS | hsa-miR-3198 | 636 | 657 | -37.9 | miRanda,TargetScan,RNAhybrid |
| A549 | DIO3OS | hsa-miR-3198 | 722 | 743 | -37.9 | miRanda,TargetScan,RNAhybrid |
| A549 | HOXA11-AS | hsa-miR-4286 | 208 | 224 | -25.92 | miRanda,TargetScan,RNAhybrid |
| A549 | MMP25-AS1 | hsa-miR-4286 | 190 | 206 | -26.51 | miRanda,TargetScan,RNAhybrid |
| A549 | LINC00511 | hsa-miR-4286 | 282 | 298 | -32.18 | miRanda,TargetScan,RNAhybrid |
| A549 | LINC00511 | hsa-miR-4286 | 679 | 695 | -32.18 | miRanda,TargetScan,RNAhybrid |
| A549 | CACTIN-AS1 | hsa-miR-4286 | 425 | 441 | -29.48 | miRanda,TargetScan,RNAhybrid |
| A549 | CACTIN-AS1 | hsa-miR-4286 | 410 | 426 | -29.48 | miRanda,TargetScan,RNAhybrid |
| A549 | CACTIN-AS1 | hsa-miR-4286 | 406 | 422 | -29.48 | miRanda,TargetScan,RNAhybrid |
| A549 | CACTIN-AS1 | hsa-miR-4286 | 79 | 95 | -29.48 | miRanda,TargetScan,RNAhybrid |
| A549 | LINC01239 | hsa-miR-331-3p | 603 | 623 | -29.48 | miRanda,TargetScan,RNAhybrid |
| A549 | LINC01239 | hsa-miR-331-3p | 184 | 204 | -29.48 | miRanda,TargetScan,RNAhybrid |
| A549 | LINC01239 | hsa-miR-331-3p | 126 | 146 | -29.48 | miRanda,TargetScan,RNAhybrid |
| A549 | MEG8 | hsa-miR-331-3p | 235 | 255 | -31.44 | miRanda,TargetScan,RNAhybrid |
| GM12878 | LINC00298 | hsa-miR-326 | 54 | 73 | -32.05 | miRanda,TargetScan,RNAhybrid |
| GM12878 | LINC00298 | hsa-miR-331-3p | 199 | 219 | -29.5 | miRanda,TargetScan,RNAhybrid |
| GM12878 | DARS-AS1 | hsa-miR-331-3p | 199 | 219 | -25.99 | miRanda,TargetScan,RNAhybrid |
| GM12878 | DARS-AS1 | hsa-miR-331-3p | 172 | 192 | -25.99 | miRanda,TargetScan,RNAhybrid |
| GM12878 | DARS-AS1 | hsa-miR-331-3p | 171 | 191 | -25.99 | miRanda,TargetScan,RNAhybrid |
| GM12878 | DARS-AS1 | hsa-miR-331-3p | 155 | 175 | -25.99 | miRanda,TargetScan,RNAhybrid |
| GM12878 | DARS-AS1 | hsa-miR-331-3p | 129 | 149 | -25.99 | miRanda,TargetScan,RNAhybrid |
| GM12878 | DARS-AS1 | hsa-miR-331-3p | 14 | 34 | -25.99 | miRanda,TargetScan,RNAhybrid |
| GM12878 | CDKN2B-AS1 | hsa-miR-331-3p | 603 | 623 | -29.48 | miRanda,TargetScan,RNAhybrid |
| GM12878 | CDKN2B-AS1 | hsa-miR-331-3p | 184 | 204 | -29.48 | miRanda,TargetScan,RNAhybrid |
| GM12878 | CDKN2B-AS1 | hsa-miR-331-3p | 126 | 146 | -29.48 | miRanda,TargetScan,RNAhybrid |
| GM12878 | MEG3 | hsa-miR-331-3p | 235 | 255 | -31.44 | miRanda,TargetScan,RNAhybrid |
| GM12878 | NARF-IT1 | hsa-miR-331-3p | 281 | 301 | -36.15 | miRanda,TargetScan,RNAhybrid |
| GM12878 | ADPGK-AS1 | hsa-miR-484 | 31 | 52 | -32.06 | miRanda,TargetScan,RNAhybrid |
| GM12878 | SNHG14 | hsa-miR-574-5p | 136 | 158 | -27.25 | miRanda,TargetScan,RNAhybrid |
| GM12878 | SNHG14 | hsa-miR-574-5p | 19 | 41 | -27.25 | miRanda,TargetScan,RNAhybrid |
| GM12878 | MIR222HG | hsa-miR-574-5p | 290 | 312 | -31.92 | miRanda,TargetScan,RNAhybrid |
| GM12878 | USP3-AS1 | hsa-miR-612 | 56 | 82 | -32.46 | miRanda,TargetScan,RNAhybrid |
| GM12878 | LINC00298 | hsa-miR-632 | 72 | 90 | -22.47 | miRanda,TargetScan,RNAhybrid |
| GM12878 | LINC00298 | hsa-miR-632 | 74 | 92 | -22.47 | miRanda,TargetScan,RNAhybrid |
| GM12878 | MIR646HG | hsa-miR-650 | 232 | 252 | -28.7 | miRanda,TargetScan,RNAhybrid |
| GM12878 | LINC01126 | hsa-miR-663a | 459 | 481 | -37.78 | miRanda,TargetScan,RNAhybrid |
| GM12878 | LAMTOR5-AS1 | hsa-miR-761 | 242 | 263 | -32.26 | miRanda,TargetScan,RNAhybrid |
| GM12878 | A1BG-AS1 | hsa-miR-1204 | 67 | 89 | -32.56 | miRanda,TargetScan,RNAhybrid |
| GM12878 | A1BG-AS1 | hsa-miR-1204 | 81 | 101 | -23.54 | miRanda,TargetScan,RNAhybrid |
| GM12878 | SNHG14 | hsa-miR-3176 | 313 | 331 | -32.49 | miRanda,TargetScan,RNAhybrid |
| GM12878 | SNHG14 | hsa-miR-3176 | 300 | 318 | -32.49 | miRanda,TargetScan,RNAhybrid |
| GM12878 | SNHG14 | hsa-miR-3176 | 30 | 48 | -26.12 | miRanda,TargetScan,RNAhybrid |
| GM12878 | USP3-AS1 | hsa-miR-3176 | 44 | 62 | -25.83 | miRanda,TargetScan,RNAhybrid |
| GM12878 | SNHG12 | hsa-miR-3178 | 297 | 313 | -31.38 | miRanda,TargetScan,RNAhybrid |
| GM12878 | SNHG12 | hsa-miR-3178 | 441 | 457 | -31.38 | miRanda,TargetScan,RNAhybrid |
| GM12878 | SNHG12 | hsa-miR-3178 | 544 | 560 | -31.38 | miRanda,TargetScan,RNAhybrid |
| GM12878 | SNHG12 | hsa-miR-3178 | 612 | 628 | -31.38 | miRanda,TargetScan,RNAhybrid |
| GM12878 | SNHG12 | hsa-miR-3178 | 724 | 740 | -31.38 | miRanda,TargetScan,RNAhybrid |
| GM12878 | SNHG12 | hsa-miR-3178 | 932 | 948 | -31.38 | miRanda,TargetScan,RNAhybrid |
| GM12878 | SNHG12 | hsa-miR-3178 | 1255 | 1271 | -31.38 | miRanda,TargetScan,RNAhybrid |
| GM12878 | SNHG12 | hsa-miR-3178 | 213 | 229 | -31.38 | miRanda,TargetScan,RNAhybrid |
| GM12878 | SNHG12 | hsa-miR-3178 | 47 | 63 | -31.38 | miRanda,TargetScan,RNAhybrid |
| GM12878 | SNHG12 | hsa-miR-3178 | 186 | 202 | -31.38 | miRanda,TargetScan,RNAhybrid |
| GM12878 | SNHG12 | hsa-miR-3178 | 194 | 210 | -31.38 | miRanda,TargetScan,RNAhybrid |
| GM12878 | SNHG12 | hsa-miR-3178 | 205 | 221 | -31.38 | miRanda,TargetScan,RNAhybrid |
| GM12878 | SNHG12 | hsa-miR-3178 | 206 | 222 | -31.38 | miRanda,TargetScan,RNAhybrid |
| GM12878 | SNHG12 | hsa-miR-3178 | 209 | 225 | -31.38 | miRanda,TargetScan,RNAhybrid |
| GM12878 | SNHG12 | hsa-miR-3178 | 224 | 240 | -31.38 | miRanda,TargetScan,RNAhybrid |
| GM12878 | SNHG12 | hsa-miR-3178 | 225 | 241 | -31.38 | miRanda,TargetScan,RNAhybrid |
| GM12878 | SNHG12 | hsa-miR-3178 | 230 | 246 | -31.38 | miRanda,TargetScan,RNAhybrid |
| GM12878 | SNHG12 | hsa-miR-3178 | 15 | 31 | -31.38 | miRanda,TargetScan,RNAhybrid |
| GM12878 | SNHG12 | hsa-miR-3178 | 170 | 186 | -31.38 | miRanda,TargetScan,RNAhybrid |
| GM12878 | SNHG12 | hsa-miR-3178 | 149 | 165 | -31.38 | miRanda,TargetScan,RNAhybrid |
| GM12878 | SNHG12 | hsa-miR-3178 | 219 | 235 | -31.38 | miRanda,TargetScan,RNAhybrid |
| GM12878 | SNHG12 | hsa-miR-3178 | 223 | 239 | -31.38 | miRanda,TargetScan,RNAhybrid |
| GM12878 | CAHM | hsa-miR-3178 | 35 | 53 | -32.32 | miRanda,TargetScan,RNAhybrid |
| GM12878 | MSC-AS1 | hsa-miR-3178 | 64 | 81 | -30.27 | miRanda,TargetScan,RNAhybrid |
| GM12878 | ZEB1-AS1 | hsa-miR-3178 | 260 | 276 | -31.96 | miRanda,TargetScan,RNAhybrid |
| GM12878 | LINC00605 | hsa-miR-3178 | 102 | 118 | -33.1 | miRanda,TargetScan,RNAhybrid |
| GM12878 | LINC00605 | hsa-miR-3178 | 159 | 175 | -33.1 | miRanda,TargetScan,RNAhybrid |
| GM12878 | MMP25-AS1 | hsa-miR-3178 | 867 | 883 | -36.22 | miRanda,TargetScan,RNAhybrid |
| GM12878 | MMP25-AS1 | hsa-miR-3178 | 840 | 856 | -36.22 | miRanda,TargetScan,RNAhybrid |
| GM12878 | LINC00910 | hsa-miR-3178 | 517 | 533 | -28.76 | miRanda,TargetScan,RNAhybrid |
| GM12878 | LINC00910 | hsa-miR-3178 | 542 | 558 | -28.76 | miRanda,TargetScan,RNAhybrid |
| GM12878 | LINC00910 | hsa-miR-3178 | 857 | 873 | -28.76 | miRanda,TargetScan,RNAhybrid |
| GM12878 | LINC00910 | hsa-miR-3178 | 648 | 664 | -28.76 | miRanda,TargetScan,RNAhybrid |
| GM12878 | LINC00910 | hsa-miR-3178 | 577 | 593 | -28.76 | miRanda,TargetScan,RNAhybrid |
| GM12878 | LINC00910 | hsa-miR-3178 | 506 | 522 | -28.76 | miRanda,TargetScan,RNAhybrid |
| GM12878 | LINC00910 | hsa-miR-3178 | 529 | 545 | -28.76 | miRanda,TargetScan,RNAhybrid |
| GM12878 | LINC00910 | hsa-miR-3178 | 257 | 273 | -28.76 | miRanda,TargetScan,RNAhybrid |
| GM12878 | ASB16-AS1 | hsa-miR-3178 | 851 | 867 | -32.15 | miRanda,TargetScan,RNAhybrid |
| GM12878 | PAX8-AS1 | hsa-miR-320e | 15 | 32 | -25.81 | miRanda,TargetScan,RNAhybrid |
| GM12878 | NR2F2-AS1 | hsa-miR-3195 | 76 | 92 | -28.24 | miRanda,TargetScan,RNAhybrid |
| GM12878 | NR2F2-AS1 | hsa-miR-3195 | 26 | 42 | -28.24 | miRanda,TargetScan,RNAhybrid |
| GM12878 | NR2F2-AS1 | hsa-miR-3195 | 74 | 90 | -28.24 | miRanda,TargetScan,RNAhybrid |
| GM12878 | NR2F2-AS1 | hsa-miR-3195 | 55 | 71 | -28.24 | miRanda,TargetScan,RNAhybrid |
| GM12878 | NR2F2-AS1 | hsa-miR-3195 | 87 | 103 | -28.24 | miRanda,TargetScan,RNAhybrid |
| GM12878 | SNAI3-AS1 | hsa-miR-3195 | 75 | 91 | -31.24 | miRanda,TargetScan,RNAhybrid |
| GM12878 | LINC00894 | hsa-miR-3195 | 908 | 924 | -33.3 | miRanda,TargetScan,RNAhybrid |
| GM12878 | MMP25-AS1 | hsa-miR-4286 | 190 | 206 | -26.51 | miRanda,TargetScan,RNAhybrid |
| GM12878 | LINC00299 | hsa-miR-326 | 54 | 73 | -32.05 | miRanda,TargetScan,RNAhybrid |
| GM12878 | LINC00299 | hsa-miR-331-3p | 199 | 219 | -29.5 | miRanda,TargetScan,RNAhybrid |
| GM12878 | LINC01239 | hsa-miR-331-3p | 603 | 623 | -29.48 | miRanda,TargetScan,RNAhybrid |
| GM12878 | LINC01239 | hsa-miR-331-3p | 184 | 204 | -29.48 | miRanda,TargetScan,RNAhybrid |
| GM12878 | LINC01239 | hsa-miR-331-3p | 126 | 146 | -29.48 | miRanda,TargetScan,RNAhybrid |
| GM12878 | PWAR5 | hsa-miR-574-5p | 136 | 158 | -27.25 | miRanda,TargetScan,RNAhybrid |
| GM12878 | PWAR5 | hsa-miR-574-5p | 19 | 41 | -27.25 | miRanda,TargetScan,RNAhybrid |
| GM12878 | FAM66B | hsa-miR-612 | 146 | 170 | -33.24 | miRanda,TargetScan,RNAhybrid |
| GM12878 | LINC00299 | hsa-miR-632 | 72 | 90 | -22.47 | miRanda,TargetScan,RNAhybrid |
| GM12878 | LINC00299 | hsa-miR-632 | 74 | 92 | -22.47 | miRanda,TargetScan,RNAhybrid |
| GM12878 | PWAR5 | hsa-miR-3176 | 313 | 331 | -32.49 | miRanda,TargetScan,RNAhybrid |
| GM12878 | PWAR5 | hsa-miR-3176 | 300 | 318 | -32.49 | miRanda,TargetScan,RNAhybrid |
| GM12878 | PWAR5 | hsa-miR-3176 | 30 | 48 | -26.12 | miRanda,TargetScan,RNAhybrid |
| H1.hESC | LIPE-AS1 | hsa-miR-198 | 1255 | 1276 | -36.21 | miRanda,TargetScan,RNAhybrid |
| H1.hESC | LINC01600 | hsa-miR-326 | 685 | 704 | -34.52 | miRanda,TargetScan,RNAhybrid |
| H1.hESC | LINC01600 | hsa-miR-326 | 622 | 641 | -34.52 | miRanda,TargetScan,RNAhybrid |
| H1.hESC | KRTAP5-AS1 | hsa-miR-326 | 532 | 551 | -37.75 | miRanda,TargetScan,RNAhybrid |
| H1.hESC | KRTAP5-AS1 | hsa-miR-326 | 391 | 410 | -37.75 | miRanda,TargetScan,RNAhybrid |
| H1.hESC | MRGPRG-AS1 | hsa-miR-326 | 615 | 634 | -29.31 | miRanda,TargetScan,RNAhybrid |
| H1.hESC | MRGPRG-AS1 | hsa-miR-326 | 676 | 695 | -29.31 | miRanda,TargetScan,RNAhybrid |
| H1.hESC | LINC00668 | hsa-miR-326 | 28 | 48 | -32.78 | miRanda,TargetScan,RNAhybrid |
| H1.hESC | LINC01270 | hsa-miR-326 | 60 | 79 | -34.19 | miRanda,TargetScan,RNAhybrid |
| H1.hESC | LINC01270 | hsa-miR-326 | 49 | 68 | -34.19 | miRanda,TargetScan,RNAhybrid |
| H1.hESC | LINC01270 | hsa-miR-326 | 20 | 39 | -34.19 | miRanda,TargetScan,RNAhybrid |
| H1.hESC | MIR4435-2HG | hsa-miR-331-3p | 786 | 806 | -29.98 | miRanda,TargetScan,RNAhybrid |
| H1.hESC | FAM85B | hsa-miR-331-3p | 59 | 79 | -33.94 | miRanda,TargetScan,RNAhybrid |
| H1.hESC | FAM85B | hsa-miR-331-3p | 10 | 30 | -33.94 | miRanda,TargetScan,RNAhybrid |
| H1.hESC | CASC2 | hsa-miR-331-3p | 42 | 61 | -30.94 | miRanda,TargetScan,RNAhybrid |
| H1.hESC | MRGPRG-AS1 | hsa-miR-331-3p | 388 | 407 | -29.54 | miRanda,TargetScan,RNAhybrid |
| H1.hESC | MRGPRG-AS1 | hsa-miR-331-3p | 494 | 513 | -29.54 | miRanda,TargetScan,RNAhybrid |
| H1.hESC | MRGPRG-AS1 | hsa-miR-331-3p | 538 | 557 | -29.54 | miRanda,TargetScan,RNAhybrid |
| H1.hESC | MRGPRG-AS1 | hsa-miR-331-3p | 449 | 468 | -29.54 | miRanda,TargetScan,RNAhybrid |
| H1.hESC | MRGPRG-AS1 | hsa-miR-331-3p | 527 | 546 | -29.54 | miRanda,TargetScan,RNAhybrid |
| H1.hESC | MRGPRF-AS1 | hsa-miR-331-3p | 564 | 584 | -28.13 | miRanda,TargetScan,RNAhybrid |
| H1.hESC | MRGPRF-AS1 | hsa-miR-331-3p | 138 | 158 | -28.13 | miRanda,TargetScan,RNAhybrid |
| H1.hESC | USP2-AS1 | hsa-miR-331-3p | 26 | 46 | -33.27 | miRanda,TargetScan,RNAhybrid |
| H1.hESC | MEG3 | hsa-miR-331-3p | 235 | 255 | -31.44 | miRanda,TargetScan,RNAhybrid |
| H1.hESC | DIO3OS | hsa-miR-331-3p | 188 | 208 | -28.62 | miRanda,TargetScan,RNAhybrid |
| H1.hESC | DIO3OS | hsa-miR-331-3p | 274 | 294 | -28.62 | miRanda,TargetScan,RNAhybrid |
| H1.hESC | FAM157C | hsa-miR-331-3p | 1169 | 1189 | -30.43 | miRanda,TargetScan,RNAhybrid |
| H1.hESC | FAM157C | hsa-miR-331-3p | 1006 | 1026 | -30.43 | miRanda,TargetScan,RNAhybrid |
| H1.hESC | FAM157C | hsa-miR-331-3p | 1111 | 1131 | -30.43 | miRanda,TargetScan,RNAhybrid |
| H1.hESC | FAM157C | hsa-miR-331-3p | 490 | 510 | -30.43 | miRanda,TargetScan,RNAhybrid |
| H1.hESC | FAM157C | hsa-miR-331-3p | 21 | 41 | -30.43 | miRanda,TargetScan,RNAhybrid |
| H1.hESC | LINC00511 | hsa-miR-331-3p | 201 | 220 | -27.07 | miRanda,TargetScan,RNAhybrid |
| H1.hESC | LINC00511 | hsa-miR-331-3p | 334 | 353 | -27.07 | miRanda,TargetScan,RNAhybrid |
| H1.hESC | LINC00511 | hsa-miR-331-3p | 122 | 141 | -27.07 | miRanda,TargetScan,RNAhybrid |
| H1.hESC | LINC00511 | hsa-miR-331-3p | 473 | 492 | -27.07 | miRanda,TargetScan,RNAhybrid |
| H1.hESC | LINC00511 | hsa-miR-331-3p | 595 | 614 | -27.07 | miRanda,TargetScan,RNAhybrid |
| H1.hESC | NARF-IT1 | hsa-miR-331-3p | 281 | 301 | -36.15 | miRanda,TargetScan,RNAhybrid |
| H1.hESC | LINC00310 | hsa-miR-331-3p | 429 | 449 | -30.57 | miRanda,TargetScan,RNAhybrid |
| H1.hESC | LINC00310 | hsa-miR-331-3p | 808 | 828 | -30.57 | miRanda,TargetScan,RNAhybrid |
| H1.hESC | LINC00310 | hsa-miR-331-3p | 788 | 808 | -30.57 | miRanda,TargetScan,RNAhybrid |
| H1.hESC | LINC00310 | hsa-miR-331-3p | 792 | 812 | -30.57 | miRanda,TargetScan,RNAhybrid |
| H1.hESC | ADPGK-AS1 | hsa-miR-484 | 31 | 52 | -32.06 | miRanda,TargetScan,RNAhybrid |
| H1.hESC | FEZF1-AS1 | hsa-miR-492 | 1360 | 1382 | -30.97 | miRanda,TargetScan,RNAhybrid |
| H1.hESC | SNHG14 | hsa-miR-574-5p | 136 | 158 | -27.25 | miRanda,TargetScan,RNAhybrid |
| H1.hESC | SNHG14 | hsa-miR-574-5p | 19 | 41 | -27.25 | miRanda,TargetScan,RNAhybrid |
| H1.hESC | MIR222HG | hsa-miR-574-5p | 290 | 312 | -31.92 | miRanda,TargetScan,RNAhybrid |
| H1.hESC | LINC00853 | hsa-miR-612 | 825 | 849 | -33.84 | miRanda,TargetScan,RNAhybrid |
| H1.hESC | LINC00853 | hsa-miR-612 | 833 | 857 | -33.84 | miRanda,TargetScan,RNAhybrid |
| H1.hESC | LINC00853 | hsa-miR-612 | 575 | 599 | -33.84 | miRanda,TargetScan,RNAhybrid |
| H1.hESC | LINC00853 | hsa-miR-612 | 786 | 810 | -33.84 | miRanda,TargetScan,RNAhybrid |
| H1.hESC | LINC00853 | hsa-miR-612 | 709 | 733 | -33.84 | miRanda,TargetScan,RNAhybrid |
| H1.hESC | LINC00853 | hsa-miR-612 | 481 | 505 | -33.84 | miRanda,TargetScan,RNAhybrid |
| H1.hESC | LINC00853 | hsa-miR-612 | 910 | 934 | -33.84 | miRanda,TargetScan,RNAhybrid |
| H1.hESC | LINGO1-AS1 | hsa-miR-612 | 65 | 89 | -45.01 | miRanda,TargetScan,RNAhybrid |
| H1.hESC | PTOV1-AS2 | hsa-miR-612 | 205 | 234 | -37.47 | miRanda,TargetScan,RNAhybrid |
| H1.hESC | PTOV1-AS2 | hsa-miR-612 | 216 | 245 | -37.47 | miRanda,TargetScan,RNAhybrid |
| H1.hESC | SEMA3F-AS1 | hsa-miR-650 | 102 | 122 | -27.92 | miRanda,TargetScan,RNAhybrid |
| H1.hESC | LINC01126 | hsa-miR-663a | 459 | 481 | -37.78 | miRanda,TargetScan,RNAhybrid |
| H1.hESC | LAMTOR5-AS1 | hsa-miR-761 | 242 | 263 | -32.26 | miRanda,TargetScan,RNAhybrid |
| H1.hESC | LINC01515 | hsa-miR-761 | 370 | 392 | -23.56 | miRanda,TargetScan,RNAhybrid |
| H1.hESC | LINC01122 | hsa-miR-892b | 811 | 832 | -34.95 | miRanda,TargetScan,RNAhybrid |
| H1.hESC | LINC01122 | hsa-miR-892b | 651 | 672 | -34.95 | miRanda,TargetScan,RNAhybrid |
| H1.hESC | LINC01122 | hsa-miR-892b | 798 | 819 | -34.95 | miRanda,TargetScan,RNAhybrid |
| H1.hESC | LINC01122 | hsa-miR-892b | 542 | 563 | -34.95 | miRanda,TargetScan,RNAhybrid |
| H1.hESC | LINC01122 | hsa-miR-892b | 645 | 666 | -34.95 | miRanda,TargetScan,RNAhybrid |
| H1.hESC | LINC01122 | hsa-miR-892b | 695 | 716 | -34.95 | miRanda,TargetScan,RNAhybrid |
| H1.hESC | LINC01122 | hsa-miR-892b | 958 | 979 | -34.95 | miRanda,TargetScan,RNAhybrid |
| H1.hESC | LINC01122 | hsa-miR-892b | 577 | 598 | -34.95 | miRanda,TargetScan,RNAhybrid |
| H1.hESC | LINC01122 | hsa-miR-892b | 686 | 707 | -34.95 | miRanda,TargetScan,RNAhybrid |
| H1.hESC | LINC01122 | hsa-miR-892b | 733 | 754 | -34.95 | miRanda,TargetScan,RNAhybrid |
| H1.hESC | LINC01122 | hsa-miR-892b | 673 | 694 | -34.95 | miRanda,TargetScan,RNAhybrid |
| H1.hESC | LINC01122 | hsa-miR-892b | 556 | 577 | -34.95 | miRanda,TargetScan,RNAhybrid |
| H1.hESC | LINC01122 | hsa-miR-892b | 205 | 226 | -34.95 | miRanda,TargetScan,RNAhybrid |
| H1.hESC | A1BG-AS1 | hsa-miR-1204 | 67 | 89 | -32.56 | miRanda,TargetScan,RNAhybrid |
| H1.hESC | A1BG-AS1 | hsa-miR-1204 | 81 | 101 | -23.54 | miRanda,TargetScan,RNAhybrid |
| H1.hESC | LINC01118 | hsa-miR-1303 | 77 | 98 | -37.75 | miRanda,TargetScan,RNAhybrid |
| H1.hESC | LINC01118 | hsa-miR-1303 | 60 | 81 | -37.75 | miRanda,TargetScan,RNAhybrid |
| H1.hESC | GATA2-AS1 | hsa-miR-3176 | 61 | 79 | -27.53 | miRanda,TargetScan,RNAhybrid |
| H1.hESC | GATA2-AS1 | hsa-miR-3176 | 48 | 66 | -27.53 | miRanda,TargetScan,RNAhybrid |
| H1.hESC | SNHG14 | hsa-miR-3176 | 313 | 331 | -32.49 | miRanda,TargetScan,RNAhybrid |
| H1.hESC | SNHG14 | hsa-miR-3176 | 300 | 318 | -32.49 | miRanda,TargetScan,RNAhybrid |
| H1.hESC | SNHG14 | hsa-miR-3176 | 30 | 48 | -26.12 | miRanda,TargetScan,RNAhybrid |
| H1.hESC | SNHG12 | hsa-miR-3178 | 297 | 313 | -31.38 | miRanda,TargetScan,RNAhybrid |
| H1.hESC | SNHG12 | hsa-miR-3178 | 441 | 457 | -31.38 | miRanda,TargetScan,RNAhybrid |
| H1.hESC | SNHG12 | hsa-miR-3178 | 544 | 560 | -31.38 | miRanda,TargetScan,RNAhybrid |
| H1.hESC | SNHG12 | hsa-miR-3178 | 612 | 628 | -31.38 | miRanda,TargetScan,RNAhybrid |
| H1.hESC | SNHG12 | hsa-miR-3178 | 724 | 740 | -31.38 | miRanda,TargetScan,RNAhybrid |
| H1.hESC | SNHG12 | hsa-miR-3178 | 932 | 948 | -31.38 | miRanda,TargetScan,RNAhybrid |
| H1.hESC | SNHG12 | hsa-miR-3178 | 1255 | 1271 | -31.38 | miRanda,TargetScan,RNAhybrid |
| H1.hESC | SNHG12 | hsa-miR-3178 | 213 | 229 | -31.38 | miRanda,TargetScan,RNAhybrid |
| H1.hESC | SNHG12 | hsa-miR-3178 | 47 | 63 | -31.38 | miRanda,TargetScan,RNAhybrid |
| H1.hESC | SNHG12 | hsa-miR-3178 | 186 | 202 | -31.38 | miRanda,TargetScan,RNAhybrid |
| H1.hESC | SNHG12 | hsa-miR-3178 | 194 | 210 | -31.38 | miRanda,TargetScan,RNAhybrid |
| H1.hESC | SNHG12 | hsa-miR-3178 | 205 | 221 | -31.38 | miRanda,TargetScan,RNAhybrid |
| H1.hESC | SNHG12 | hsa-miR-3178 | 206 | 222 | -31.38 | miRanda,TargetScan,RNAhybrid |
| H1.hESC | SNHG12 | hsa-miR-3178 | 209 | 225 | -31.38 | miRanda,TargetScan,RNAhybrid |
| H1.hESC | SNHG12 | hsa-miR-3178 | 224 | 240 | -31.38 | miRanda,TargetScan,RNAhybrid |
| H1.hESC | SNHG12 | hsa-miR-3178 | 225 | 241 | -31.38 | miRanda,TargetScan,RNAhybrid |
| H1.hESC | SNHG12 | hsa-miR-3178 | 230 | 246 | -31.38 | miRanda,TargetScan,RNAhybrid |
| H1.hESC | SNHG12 | hsa-miR-3178 | 15 | 31 | -31.38 | miRanda,TargetScan,RNAhybrid |
| H1.hESC | SNHG12 | hsa-miR-3178 | 170 | 186 | -31.38 | miRanda,TargetScan,RNAhybrid |
| H1.hESC | SNHG12 | hsa-miR-3178 | 149 | 165 | -31.38 | miRanda,TargetScan,RNAhybrid |
| H1.hESC | SNHG12 | hsa-miR-3178 | 219 | 235 | -31.38 | miRanda,TargetScan,RNAhybrid |
| H1.hESC | SNHG12 | hsa-miR-3178 | 223 | 239 | -31.38 | miRanda,TargetScan,RNAhybrid |
| H1.hESC | CAHM | hsa-miR-3178 | 35 | 53 | -32.32 | miRanda,TargetScan,RNAhybrid |
| H1.hESC | MSC-AS1 | hsa-miR-3178 | 64 | 81 | -30.27 | miRanda,TargetScan,RNAhybrid |
| H1.hESC | LINC00958 | hsa-miR-3178 | 12 | 28 | -28.52 | miRanda,TargetScan,RNAhybrid |
| H1.hESC | LINC00605 | hsa-miR-3178 | 102 | 118 | -33.1 | miRanda,TargetScan,RNAhybrid |
| H1.hESC | LINC00605 | hsa-miR-3178 | 159 | 175 | -33.1 | miRanda,TargetScan,RNAhybrid |
| H1.hESC | LINC00637 | hsa-miR-3178 | 456 | 472 | -32.07 | miRanda,TargetScan,RNAhybrid |
| H1.hESC | MMP25-AS1 | hsa-miR-3178 | 867 | 883 | -36.22 | miRanda,TargetScan,RNAhybrid |
| H1.hESC | MMP25-AS1 | hsa-miR-3178 | 840 | 856 | -36.22 | miRanda,TargetScan,RNAhybrid |
| H1.hESC | LINC00910 | hsa-miR-3178 | 517 | 533 | -28.76 | miRanda,TargetScan,RNAhybrid |
| H1.hESC | LINC00910 | hsa-miR-3178 | 542 | 558 | -28.76 | miRanda,TargetScan,RNAhybrid |
| H1.hESC | LINC00910 | hsa-miR-3178 | 857 | 873 | -28.76 | miRanda,TargetScan,RNAhybrid |
| H1.hESC | LINC00910 | hsa-miR-3178 | 648 | 664 | -28.76 | miRanda,TargetScan,RNAhybrid |
| H1.hESC | LINC00910 | hsa-miR-3178 | 577 | 593 | -28.76 | miRanda,TargetScan,RNAhybrid |
| H1.hESC | LINC00910 | hsa-miR-3178 | 506 | 522 | -28.76 | miRanda,TargetScan,RNAhybrid |
| H1.hESC | LINC00910 | hsa-miR-3178 | 529 | 545 | -28.76 | miRanda,TargetScan,RNAhybrid |
| H1.hESC | LINC00910 | hsa-miR-3178 | 257 | 273 | -28.76 | miRanda,TargetScan,RNAhybrid |
| H1.hESC | ASB16-AS1 | hsa-miR-3178 | 851 | 867 | -32.15 | miRanda,TargetScan,RNAhybrid |
| H1.hESC | LINC00899 | hsa-miR-3178 | 25 | 41 | -36.65 | miRanda,TargetScan,RNAhybrid |
| H1.hESC | LINC00899 | hsa-miR-3178 | 10 | 26 | -36.65 | miRanda,TargetScan,RNAhybrid |
| H1.hESC | LINC00899 | hsa-miR-3178 | 88 | 104 | -36.65 | miRanda,TargetScan,RNAhybrid |
| H1.hESC | LINC00899 | hsa-miR-3178 | 53 | 69 | -36.65 | miRanda,TargetScan,RNAhybrid |
| H1.hESC | HAS2-AS1 | hsa-miR-3182 | 113 | 129 | -22.1 | miRanda,TargetScan,RNAhybrid |
| H1.hESC | HAS2-AS1 | hsa-miR-3182 | 111 | 127 | -22.1 | miRanda,TargetScan,RNAhybrid |
| H1.hESC | PXN-AS1 | hsa-miR-3183 | 1013 | 1034 | -30.77 | miRanda,TargetScan,RNAhybrid |
| H1.hESC | PXN-AS1 | hsa-miR-3183 | 1127 | 1148 | -30.77 | miRanda,TargetScan,RNAhybrid |
| H1.hESC | PXN-AS1 | hsa-miR-3183 | 1110 | 1131 | -30.77 | miRanda,TargetScan,RNAhybrid |
| H1.hESC | PXN-AS1 | hsa-miR-3183 | 1014 | 1035 | -30.77 | miRanda,TargetScan,RNAhybrid |
| H1.hESC | LINC00894 | hsa-miR-3195 | 908 | 924 | -33.3 | miRanda,TargetScan,RNAhybrid |
| H1.hESC | DNAH17-AS1 | hsa-miR-3197 | 182 | 204 | -33.96 | miRanda,TargetScan,RNAhybrid |
| H1.hESC | DNAH17-AS1 | hsa-miR-3197 | 169 | 191 | -33.96 | miRanda,TargetScan,RNAhybrid |
| H1.hESC | DNAH17-AS1 | hsa-miR-3197 | 140 | 162 | -33.96 | miRanda,TargetScan,RNAhybrid |
| H1.hESC | LINC01535 | hsa-miR-3197 | 484 | 506 | -33.16 | miRanda,TargetScan,RNAhybrid |
| H1.hESC | TMEM72-AS1 | hsa-miR-3198 | 926 | 947 | -34.32 | miRanda,TargetScan,RNAhybrid |
| H1.hESC | DIO3OS | hsa-miR-3198 | 71 | 92 | -37.9 | miRanda,TargetScan,RNAhybrid |
| H1.hESC | DIO3OS | hsa-miR-3198 | 152 | 173 | -37.9 | miRanda,TargetScan,RNAhybrid |
| H1.hESC | DIO3OS | hsa-miR-3198 | 270 | 291 | -37.9 | miRanda,TargetScan,RNAhybrid |
| H1.hESC | DIO3OS | hsa-miR-3198 | 281 | 302 | -37.9 | miRanda,TargetScan,RNAhybrid |
| H1.hESC | DIO3OS | hsa-miR-3198 | 636 | 657 | -37.9 | miRanda,TargetScan,RNAhybrid |
| H1.hESC | DIO3OS | hsa-miR-3198 | 722 | 743 | -37.9 | miRanda,TargetScan,RNAhybrid |
| H1.hESC | POT1-AS1 | hsa-miR-4286 | 897 | 913 | -26.71 | miRanda,TargetScan,RNAhybrid |
| H1.hESC | MMP25-AS1 | hsa-miR-4286 | 190 | 206 | -26.51 | miRanda,TargetScan,RNAhybrid |
| H1.hESC | LINC00511 | hsa-miR-4286 | 282 | 298 | -32.18 | miRanda,TargetScan,RNAhybrid |
| H1.hESC | LINC00511 | hsa-miR-4286 | 679 | 695 | -32.18 | miRanda,TargetScan,RNAhybrid |
| H1.hESC | FAM230B | hsa-miR-4286 | 431 | 447 | -27.62 | miRanda,TargetScan,RNAhybrid |
| H1.hESC | LINC00299 | hsa-miR-326 | 54 | 73 | -32.05 | miRanda,TargetScan,RNAhybrid |
| H1.hESC | LINC00299 | hsa-miR-331-3p | 199 | 219 | -29.5 | miRanda,TargetScan,RNAhybrid |
| H1.hESC | LINC01239 | hsa-miR-331-3p | 603 | 623 | -29.48 | miRanda,TargetScan,RNAhybrid |
| H1.hESC | LINC01239 | hsa-miR-331-3p | 184 | 204 | -29.48 | miRanda,TargetScan,RNAhybrid |
| H1.hESC | LINC01239 | hsa-miR-331-3p | 126 | 146 | -29.48 | miRanda,TargetScan,RNAhybrid |
| H1.hESC | PWAR5 | hsa-miR-574-5p | 136 | 158 | -27.25 | miRanda,TargetScan,RNAhybrid |
| H1.hESC | PWAR5 | hsa-miR-574-5p | 19 | 41 | -27.25 | miRanda,TargetScan,RNAhybrid |
| H1.hESC | FAM66B | hsa-miR-612 | 146 | 170 | -33.24 | miRanda,TargetScan,RNAhybrid |
| H1.hESC | LINC00299 | hsa-miR-632 | 72 | 90 | -22.47 | miRanda,TargetScan,RNAhybrid |
| H1.hESC | LINC00299 | hsa-miR-632 | 74 | 92 | -22.47 | miRanda,TargetScan,RNAhybrid |
| H1.hESC | LINC01119 | hsa-miR-1303 | 77 | 98 | -37.75 | miRanda,TargetScan,RNAhybrid |
| H1.hESC | LINC01119 | hsa-miR-1303 | 60 | 81 | -37.75 | miRanda,TargetScan,RNAhybrid |
| H1.hESC | PWAR5 | hsa-miR-3176 | 313 | 331 | -32.49 | miRanda,TargetScan,RNAhybrid |
| H1.hESC | PWAR5 | hsa-miR-3176 | 300 | 318 | -32.49 | miRanda,TargetScan,RNAhybrid |
| H1.hESC | PWAR5 | hsa-miR-3176 | 30 | 48 | -26.12 | miRanda,TargetScan,RNAhybrid |
| H1.hESC | DLEU7-AS1 | hsa-miR-4286 | 380 | 396 | -32.51 | miRanda,TargetScan,RNAhybrid |
| H1.hESC | DLEU7-AS1 | hsa-miR-4286 | 527 | 543 | -27.5 | miRanda,TargetScan,RNAhybrid |
| H1.hESC | DLEU7-AS1 | hsa-miR-4286 | 420 | 436 | -27.5 | miRanda,TargetScan,RNAhybrid |
| H1.hESC | DLEU7-AS1 | hsa-miR-4286 | 278 | 294 | -27.5 | miRanda,TargetScan,RNAhybrid |
| H1.hESC | DLEU7-AS1 | hsa-miR-4286 | 199 | 215 | -27.5 | miRanda,TargetScan,RNAhybrid |
| H1.hESC | DLEU7-AS1 | hsa-miR-4286 | 87 | 103 | -27.5 | miRanda,TargetScan,RNAhybrid |
| H1.hESC | DLEU7-AS1 | hsa-miR-4286 | 85 | 101 | -27.5 | miRanda,TargetScan,RNAhybrid |
| H1.hESC | DLEU7-AS1 | hsa-miR-4286 | 38 | 54 | -27.5 | miRanda,TargetScan,RNAhybrid |
| H1.hESC | DLEU7-AS1 | hsa-miR-4286 | 359 | 375 | -30.84 | miRanda,TargetScan,RNAhybrid |
| H1.hESC | PWAR1 | hsa-miR-574-5p | 136 | 158 | -27.25 | miRanda,TargetScan,RNAhybrid |
| H1.hESC | PWAR1 | hsa-miR-574-5p | 19 | 41 | -27.25 | miRanda,TargetScan,RNAhybrid |
| H1.hESC | PWAR1 | hsa-miR-3176 | 313 | 331 | -32.49 | miRanda,TargetScan,RNAhybrid |
| H1.hESC | PWAR1 | hsa-miR-3176 | 300 | 318 | -32.49 | miRanda,TargetScan,RNAhybrid |
| H1.hESC | PWAR1 | hsa-miR-3176 | 30 | 48 | -26.12 | miRanda,TargetScan,RNAhybrid |
| HeLa.S3 | LINC01600 | hsa-miR-326 | 685 | 704 | -34.52 | miRanda,TargetScan,RNAhybrid |
| HeLa.S3 | LINC01600 | hsa-miR-326 | 622 | 641 | -34.52 | miRanda,TargetScan,RNAhybrid |
| HeLa.S3 | CDKN2B-AS1 | hsa-miR-331-3p | 603 | 623 | -29.48 | miRanda,TargetScan,RNAhybrid |
| HeLa.S3 | CDKN2B-AS1 | hsa-miR-331-3p | 184 | 204 | -29.48 | miRanda,TargetScan,RNAhybrid |
| HeLa.S3 | CDKN2B-AS1 | hsa-miR-331-3p | 126 | 146 | -29.48 | miRanda,TargetScan,RNAhybrid |
| HeLa.S3 | DIO3OS | hsa-miR-331-3p | 188 | 208 | -28.62 | miRanda,TargetScan,RNAhybrid |
| HeLa.S3 | DIO3OS | hsa-miR-331-3p | 274 | 294 | -28.62 | miRanda,TargetScan,RNAhybrid |
| HeLa.S3 | FAM157C | hsa-miR-331-3p | 1169 | 1189 | -30.43 | miRanda,TargetScan,RNAhybrid |
| HeLa.S3 | FAM157C | hsa-miR-331-3p | 1006 | 1026 | -30.43 | miRanda,TargetScan,RNAhybrid |
| HeLa.S3 | FAM157C | hsa-miR-331-3p | 1111 | 1131 | -30.43 | miRanda,TargetScan,RNAhybrid |
| HeLa.S3 | FAM157C | hsa-miR-331-3p | 490 | 510 | -30.43 | miRanda,TargetScan,RNAhybrid |
| HeLa.S3 | FAM157C | hsa-miR-331-3p | 21 | 41 | -30.43 | miRanda,TargetScan,RNAhybrid |
| HeLa.S3 | ADPGK-AS1 | hsa-miR-484 | 31 | 52 | -32.06 | miRanda,TargetScan,RNAhybrid |
| HeLa.S3 | PTOV1-AS2 | hsa-miR-612 | 205 | 234 | -37.47 | miRanda,TargetScan,RNAhybrid |
| HeLa.S3 | PTOV1-AS2 | hsa-miR-612 | 216 | 245 | -37.47 | miRanda,TargetScan,RNAhybrid |
| HeLa.S3 | LINC01126 | hsa-miR-663a | 459 | 481 | -37.78 | miRanda,TargetScan,RNAhybrid |
| HeLa.S3 | LAMTOR5-AS1 | hsa-miR-761 | 242 | 263 | -32.26 | miRanda,TargetScan,RNAhybrid |
| HeLa.S3 | SEMA3B-AS1 | hsa-miR-760 | 112 | 132 | -38.68 | miRanda,TargetScan,RNAhybrid |
| HeLa.S3 | CAHM | hsa-miR-3178 | 35 | 53 | -32.32 | miRanda,TargetScan,RNAhybrid |
| HeLa.S3 | ZEB1-AS1 | hsa-miR-3178 | 260 | 276 | -31.96 | miRanda,TargetScan,RNAhybrid |
| HeLa.S3 | MMP25-AS1 | hsa-miR-3178 | 867 | 883 | -36.22 | miRanda,TargetScan,RNAhybrid |
| HeLa.S3 | MMP25-AS1 | hsa-miR-3178 | 840 | 856 | -36.22 | miRanda,TargetScan,RNAhybrid |
| HeLa.S3 | ASB16-AS1 | hsa-miR-3178 | 851 | 867 | -32.15 | miRanda,TargetScan,RNAhybrid |
| HeLa.S3 | PAX8-AS1 | hsa-miR-320e | 15 | 32 | -25.81 | miRanda,TargetScan,RNAhybrid |
| HeLa.S3 | ZMIZ1-AS1 | hsa-miR-3195 | 601 | 617 | -35.09 | miRanda,TargetScan,RNAhybrid |
| HeLa.S3 | DIO3OS | hsa-miR-3198 | 71 | 92 | -37.9 | miRanda,TargetScan,RNAhybrid |
| HeLa.S3 | DIO3OS | hsa-miR-3198 | 152 | 173 | -37.9 | miRanda,TargetScan,RNAhybrid |
| HeLa.S3 | DIO3OS | hsa-miR-3198 | 270 | 291 | -37.9 | miRanda,TargetScan,RNAhybrid |
| HeLa.S3 | DIO3OS | hsa-miR-3198 | 281 | 302 | -37.9 | miRanda,TargetScan,RNAhybrid |
| HeLa.S3 | DIO3OS | hsa-miR-3198 | 636 | 657 | -37.9 | miRanda,TargetScan,RNAhybrid |
| HeLa.S3 | DIO3OS | hsa-miR-3198 | 722 | 743 | -37.9 | miRanda,TargetScan,RNAhybrid |
| HeLa.S3 | POT1-AS1 | hsa-miR-4286 | 897 | 913 | -26.71 | miRanda,TargetScan,RNAhybrid |
| HeLa.S3 | HOTAIR | hsa-miR-4286 | 275 | 291 | -22.5 | miRanda,TargetScan,RNAhybrid |
| HeLa.S3 | MMP25-AS1 | hsa-miR-4286 | 190 | 206 | -26.51 | miRanda,TargetScan,RNAhybrid |
| HepG2 | LIPE-AS1 | hsa-miR-198 | 1255 | 1276 | -36.21 | miRanda,TargetScan,RNAhybrid |
| HepG2 | LINC01600 | hsa-miR-326 | 685 | 704 | -34.52 | miRanda,TargetScan,RNAhybrid |
| HepG2 | LINC01600 | hsa-miR-326 | 622 | 641 | -34.52 | miRanda,TargetScan,RNAhybrid |
| HepG2 | KRTAP5-AS1 | hsa-miR-326 | 532 | 551 | -37.75 | miRanda,TargetScan,RNAhybrid |
| HepG2 | KRTAP5-AS1 | hsa-miR-326 | 391 | 410 | -37.75 | miRanda,TargetScan,RNAhybrid |
| HepG2 | LINC00668 | hsa-miR-326 | 28 | 48 | -32.78 | miRanda,TargetScan,RNAhybrid |
| HepG2 | LINC01270 | hsa-miR-326 | 60 | 79 | -34.19 | miRanda,TargetScan,RNAhybrid |
| HepG2 | LINC01270 | hsa-miR-326 | 49 | 68 | -34.19 | miRanda,TargetScan,RNAhybrid |
| HepG2 | LINC01270 | hsa-miR-326 | 20 | 39 | -34.19 | miRanda,TargetScan,RNAhybrid |
| HepG2 | DARS-AS1 | hsa-miR-331-3p | 199 | 219 | -25.99 | miRanda,TargetScan,RNAhybrid |
| HepG2 | DARS-AS1 | hsa-miR-331-3p | 172 | 192 | -25.99 | miRanda,TargetScan,RNAhybrid |
| HepG2 | DARS-AS1 | hsa-miR-331-3p | 171 | 191 | -25.99 | miRanda,TargetScan,RNAhybrid |
| HepG2 | DARS-AS1 | hsa-miR-331-3p | 155 | 175 | -25.99 | miRanda,TargetScan,RNAhybrid |
| HepG2 | DARS-AS1 | hsa-miR-331-3p | 129 | 149 | -25.99 | miRanda,TargetScan,RNAhybrid |
| HepG2 | DARS-AS1 | hsa-miR-331-3p | 14 | 34 | -25.99 | miRanda,TargetScan,RNAhybrid |
| HepG2 | FAM85B | hsa-miR-331-3p | 59 | 79 | -33.94 | miRanda,TargetScan,RNAhybrid |
| HepG2 | FAM85B | hsa-miR-331-3p | 10 | 30 | -33.94 | miRanda,TargetScan,RNAhybrid |
| HepG2 | MEG3 | hsa-miR-331-3p | 235 | 255 | -31.44 | miRanda,TargetScan,RNAhybrid |
| HepG2 | DIO3OS | hsa-miR-331-3p | 188 | 208 | -28.62 | miRanda,TargetScan,RNAhybrid |
| HepG2 | DIO3OS | hsa-miR-331-3p | 274 | 294 | -28.62 | miRanda,TargetScan,RNAhybrid |
| HepG2 | FAM157C | hsa-miR-331-3p | 1169 | 1189 | -30.43 | miRanda,TargetScan,RNAhybrid |
| HepG2 | FAM157C | hsa-miR-331-3p | 1006 | 1026 | -30.43 | miRanda,TargetScan,RNAhybrid |
| HepG2 | FAM157C | hsa-miR-331-3p | 1111 | 1131 | -30.43 | miRanda,TargetScan,RNAhybrid |
| HepG2 | FAM157C | hsa-miR-331-3p | 490 | 510 | -30.43 | miRanda,TargetScan,RNAhybrid |
| HepG2 | FAM157C | hsa-miR-331-3p | 21 | 41 | -30.43 | miRanda,TargetScan,RNAhybrid |
| HepG2 | LINC00671 | hsa-miR-331-3p | 256 | 276 | -26.15 | miRanda,TargetScan,RNAhybrid |
| HepG2 | LINC00313 | hsa-miR-331-3p | 208 | 228 | -30.15 | miRanda,TargetScan,RNAhybrid |
| HepG2 | LINC00313 | hsa-miR-331-3p | 1138 | 1158 | -30.15 | miRanda,TargetScan,RNAhybrid |
| HepG2 | LINC00313 | hsa-miR-331-3p | 592 | 612 | -30.15 | miRanda,TargetScan,RNAhybrid |
| HepG2 | PCAT14 | hsa-miR-331-3p | 772 | 792 | -31.91 | miRanda,TargetScan,RNAhybrid |
| HepG2 | MAGI2-AS3 | hsa-miR-339-5p | 67 | 89 | -31.61 | miRanda,TargetScan,RNAhybrid |
| HepG2 | RAB11B-AS1 | hsa-miR-564 | 328 | 346 | -28.65 | miRanda,TargetScan,RNAhybrid |
| HepG2 | RAB11B-AS1 | hsa-miR-564 | 373 | 391 | -28.65 | miRanda,TargetScan,RNAhybrid |
| HepG2 | RAB11B-AS1 | hsa-miR-564 | 813 | 831 | -28.65 | miRanda,TargetScan,RNAhybrid |
| HepG2 | RAB11B-AS1 | hsa-miR-564 | 280 | 298 | -28.65 | miRanda,TargetScan,RNAhybrid |
| HepG2 | SNHG14 | hsa-miR-574-5p | 136 | 158 | -27.25 | miRanda,TargetScan,RNAhybrid |
| HepG2 | SNHG14 | hsa-miR-574-5p | 19 | 41 | -27.25 | miRanda,TargetScan,RNAhybrid |
| HepG2 | LINC00313 | hsa-miR-650 | 1560 | 1580 | -34.19 | miRanda,TargetScan,RNAhybrid |
| HepG2 | GACAT3 | hsa-miR-657 | 191 | 213 | -31.17 | miRanda,TargetScan,RNAhybrid |
| HepG2 | LAMTOR5-AS1 | hsa-miR-761 | 242 | 263 | -32.26 | miRanda,TargetScan,RNAhybrid |
| HepG2 | LINC01515 | hsa-miR-761 | 370 | 392 | -23.56 | miRanda,TargetScan,RNAhybrid |
| HepG2 | SNHG4 | hsa-miR-3170 | 252 | 273 | -27.25 | miRanda,TargetScan,RNAhybrid |
| HepG2 | SNHG14 | hsa-miR-3176 | 313 | 331 | -32.49 | miRanda,TargetScan,RNAhybrid |
| HepG2 | SNHG14 | hsa-miR-3176 | 300 | 318 | -32.49 | miRanda,TargetScan,RNAhybrid |
| HepG2 | SNHG14 | hsa-miR-3176 | 30 | 48 | -26.12 | miRanda,TargetScan,RNAhybrid |
| HepG2 | SNHG12 | hsa-miR-3178 | 297 | 313 | -31.38 | miRanda,TargetScan,RNAhybrid |
| HepG2 | SNHG12 | hsa-miR-3178 | 441 | 457 | -31.38 | miRanda,TargetScan,RNAhybrid |
| HepG2 | SNHG12 | hsa-miR-3178 | 544 | 560 | -31.38 | miRanda,TargetScan,RNAhybrid |
| HepG2 | SNHG12 | hsa-miR-3178 | 612 | 628 | -31.38 | miRanda,TargetScan,RNAhybrid |
| HepG2 | SNHG12 | hsa-miR-3178 | 724 | 740 | -31.38 | miRanda,TargetScan,RNAhybrid |
| HepG2 | SNHG12 | hsa-miR-3178 | 932 | 948 | -31.38 | miRanda,TargetScan,RNAhybrid |
| HepG2 | SNHG12 | hsa-miR-3178 | 1255 | 1271 | -31.38 | miRanda,TargetScan,RNAhybrid |
| HepG2 | SNHG12 | hsa-miR-3178 | 213 | 229 | -31.38 | miRanda,TargetScan,RNAhybrid |
| HepG2 | SNHG12 | hsa-miR-3178 | 47 | 63 | -31.38 | miRanda,TargetScan,RNAhybrid |
| HepG2 | SNHG12 | hsa-miR-3178 | 186 | 202 | -31.38 | miRanda,TargetScan,RNAhybrid |
| HepG2 | SNHG12 | hsa-miR-3178 | 194 | 210 | -31.38 | miRanda,TargetScan,RNAhybrid |
| HepG2 | SNHG12 | hsa-miR-3178 | 205 | 221 | -31.38 | miRanda,TargetScan,RNAhybrid |
| HepG2 | SNHG12 | hsa-miR-3178 | 206 | 222 | -31.38 | miRanda,TargetScan,RNAhybrid |
| HepG2 | SNHG12 | hsa-miR-3178 | 209 | 225 | -31.38 | miRanda,TargetScan,RNAhybrid |
| HepG2 | SNHG12 | hsa-miR-3178 | 224 | 240 | -31.38 | miRanda,TargetScan,RNAhybrid |
| HepG2 | SNHG12 | hsa-miR-3178 | 225 | 241 | -31.38 | miRanda,TargetScan,RNAhybrid |
| HepG2 | SNHG12 | hsa-miR-3178 | 230 | 246 | -31.38 | miRanda,TargetScan,RNAhybrid |
| HepG2 | SNHG12 | hsa-miR-3178 | 15 | 31 | -31.38 | miRanda,TargetScan,RNAhybrid |
| HepG2 | SNHG12 | hsa-miR-3178 | 170 | 186 | -31.38 | miRanda,TargetScan,RNAhybrid |
| HepG2 | SNHG12 | hsa-miR-3178 | 149 | 165 | -31.38 | miRanda,TargetScan,RNAhybrid |
| HepG2 | SNHG12 | hsa-miR-3178 | 219 | 235 | -31.38 | miRanda,TargetScan,RNAhybrid |
| HepG2 | SNHG12 | hsa-miR-3178 | 223 | 239 | -31.38 | miRanda,TargetScan,RNAhybrid |
| HepG2 | CAHM | hsa-miR-3178 | 35 | 53 | -32.32 | miRanda,TargetScan,RNAhybrid |
| HepG2 | MMP25-AS1 | hsa-miR-3178 | 867 | 883 | -36.22 | miRanda,TargetScan,RNAhybrid |
| HepG2 | MMP25-AS1 | hsa-miR-3178 | 840 | 856 | -36.22 | miRanda,TargetScan,RNAhybrid |
| HepG2 | LINC00910 | hsa-miR-3178 | 517 | 533 | -28.76 | miRanda,TargetScan,RNAhybrid |
| HepG2 | LINC00910 | hsa-miR-3178 | 542 | 558 | -28.76 | miRanda,TargetScan,RNAhybrid |
| HepG2 | LINC00910 | hsa-miR-3178 | 857 | 873 | -28.76 | miRanda,TargetScan,RNAhybrid |
| HepG2 | LINC00910 | hsa-miR-3178 | 648 | 664 | -28.76 | miRanda,TargetScan,RNAhybrid |
| HepG2 | LINC00910 | hsa-miR-3178 | 577 | 593 | -28.76 | miRanda,TargetScan,RNAhybrid |
| HepG2 | LINC00910 | hsa-miR-3178 | 506 | 522 | -28.76 | miRanda,TargetScan,RNAhybrid |
| HepG2 | LINC00910 | hsa-miR-3178 | 529 | 545 | -28.76 | miRanda,TargetScan,RNAhybrid |
| HepG2 | LINC00910 | hsa-miR-3178 | 257 | 273 | -28.76 | miRanda,TargetScan,RNAhybrid |
| HepG2 | ASB16-AS1 | hsa-miR-3178 | 851 | 867 | -32.15 | miRanda,TargetScan,RNAhybrid |
| HepG2 | ZNF582-AS1 | hsa-miR-3178 | 155 | 171 | -33.98 | miRanda,TargetScan,RNAhybrid |
| HepG2 | HOXA-AS2 | hsa-miR-3195 | 476 | 492 | -30.42 | miRanda,TargetScan,RNAhybrid |
| HepG2 | ZMIZ1-AS1 | hsa-miR-3195 | 601 | 617 | -35.09 | miRanda,TargetScan,RNAhybrid |
| HepG2 | NR2F2-AS1 | hsa-miR-3195 | 76 | 92 | -28.24 | miRanda,TargetScan,RNAhybrid |
| HepG2 | NR2F2-AS1 | hsa-miR-3195 | 26 | 42 | -28.24 | miRanda,TargetScan,RNAhybrid |
| HepG2 | NR2F2-AS1 | hsa-miR-3195 | 74 | 90 | -28.24 | miRanda,TargetScan,RNAhybrid |
| HepG2 | NR2F2-AS1 | hsa-miR-3195 | 55 | 71 | -28.24 | miRanda,TargetScan,RNAhybrid |
| HepG2 | NR2F2-AS1 | hsa-miR-3195 | 87 | 103 | -28.24 | miRanda,TargetScan,RNAhybrid |
| HepG2 | SNAI3-AS1 | hsa-miR-3195 | 75 | 91 | -31.24 | miRanda,TargetScan,RNAhybrid |
| HepG2 | LINC00894 | hsa-miR-3195 | 908 | 924 | -33.3 | miRanda,TargetScan,RNAhybrid |
| HepG2 | DIO3OS | hsa-miR-3198 | 71 | 92 | -37.9 | miRanda,TargetScan,RNAhybrid |
| HepG2 | DIO3OS | hsa-miR-3198 | 152 | 173 | -37.9 | miRanda,TargetScan,RNAhybrid |
| HepG2 | DIO3OS | hsa-miR-3198 | 270 | 291 | -37.9 | miRanda,TargetScan,RNAhybrid |
| HepG2 | DIO3OS | hsa-miR-3198 | 281 | 302 | -37.9 | miRanda,TargetScan,RNAhybrid |
| HepG2 | DIO3OS | hsa-miR-3198 | 636 | 657 | -37.9 | miRanda,TargetScan,RNAhybrid |
| HepG2 | DIO3OS | hsa-miR-3198 | 722 | 743 | -37.9 | miRanda,TargetScan,RNAhybrid |
| HepG2 | MMP25-AS1 | hsa-miR-4286 | 190 | 206 | -26.51 | miRanda,TargetScan,RNAhybrid |
| HepG2 | RAB11B-AS1 | hsa-miR-4286 | 196 | 212 | -29.7 | miRanda,TargetScan,RNAhybrid |
| HepG2 | LINC01239 | hsa-miR-331-3p | 603 | 623 | -29.48 | miRanda,TargetScan,RNAhybrid |
| HepG2 | LINC01239 | hsa-miR-331-3p | 184 | 204 | -29.48 | miRanda,TargetScan,RNAhybrid |
| HepG2 | LINC01239 | hsa-miR-331-3p | 126 | 146 | -29.48 | miRanda,TargetScan,RNAhybrid |
| HepG2 | PWAR5 | hsa-miR-574-5p | 136 | 158 | -27.25 | miRanda,TargetScan,RNAhybrid |
| HepG2 | PWAR5 | hsa-miR-574-5p | 19 | 41 | -27.25 | miRanda,TargetScan,RNAhybrid |
| HepG2 | PWAR5 | hsa-miR-3176 | 313 | 331 | -32.49 | miRanda,TargetScan,RNAhybrid |
| HepG2 | PWAR5 | hsa-miR-3176 | 300 | 318 | -32.49 | miRanda,TargetScan,RNAhybrid |
| HepG2 | PWAR5 | hsa-miR-3176 | 30 | 48 | -26.12 | miRanda,TargetScan,RNAhybrid |
| HepG2 | LINC01151 | hsa-miR-4284 | 337 | 354 | -22.84 | miRanda,TargetScan,RNAhybrid |
| HepG2 | PWAR1 | hsa-miR-574-5p | 136 | 158 | -27.25 | miRanda,TargetScan,RNAhybrid |
| HepG2 | PWAR1 | hsa-miR-574-5p | 19 | 41 | -27.25 | miRanda,TargetScan,RNAhybrid |
| HepG2 | PWAR1 | hsa-miR-3176 | 313 | 331 | -32.49 | miRanda,TargetScan,RNAhybrid |
| HepG2 | PWAR1 | hsa-miR-3176 | 300 | 318 | -32.49 | miRanda,TargetScan,RNAhybrid |
| HepG2 | PWAR1 | hsa-miR-3176 | 30 | 48 | -26.12 | miRanda,TargetScan,RNAhybrid |
| HT1080 | LINC01270 | hsa-miR-326 | 60 | 79 | -34.19 | miRanda,TargetScan,RNAhybrid |
| HT1080 | LINC01270 | hsa-miR-326 | 49 | 68 | -34.19 | miRanda,TargetScan,RNAhybrid |
| HT1080 | LINC01270 | hsa-miR-326 | 20 | 39 | -34.19 | miRanda,TargetScan,RNAhybrid |
| HT1080 | CDKN2B-AS1 | hsa-miR-331-3p | 603 | 623 | -29.48 | miRanda,TargetScan,RNAhybrid |
| HT1080 | CDKN2B-AS1 | hsa-miR-331-3p | 184 | 204 | -29.48 | miRanda,TargetScan,RNAhybrid |
| HT1080 | CDKN2B-AS1 | hsa-miR-331-3p | 126 | 146 | -29.48 | miRanda,TargetScan,RNAhybrid |
| HT1080 | MAGI2-AS3 | hsa-miR-339-5p | 67 | 89 | -31.61 | miRanda,TargetScan,RNAhybrid |
| HT1080 | SNHG14 | hsa-miR-574-5p | 136 | 158 | -27.25 | miRanda,TargetScan,RNAhybrid |
| HT1080 | SNHG14 | hsa-miR-574-5p | 19 | 41 | -27.25 | miRanda,TargetScan,RNAhybrid |
| HT1080 | MIR222HG | hsa-miR-574-5p | 290 | 312 | -31.92 | miRanda,TargetScan,RNAhybrid |
| HT1080 | PTOV1-AS2 | hsa-miR-612 | 205 | 234 | -37.47 | miRanda,TargetScan,RNAhybrid |
| HT1080 | PTOV1-AS2 | hsa-miR-612 | 216 | 245 | -37.47 | miRanda,TargetScan,RNAhybrid |
| HT1080 | LINC01126 | hsa-miR-663a | 459 | 481 | -37.78 | miRanda,TargetScan,RNAhybrid |
| HT1080 | LAMTOR5-AS1 | hsa-miR-761 | 242 | 263 | -32.26 | miRanda,TargetScan,RNAhybrid |
| HT1080 | A1BG-AS1 | hsa-miR-1204 | 67 | 89 | -32.56 | miRanda,TargetScan,RNAhybrid |
| HT1080 | A1BG-AS1 | hsa-miR-1204 | 81 | 101 | -23.54 | miRanda,TargetScan,RNAhybrid |
| HT1080 | SNHG4 | hsa-miR-3170 | 252 | 273 | -27.25 | miRanda,TargetScan,RNAhybrid |
| HT1080 | SNHG14 | hsa-miR-3176 | 313 | 331 | -32.49 | miRanda,TargetScan,RNAhybrid |
| HT1080 | SNHG14 | hsa-miR-3176 | 300 | 318 | -32.49 | miRanda,TargetScan,RNAhybrid |
| HT1080 | SNHG14 | hsa-miR-3176 | 30 | 48 | -26.12 | miRanda,TargetScan,RNAhybrid |
| HT1080 | SNHG12 | hsa-miR-3178 | 297 | 313 | -31.38 | miRanda,TargetScan,RNAhybrid |
| HT1080 | SNHG12 | hsa-miR-3178 | 441 | 457 | -31.38 | miRanda,TargetScan,RNAhybrid |
| HT1080 | SNHG12 | hsa-miR-3178 | 544 | 560 | -31.38 | miRanda,TargetScan,RNAhybrid |
| HT1080 | SNHG12 | hsa-miR-3178 | 612 | 628 | -31.38 | miRanda,TargetScan,RNAhybrid |
| HT1080 | SNHG12 | hsa-miR-3178 | 724 | 740 | -31.38 | miRanda,TargetScan,RNAhybrid |
| HT1080 | SNHG12 | hsa-miR-3178 | 932 | 948 | -31.38 | miRanda,TargetScan,RNAhybrid |
| HT1080 | SNHG12 | hsa-miR-3178 | 1255 | 1271 | -31.38 | miRanda,TargetScan,RNAhybrid |
| HT1080 | SNHG12 | hsa-miR-3178 | 213 | 229 | -31.38 | miRanda,TargetScan,RNAhybrid |
| HT1080 | SNHG12 | hsa-miR-3178 | 47 | 63 | -31.38 | miRanda,TargetScan,RNAhybrid |
| HT1080 | SNHG12 | hsa-miR-3178 | 186 | 202 | -31.38 | miRanda,TargetScan,RNAhybrid |
| HT1080 | SNHG12 | hsa-miR-3178 | 194 | 210 | -31.38 | miRanda,TargetScan,RNAhybrid |
| HT1080 | SNHG12 | hsa-miR-3178 | 205 | 221 | -31.38 | miRanda,TargetScan,RNAhybrid |
| HT1080 | SNHG12 | hsa-miR-3178 | 206 | 222 | -31.38 | miRanda,TargetScan,RNAhybrid |
| HT1080 | SNHG12 | hsa-miR-3178 | 209 | 225 | -31.38 | miRanda,TargetScan,RNAhybrid |
| HT1080 | SNHG12 | hsa-miR-3178 | 224 | 240 | -31.38 | miRanda,TargetScan,RNAhybrid |
| HT1080 | SNHG12 | hsa-miR-3178 | 225 | 241 | -31.38 | miRanda,TargetScan,RNAhybrid |
| HT1080 | SNHG12 | hsa-miR-3178 | 230 | 246 | -31.38 | miRanda,TargetScan,RNAhybrid |
| HT1080 | SNHG12 | hsa-miR-3178 | 15 | 31 | -31.38 | miRanda,TargetScan,RNAhybrid |
| HT1080 | SNHG12 | hsa-miR-3178 | 170 | 186 | -31.38 | miRanda,TargetScan,RNAhybrid |
| HT1080 | SNHG12 | hsa-miR-3178 | 149 | 165 | -31.38 | miRanda,TargetScan,RNAhybrid |
| HT1080 | SNHG12 | hsa-miR-3178 | 219 | 235 | -31.38 | miRanda,TargetScan,RNAhybrid |
| HT1080 | SNHG12 | hsa-miR-3178 | 223 | 239 | -31.38 | miRanda,TargetScan,RNAhybrid |
| HT1080 | MMP25-AS1 | hsa-miR-3178 | 867 | 883 | -36.22 | miRanda,TargetScan,RNAhybrid |
| HT1080 | MMP25-AS1 | hsa-miR-3178 | 840 | 856 | -36.22 | miRanda,TargetScan,RNAhybrid |
| HT1080 | ASB16-AS1 | hsa-miR-3178 | 851 | 867 | -32.15 | miRanda,TargetScan,RNAhybrid |
| HT1080 | LINC00899 | hsa-miR-3178 | 25 | 41 | -36.65 | miRanda,TargetScan,RNAhybrid |
| HT1080 | LINC00899 | hsa-miR-3178 | 10 | 26 | -36.65 | miRanda,TargetScan,RNAhybrid |
| HT1080 | LINC00899 | hsa-miR-3178 | 88 | 104 | -36.65 | miRanda,TargetScan,RNAhybrid |
| HT1080 | LINC00899 | hsa-miR-3178 | 53 | 69 | -36.65 | miRanda,TargetScan,RNAhybrid |
| HT1080 | ZMIZ1-AS1 | hsa-miR-3195 | 601 | 617 | -35.09 | miRanda,TargetScan,RNAhybrid |
| HT1080 | POT1-AS1 | hsa-miR-4286 | 897 | 913 | -26.71 | miRanda,TargetScan,RNAhybrid |
| HT1080 | MMP25-AS1 | hsa-miR-4286 | 190 | 206 | -26.51 | miRanda,TargetScan,RNAhybrid |
| HT1080 | LINC01239 | hsa-miR-331-3p | 603 | 623 | -29.48 | miRanda,TargetScan,RNAhybrid |
| HT1080 | LINC01239 | hsa-miR-331-3p | 184 | 204 | -29.48 | miRanda,TargetScan,RNAhybrid |
| HT1080 | LINC01239 | hsa-miR-331-3p | 126 | 146 | -29.48 | miRanda,TargetScan,RNAhybrid |
| HT1080 | PWAR5 | hsa-miR-574-5p | 136 | 158 | -27.25 | miRanda,TargetScan,RNAhybrid |
| HT1080 | PWAR5 | hsa-miR-574-5p | 19 | 41 | -27.25 | miRanda,TargetScan,RNAhybrid |
| HT1080 | PWAR5 | hsa-miR-3176 | 313 | 331 | -32.49 | miRanda,TargetScan,RNAhybrid |
| HT1080 | PWAR5 | hsa-miR-3176 | 300 | 318 | -32.49 | miRanda,TargetScan,RNAhybrid |
| HT1080 | PWAR5 | hsa-miR-3176 | 30 | 48 | -26.12 | miRanda,TargetScan,RNAhybrid |
| HUVEC | LINC01600 | hsa-miR-326 | 685 | 704 | -34.52 | miRanda,TargetScan,RNAhybrid |
| HUVEC | LINC01600 | hsa-miR-326 | 622 | 641 | -34.52 | miRanda,TargetScan,RNAhybrid |
| HUVEC | LINC01270 | hsa-miR-326 | 60 | 79 | -34.19 | miRanda,TargetScan,RNAhybrid |
| HUVEC | LINC01270 | hsa-miR-326 | 49 | 68 | -34.19 | miRanda,TargetScan,RNAhybrid |
| HUVEC | LINC01270 | hsa-miR-326 | 20 | 39 | -34.19 | miRanda,TargetScan,RNAhybrid |
| HUVEC | DARS-AS1 | hsa-miR-331-3p | 199 | 219 | -25.99 | miRanda,TargetScan,RNAhybrid |
| HUVEC | DARS-AS1 | hsa-miR-331-3p | 172 | 192 | -25.99 | miRanda,TargetScan,RNAhybrid |
| HUVEC | DARS-AS1 | hsa-miR-331-3p | 171 | 191 | -25.99 | miRanda,TargetScan,RNAhybrid |
| HUVEC | DARS-AS1 | hsa-miR-331-3p | 155 | 175 | -25.99 | miRanda,TargetScan,RNAhybrid |
| HUVEC | DARS-AS1 | hsa-miR-331-3p | 129 | 149 | -25.99 | miRanda,TargetScan,RNAhybrid |
| HUVEC | DARS-AS1 | hsa-miR-331-3p | 14 | 34 | -25.99 | miRanda,TargetScan,RNAhybrid |
| HUVEC | CDKN2B-AS1 | hsa-miR-331-3p | 603 | 623 | -29.48 | miRanda,TargetScan,RNAhybrid |
| HUVEC | CDKN2B-AS1 | hsa-miR-331-3p | 184 | 204 | -29.48 | miRanda,TargetScan,RNAhybrid |
| HUVEC | CDKN2B-AS1 | hsa-miR-331-3p | 126 | 146 | -29.48 | miRanda,TargetScan,RNAhybrid |
| HUVEC | FAM157C | hsa-miR-331-3p | 1169 | 1189 | -30.43 | miRanda,TargetScan,RNAhybrid |
| HUVEC | FAM157C | hsa-miR-331-3p | 1006 | 1026 | -30.43 | miRanda,TargetScan,RNAhybrid |
| HUVEC | FAM157C | hsa-miR-331-3p | 1111 | 1131 | -30.43 | miRanda,TargetScan,RNAhybrid |
| HUVEC | FAM157C | hsa-miR-331-3p | 490 | 510 | -30.43 | miRanda,TargetScan,RNAhybrid |
| HUVEC | FAM157C | hsa-miR-331-3p | 21 | 41 | -30.43 | miRanda,TargetScan,RNAhybrid |
| HUVEC | NARF-IT1 | hsa-miR-331-3p | 281 | 301 | -36.15 | miRanda,TargetScan,RNAhybrid |
| HUVEC | LINC00313 | hsa-miR-331-3p | 208 | 228 | -30.15 | miRanda,TargetScan,RNAhybrid |
| HUVEC | LINC00313 | hsa-miR-331-3p | 1138 | 1158 | -30.15 | miRanda,TargetScan,RNAhybrid |
| HUVEC | LINC00313 | hsa-miR-331-3p | 592 | 612 | -30.15 | miRanda,TargetScan,RNAhybrid |
| HUVEC | MAGI2-AS3 | hsa-miR-339-5p | 67 | 89 | -31.61 | miRanda,TargetScan,RNAhybrid |
| HUVEC | SNHG14 | hsa-miR-574-5p | 136 | 158 | -27.25 | miRanda,TargetScan,RNAhybrid |
| HUVEC | SNHG14 | hsa-miR-574-5p | 19 | 41 | -27.25 | miRanda,TargetScan,RNAhybrid |
| HUVEC | SEMA3F-AS1 | hsa-miR-650 | 102 | 122 | -27.92 | miRanda,TargetScan,RNAhybrid |
| HUVEC | LINC00313 | hsa-miR-650 | 1560 | 1580 | -34.19 | miRanda,TargetScan,RNAhybrid |
| HUVEC | LINC01126 | hsa-miR-663a | 459 | 481 | -37.78 | miRanda,TargetScan,RNAhybrid |
| HUVEC | LAMTOR5-AS1 | hsa-miR-761 | 242 | 263 | -32.26 | miRanda,TargetScan,RNAhybrid |
| HUVEC | LINC01515 | hsa-miR-761 | 370 | 392 | -23.56 | miRanda,TargetScan,RNAhybrid |
| HUVEC | SPANXA2-OT1 | hsa-miR-940 | 359 | 379 | -32.32 | miRanda,TargetScan,RNAhybrid |
| HUVEC | SNHG4 | hsa-miR-3170 | 252 | 273 | -27.25 | miRanda,TargetScan,RNAhybrid |
| HUVEC | SNHG14 | hsa-miR-3176 | 313 | 331 | -32.49 | miRanda,TargetScan,RNAhybrid |
| HUVEC | SNHG14 | hsa-miR-3176 | 300 | 318 | -32.49 | miRanda,TargetScan,RNAhybrid |
| HUVEC | SNHG14 | hsa-miR-3176 | 30 | 48 | -26.12 | miRanda,TargetScan,RNAhybrid |
| HUVEC | SNHG12 | hsa-miR-3178 | 297 | 313 | -31.38 | miRanda,TargetScan,RNAhybrid |
| HUVEC | SNHG12 | hsa-miR-3178 | 441 | 457 | -31.38 | miRanda,TargetScan,RNAhybrid |
| HUVEC | SNHG12 | hsa-miR-3178 | 544 | 560 | -31.38 | miRanda,TargetScan,RNAhybrid |
| HUVEC | SNHG12 | hsa-miR-3178 | 612 | 628 | -31.38 | miRanda,TargetScan,RNAhybrid |
| HUVEC | SNHG12 | hsa-miR-3178 | 724 | 740 | -31.38 | miRanda,TargetScan,RNAhybrid |
| HUVEC | SNHG12 | hsa-miR-3178 | 932 | 948 | -31.38 | miRanda,TargetScan,RNAhybrid |
| HUVEC | SNHG12 | hsa-miR-3178 | 1255 | 1271 | -31.38 | miRanda,TargetScan,RNAhybrid |
| HUVEC | SNHG12 | hsa-miR-3178 | 213 | 229 | -31.38 | miRanda,TargetScan,RNAhybrid |
| HUVEC | SNHG12 | hsa-miR-3178 | 47 | 63 | -31.38 | miRanda,TargetScan,RNAhybrid |
| HUVEC | SNHG12 | hsa-miR-3178 | 186 | 202 | -31.38 | miRanda,TargetScan,RNAhybrid |
| HUVEC | SNHG12 | hsa-miR-3178 | 194 | 210 | -31.38 | miRanda,TargetScan,RNAhybrid |
| HUVEC | SNHG12 | hsa-miR-3178 | 205 | 221 | -31.38 | miRanda,TargetScan,RNAhybrid |
| HUVEC | SNHG12 | hsa-miR-3178 | 206 | 222 | -31.38 | miRanda,TargetScan,RNAhybrid |
| HUVEC | SNHG12 | hsa-miR-3178 | 209 | 225 | -31.38 | miRanda,TargetScan,RNAhybrid |
| HUVEC | SNHG12 | hsa-miR-3178 | 224 | 240 | -31.38 | miRanda,TargetScan,RNAhybrid |
| HUVEC | SNHG12 | hsa-miR-3178 | 225 | 241 | -31.38 | miRanda,TargetScan,RNAhybrid |
| HUVEC | SNHG12 | hsa-miR-3178 | 230 | 246 | -31.38 | miRanda,TargetScan,RNAhybrid |
| HUVEC | SNHG12 | hsa-miR-3178 | 15 | 31 | -31.38 | miRanda,TargetScan,RNAhybrid |
| HUVEC | SNHG12 | hsa-miR-3178 | 170 | 186 | -31.38 | miRanda,TargetScan,RNAhybrid |
| HUVEC | SNHG12 | hsa-miR-3178 | 149 | 165 | -31.38 | miRanda,TargetScan,RNAhybrid |
| HUVEC | SNHG12 | hsa-miR-3178 | 219 | 235 | -31.38 | miRanda,TargetScan,RNAhybrid |
| HUVEC | SNHG12 | hsa-miR-3178 | 223 | 239 | -31.38 | miRanda,TargetScan,RNAhybrid |
| HUVEC | CAHM | hsa-miR-3178 | 35 | 53 | -32.32 | miRanda,TargetScan,RNAhybrid |
| HUVEC | MMP25-AS1 | hsa-miR-3178 | 867 | 883 | -36.22 | miRanda,TargetScan,RNAhybrid |
| HUVEC | MMP25-AS1 | hsa-miR-3178 | 840 | 856 | -36.22 | miRanda,TargetScan,RNAhybrid |
| HUVEC | LINC00910 | hsa-miR-3178 | 517 | 533 | -28.76 | miRanda,TargetScan,RNAhybrid |
| HUVEC | LINC00910 | hsa-miR-3178 | 542 | 558 | -28.76 | miRanda,TargetScan,RNAhybrid |
| HUVEC | LINC00910 | hsa-miR-3178 | 857 | 873 | -28.76 | miRanda,TargetScan,RNAhybrid |
| HUVEC | LINC00910 | hsa-miR-3178 | 648 | 664 | -28.76 | miRanda,TargetScan,RNAhybrid |
| HUVEC | LINC00910 | hsa-miR-3178 | 577 | 593 | -28.76 | miRanda,TargetScan,RNAhybrid |
| HUVEC | LINC00910 | hsa-miR-3178 | 506 | 522 | -28.76 | miRanda,TargetScan,RNAhybrid |
| HUVEC | LINC00910 | hsa-miR-3178 | 529 | 545 | -28.76 | miRanda,TargetScan,RNAhybrid |
| HUVEC | LINC00910 | hsa-miR-3178 | 257 | 273 | -28.76 | miRanda,TargetScan,RNAhybrid |
| HUVEC | ASB16-AS1 | hsa-miR-3178 | 851 | 867 | -32.15 | miRanda,TargetScan,RNAhybrid |
| HUVEC | ZNF582-AS1 | hsa-miR-3178 | 155 | 171 | -33.98 | miRanda,TargetScan,RNAhybrid |
| HUVEC | LINC00899 | hsa-miR-3178 | 25 | 41 | -36.65 | miRanda,TargetScan,RNAhybrid |
| HUVEC | LINC00899 | hsa-miR-3178 | 10 | 26 | -36.65 | miRanda,TargetScan,RNAhybrid |
| HUVEC | LINC00899 | hsa-miR-3178 | 88 | 104 | -36.65 | miRanda,TargetScan,RNAhybrid |
| HUVEC | LINC00899 | hsa-miR-3178 | 53 | 69 | -36.65 | miRanda,TargetScan,RNAhybrid |
| HUVEC | PAX8-AS1 | hsa-miR-320e | 15 | 32 | -25.81 | miRanda,TargetScan,RNAhybrid |
| HUVEC | IGFBP7-AS1 | hsa-miR-3195 | 193 | 209 | -31.14 | miRanda,TargetScan,RNAhybrid |
| HUVEC | HOXA-AS2 | hsa-miR-3195 | 476 | 492 | -30.42 | miRanda,TargetScan,RNAhybrid |
| HUVEC | NR2F2-AS1 | hsa-miR-3195 | 76 | 92 | -28.24 | miRanda,TargetScan,RNAhybrid |
| HUVEC | NR2F2-AS1 | hsa-miR-3195 | 26 | 42 | -28.24 | miRanda,TargetScan,RNAhybrid |
| HUVEC | NR2F2-AS1 | hsa-miR-3195 | 74 | 90 | -28.24 | miRanda,TargetScan,RNAhybrid |
| HUVEC | NR2F2-AS1 | hsa-miR-3195 | 55 | 71 | -28.24 | miRanda,TargetScan,RNAhybrid |
| HUVEC | NR2F2-AS1 | hsa-miR-3195 | 87 | 103 | -28.24 | miRanda,TargetScan,RNAhybrid |
| HUVEC | SNAI3-AS1 | hsa-miR-3195 | 75 | 91 | -31.24 | miRanda,TargetScan,RNAhybrid |
| HUVEC | GATA6-AS1 | hsa-miR-3195 | 119 | 136 | -35.28 | miRanda,TargetScan,RNAhybrid |
| HUVEC | LINC00894 | hsa-miR-3195 | 908 | 924 | -33.3 | miRanda,TargetScan,RNAhybrid |
| HUVEC | HOXA11-AS | hsa-miR-4286 | 208 | 224 | -25.92 | miRanda,TargetScan,RNAhybrid |
| HUVEC | MMP25-AS1 | hsa-miR-4286 | 190 | 206 | -26.51 | miRanda,TargetScan,RNAhybrid |
| HUVEC | CACTIN-AS1 | hsa-miR-4286 | 425 | 441 | -29.48 | miRanda,TargetScan,RNAhybrid |
| HUVEC | CACTIN-AS1 | hsa-miR-4286 | 410 | 426 | -29.48 | miRanda,TargetScan,RNAhybrid |
| HUVEC | CACTIN-AS1 | hsa-miR-4286 | 406 | 422 | -29.48 | miRanda,TargetScan,RNAhybrid |
| HUVEC | CACTIN-AS1 | hsa-miR-4286 | 79 | 95 | -29.48 | miRanda,TargetScan,RNAhybrid |
| HUVEC | MEG8 | hsa-miR-331-3p | 235 | 255 | -31.44 | miRanda,TargetScan,RNAhybrid |
| HUVEC | PWAR5 | hsa-miR-574-5p | 136 | 158 | -27.25 | miRanda,TargetScan,RNAhybrid |
| HUVEC | PWAR5 | hsa-miR-574-5p | 19 | 41 | -27.25 | miRanda,TargetScan,RNAhybrid |
| HUVEC | FAM66B | hsa-miR-612 | 146 | 170 | -33.24 | miRanda,TargetScan,RNAhybrid |
| HUVEC | PWAR5 | hsa-miR-3176 | 313 | 331 | -32.49 | miRanda,TargetScan,RNAhybrid |
| HUVEC | PWAR5 | hsa-miR-3176 | 300 | 318 | -32.49 | miRanda,TargetScan,RNAhybrid |
| HUVEC | PWAR5 | hsa-miR-3176 | 30 | 48 | -26.12 | miRanda,TargetScan,RNAhybrid |
| IMR.90 | LINC01270 | hsa-miR-326 | 60 | 79 | -34.19 | miRanda,TargetScan,RNAhybrid |
| IMR.90 | LINC01270 | hsa-miR-326 | 49 | 68 | -34.19 | miRanda,TargetScan,RNAhybrid |
| IMR.90 | LINC01270 | hsa-miR-326 | 20 | 39 | -34.19 | miRanda,TargetScan,RNAhybrid |
| IMR.90 | MRGPRF-AS1 | hsa-miR-331-3p | 564 | 584 | -28.13 | miRanda,TargetScan,RNAhybrid |
| IMR.90 | MRGPRF-AS1 | hsa-miR-331-3p | 138 | 158 | -28.13 | miRanda,TargetScan,RNAhybrid |
| IMR.90 | MEG3 | hsa-miR-331-3p | 235 | 255 | -31.44 | miRanda,TargetScan,RNAhybrid |
| IMR.90 | GATA2-AS1 | hsa-miR-3176 | 61 | 79 | -27.53 | miRanda,TargetScan,RNAhybrid |
| IMR.90 | GATA2-AS1 | hsa-miR-3176 | 48 | 66 | -27.53 | miRanda,TargetScan,RNAhybrid |
| IMR.90 | MMP25-AS1 | hsa-miR-3178 | 867 | 883 | -36.22 | miRanda,TargetScan,RNAhybrid |
| IMR.90 | MMP25-AS1 | hsa-miR-3178 | 840 | 856 | -36.22 | miRanda,TargetScan,RNAhybrid |
| IMR.90 | LINC00899 | hsa-miR-3178 | 25 | 41 | -36.65 | miRanda,TargetScan,RNAhybrid |
| IMR.90 | LINC00899 | hsa-miR-3178 | 10 | 26 | -36.65 | miRanda,TargetScan,RNAhybrid |
| IMR.90 | LINC00899 | hsa-miR-3178 | 88 | 104 | -36.65 | miRanda,TargetScan,RNAhybrid |
| IMR.90 | LINC00899 | hsa-miR-3178 | 53 | 69 | -36.65 | miRanda,TargetScan,RNAhybrid |
| IMR.90 | HAS2-AS1 | hsa-miR-3182 | 113 | 129 | -22.1 | miRanda,TargetScan,RNAhybrid |
| IMR.90 | HAS2-AS1 | hsa-miR-3182 | 111 | 127 | -22.1 | miRanda,TargetScan,RNAhybrid |
| IMR.90 | PAX8-AS1 | hsa-miR-320e | 15 | 32 | -25.81 | miRanda,TargetScan,RNAhybrid |
| IMR.90 | MMP25-AS1 | hsa-miR-4286 | 190 | 206 | -26.51 | miRanda,TargetScan,RNAhybrid |
| IMR.90 | MEG8 | hsa-miR-331-3p | 235 | 255 | -31.44 | miRanda,TargetScan,RNAhybrid |
| K562 | LINC01344 | hsa-miR-125a-3p | 471 | 493 | -39.65 | miRanda,TargetScan,RNAhybrid |
| K562 | DARS-AS1 | hsa-miR-331-3p | 199 | 219 | -25.99 | miRanda,TargetScan,RNAhybrid |
| K562 | DARS-AS1 | hsa-miR-331-3p | 172 | 192 | -25.99 | miRanda,TargetScan,RNAhybrid |
| K562 | DARS-AS1 | hsa-miR-331-3p | 171 | 191 | -25.99 | miRanda,TargetScan,RNAhybrid |
| K562 | DARS-AS1 | hsa-miR-331-3p | 155 | 175 | -25.99 | miRanda,TargetScan,RNAhybrid |
| K562 | DARS-AS1 | hsa-miR-331-3p | 129 | 149 | -25.99 | miRanda,TargetScan,RNAhybrid |
| K562 | DARS-AS1 | hsa-miR-331-3p | 14 | 34 | -25.99 | miRanda,TargetScan,RNAhybrid |
| K562 | NARF-IT1 | hsa-miR-331-3p | 281 | 301 | -36.15 | miRanda,TargetScan,RNAhybrid |
| K562 | ADPGK-AS1 | hsa-miR-484 | 31 | 52 | -32.06 | miRanda,TargetScan,RNAhybrid |
| K562 | PTOV1-AS2 | hsa-miR-612 | 205 | 234 | -37.47 | miRanda,TargetScan,RNAhybrid |
| K562 | PTOV1-AS2 | hsa-miR-612 | 216 | 245 | -37.47 | miRanda,TargetScan,RNAhybrid |
| K562 | ENTPD3-AS1 | hsa-miR-632 | 122 | 140 | -24.91 | miRanda,TargetScan,RNAhybrid |
| K562 | LINC01126 | hsa-miR-663a | 459 | 481 | -37.78 | miRanda,TargetScan,RNAhybrid |
| K562 | SNHG4 | hsa-miR-3170 | 252 | 273 | -27.25 | miRanda,TargetScan,RNAhybrid |
| K562 | SNHG12 | hsa-miR-3178 | 297 | 313 | -31.38 | miRanda,TargetScan,RNAhybrid |
| K562 | SNHG12 | hsa-miR-3178 | 441 | 457 | -31.38 | miRanda,TargetScan,RNAhybrid |
| K562 | SNHG12 | hsa-miR-3178 | 544 | 560 | -31.38 | miRanda,TargetScan,RNAhybrid |
| K562 | SNHG12 | hsa-miR-3178 | 612 | 628 | -31.38 | miRanda,TargetScan,RNAhybrid |
| K562 | SNHG12 | hsa-miR-3178 | 724 | 740 | -31.38 | miRanda,TargetScan,RNAhybrid |
| K562 | SNHG12 | hsa-miR-3178 | 932 | 948 | -31.38 | miRanda,TargetScan,RNAhybrid |
| K562 | SNHG12 | hsa-miR-3178 | 1255 | 1271 | -31.38 | miRanda,TargetScan,RNAhybrid |
| K562 | SNHG12 | hsa-miR-3178 | 213 | 229 | -31.38 | miRanda,TargetScan,RNAhybrid |
| K562 | SNHG12 | hsa-miR-3178 | 47 | 63 | -31.38 | miRanda,TargetScan,RNAhybrid |
| K562 | SNHG12 | hsa-miR-3178 | 186 | 202 | -31.38 | miRanda,TargetScan,RNAhybrid |
| K562 | SNHG12 | hsa-miR-3178 | 194 | 210 | -31.38 | miRanda,TargetScan,RNAhybrid |
| K562 | SNHG12 | hsa-miR-3178 | 205 | 221 | -31.38 | miRanda,TargetScan,RNAhybrid |
| K562 | SNHG12 | hsa-miR-3178 | 206 | 222 | -31.38 | miRanda,TargetScan,RNAhybrid |
| K562 | SNHG12 | hsa-miR-3178 | 209 | 225 | -31.38 | miRanda,TargetScan,RNAhybrid |
| K562 | SNHG12 | hsa-miR-3178 | 224 | 240 | -31.38 | miRanda,TargetScan,RNAhybrid |
| K562 | SNHG12 | hsa-miR-3178 | 225 | 241 | -31.38 | miRanda,TargetScan,RNAhybrid |
| K562 | SNHG12 | hsa-miR-3178 | 230 | 246 | -31.38 | miRanda,TargetScan,RNAhybrid |
| K562 | SNHG12 | hsa-miR-3178 | 15 | 31 | -31.38 | miRanda,TargetScan,RNAhybrid |
| K562 | SNHG12 | hsa-miR-3178 | 170 | 186 | -31.38 | miRanda,TargetScan,RNAhybrid |
| K562 | SNHG12 | hsa-miR-3178 | 149 | 165 | -31.38 | miRanda,TargetScan,RNAhybrid |
| K562 | SNHG12 | hsa-miR-3178 | 219 | 235 | -31.38 | miRanda,TargetScan,RNAhybrid |
| K562 | SNHG12 | hsa-miR-3178 | 223 | 239 | -31.38 | miRanda,TargetScan,RNAhybrid |
| K562 | CAHM | hsa-miR-3178 | 35 | 53 | -32.32 | miRanda,TargetScan,RNAhybrid |
| K562 | ZEB1-AS1 | hsa-miR-3178 | 260 | 276 | -31.96 | miRanda,TargetScan,RNAhybrid |
| K562 | MMP25-AS1 | hsa-miR-3178 | 867 | 883 | -36.22 | miRanda,TargetScan,RNAhybrid |
| K562 | MMP25-AS1 | hsa-miR-3178 | 840 | 856 | -36.22 | miRanda,TargetScan,RNAhybrid |
| K562 | LINC00910 | hsa-miR-3178 | 517 | 533 | -28.76 | miRanda,TargetScan,RNAhybrid |
| K562 | LINC00910 | hsa-miR-3178 | 542 | 558 | -28.76 | miRanda,TargetScan,RNAhybrid |
| K562 | LINC00910 | hsa-miR-3178 | 857 | 873 | -28.76 | miRanda,TargetScan,RNAhybrid |
| K562 | LINC00910 | hsa-miR-3178 | 648 | 664 | -28.76 | miRanda,TargetScan,RNAhybrid |
| K562 | LINC00910 | hsa-miR-3178 | 577 | 593 | -28.76 | miRanda,TargetScan,RNAhybrid |
| K562 | LINC00910 | hsa-miR-3178 | 506 | 522 | -28.76 | miRanda,TargetScan,RNAhybrid |
| K562 | LINC00910 | hsa-miR-3178 | 529 | 545 | -28.76 | miRanda,TargetScan,RNAhybrid |
| K562 | LINC00910 | hsa-miR-3178 | 257 | 273 | -28.76 | miRanda,TargetScan,RNAhybrid |
| K562 | PXN-AS1 | hsa-miR-3183 | 1013 | 1034 | -30.77 | miRanda,TargetScan,RNAhybrid |
| K562 | PXN-AS1 | hsa-miR-3183 | 1127 | 1148 | -30.77 | miRanda,TargetScan,RNAhybrid |
| K562 | PXN-AS1 | hsa-miR-3183 | 1110 | 1131 | -30.77 | miRanda,TargetScan,RNAhybrid |
| K562 | PXN-AS1 | hsa-miR-3183 | 1014 | 1035 | -30.77 | miRanda,TargetScan,RNAhybrid |
| K562 | PAX8-AS1 | hsa-miR-320e | 15 | 32 | -25.81 | miRanda,TargetScan,RNAhybrid |
| K562 | NR2F2-AS1 | hsa-miR-3195 | 76 | 92 | -28.24 | miRanda,TargetScan,RNAhybrid |
| K562 | NR2F2-AS1 | hsa-miR-3195 | 26 | 42 | -28.24 | miRanda,TargetScan,RNAhybrid |
| K562 | NR2F2-AS1 | hsa-miR-3195 | 74 | 90 | -28.24 | miRanda,TargetScan,RNAhybrid |
| K562 | NR2F2-AS1 | hsa-miR-3195 | 55 | 71 | -28.24 | miRanda,TargetScan,RNAhybrid |
| K562 | NR2F2-AS1 | hsa-miR-3195 | 87 | 103 | -28.24 | miRanda,TargetScan,RNAhybrid |
| K562 | SNAI3-AS1 | hsa-miR-3195 | 75 | 91 | -31.24 | miRanda,TargetScan,RNAhybrid |
| K562 | HOTAIR | hsa-miR-4286 | 275 | 291 | -22.5 | miRanda,TargetScan,RNAhybrid |
| K562 | MMP25-AS1 | hsa-miR-4286 | 190 | 206 | -26.51 | miRanda,TargetScan,RNAhybrid |
| K562 | FGD5-AS1 | hsa-miR-4289 | 83 | 101 | -26.18 | miRanda,TargetScan,RNAhybrid |
| K562 | FGD5-AS1 | hsa-miR-4289 | 1847 | 1865 | -26.18 | miRanda,TargetScan,RNAhybrid |
| MCF.7 | KRTAP5-AS1 | hsa-miR-326 | 532 | 551 | -37.75 | miRanda,TargetScan,RNAhybrid |
| MCF.7 | KRTAP5-AS1 | hsa-miR-326 | 391 | 410 | -37.75 | miRanda,TargetScan,RNAhybrid |
| MCF.7 | LINC01270 | hsa-miR-326 | 60 | 79 | -34.19 | miRanda,TargetScan,RNAhybrid |
| MCF.7 | LINC01270 | hsa-miR-326 | 49 | 68 | -34.19 | miRanda,TargetScan,RNAhybrid |
| MCF.7 | LINC01270 | hsa-miR-326 | 20 | 39 | -34.19 | miRanda,TargetScan,RNAhybrid |
| MCF.7 | DARS-AS1 | hsa-miR-331-3p | 199 | 219 | -25.99 | miRanda,TargetScan,RNAhybrid |
| MCF.7 | DARS-AS1 | hsa-miR-331-3p | 172 | 192 | -25.99 | miRanda,TargetScan,RNAhybrid |
| MCF.7 | DARS-AS1 | hsa-miR-331-3p | 171 | 191 | -25.99 | miRanda,TargetScan,RNAhybrid |
| MCF.7 | DARS-AS1 | hsa-miR-331-3p | 155 | 175 | -25.99 | miRanda,TargetScan,RNAhybrid |
| MCF.7 | DARS-AS1 | hsa-miR-331-3p | 129 | 149 | -25.99 | miRanda,TargetScan,RNAhybrid |
| MCF.7 | DARS-AS1 | hsa-miR-331-3p | 14 | 34 | -25.99 | miRanda,TargetScan,RNAhybrid |
| MCF.7 | CDKN2B-AS1 | hsa-miR-331-3p | 603 | 623 | -29.48 | miRanda,TargetScan,RNAhybrid |
| MCF.7 | CDKN2B-AS1 | hsa-miR-331-3p | 184 | 204 | -29.48 | miRanda,TargetScan,RNAhybrid |
| MCF.7 | CDKN2B-AS1 | hsa-miR-331-3p | 126 | 146 | -29.48 | miRanda,TargetScan,RNAhybrid |
| MCF.7 | CASC2 | hsa-miR-331-3p | 42 | 61 | -30.94 | miRanda,TargetScan,RNAhybrid |
| MCF.7 | DIO3OS | hsa-miR-331-3p | 188 | 208 | -28.62 | miRanda,TargetScan,RNAhybrid |
| MCF.7 | DIO3OS | hsa-miR-331-3p | 274 | 294 | -28.62 | miRanda,TargetScan,RNAhybrid |
| MCF.7 | FAM157C | hsa-miR-331-3p | 1169 | 1189 | -30.43 | miRanda,TargetScan,RNAhybrid |
| MCF.7 | FAM157C | hsa-miR-331-3p | 1006 | 1026 | -30.43 | miRanda,TargetScan,RNAhybrid |
| MCF.7 | FAM157C | hsa-miR-331-3p | 1111 | 1131 | -30.43 | miRanda,TargetScan,RNAhybrid |
| MCF.7 | FAM157C | hsa-miR-331-3p | 490 | 510 | -30.43 | miRanda,TargetScan,RNAhybrid |
| MCF.7 | FAM157C | hsa-miR-331-3p | 21 | 41 | -30.43 | miRanda,TargetScan,RNAhybrid |
| MCF.7 | LINC00313 | hsa-miR-331-3p | 208 | 228 | -30.15 | miRanda,TargetScan,RNAhybrid |
| MCF.7 | LINC00313 | hsa-miR-331-3p | 1138 | 1158 | -30.15 | miRanda,TargetScan,RNAhybrid |
| MCF.7 | LINC00313 | hsa-miR-331-3p | 592 | 612 | -30.15 | miRanda,TargetScan,RNAhybrid |
| MCF.7 | FEZF1-AS1 | hsa-miR-492 | 1360 | 1382 | -30.97 | miRanda,TargetScan,RNAhybrid |
| MCF.7 | MIR222HG | hsa-miR-574-5p | 290 | 312 | -31.92 | miRanda,TargetScan,RNAhybrid |
| MCF.7 | LINC00853 | hsa-miR-612 | 825 | 849 | -33.84 | miRanda,TargetScan,RNAhybrid |
| MCF.7 | LINC00853 | hsa-miR-612 | 833 | 857 | -33.84 | miRanda,TargetScan,RNAhybrid |
| MCF.7 | LINC00853 | hsa-miR-612 | 575 | 599 | -33.84 | miRanda,TargetScan,RNAhybrid |
| MCF.7 | LINC00853 | hsa-miR-612 | 786 | 810 | -33.84 | miRanda,TargetScan,RNAhybrid |
| MCF.7 | LINC00853 | hsa-miR-612 | 709 | 733 | -33.84 | miRanda,TargetScan,RNAhybrid |
| MCF.7 | LINC00853 | hsa-miR-612 | 481 | 505 | -33.84 | miRanda,TargetScan,RNAhybrid |
| MCF.7 | LINC00853 | hsa-miR-612 | 910 | 934 | -33.84 | miRanda,TargetScan,RNAhybrid |
| MCF.7 | PTOV1-AS2 | hsa-miR-612 | 205 | 234 | -37.47 | miRanda,TargetScan,RNAhybrid |
| MCF.7 | PTOV1-AS2 | hsa-miR-612 | 216 | 245 | -37.47 | miRanda,TargetScan,RNAhybrid |
| MCF.7 | LINC00313 | hsa-miR-650 | 1560 | 1580 | -34.19 | miRanda,TargetScan,RNAhybrid |
| MCF.7 | LINC01126 | hsa-miR-663a | 459 | 481 | -37.78 | miRanda,TargetScan,RNAhybrid |
| MCF.7 | LAMTOR5-AS1 | hsa-miR-761 | 242 | 263 | -32.26 | miRanda,TargetScan,RNAhybrid |
| MCF.7 | LINC01526 | hsa-miR-761 | 81 | 103 | -27.14 | miRanda,TargetScan,RNAhybrid |
| MCF.7 | LINC01526 | hsa-miR-761 | 204 | 226 | -27.14 | miRanda,TargetScan,RNAhybrid |
| MCF.7 | LINC01515 | hsa-miR-761 | 370 | 392 | -23.56 | miRanda,TargetScan,RNAhybrid |
| MCF.7 | LINC00339 | hsa-miR-765 | 135 | 155 | -32.07 | miRanda,TargetScan,RNAhybrid |
| MCF.7 | SPANXA2-OT1 | hsa-miR-940 | 359 | 379 | -32.32 | miRanda,TargetScan,RNAhybrid |
| MCF.7 | A1BG-AS1 | hsa-miR-1204 | 67 | 89 | -32.56 | miRanda,TargetScan,RNAhybrid |
| MCF.7 | A1BG-AS1 | hsa-miR-1204 | 81 | 101 | -23.54 | miRanda,TargetScan,RNAhybrid |
| MCF.7 | LINC01118 | hsa-miR-1303 | 77 | 98 | -37.75 | miRanda,TargetScan,RNAhybrid |
| MCF.7 | LINC01118 | hsa-miR-1303 | 60 | 81 | -37.75 | miRanda,TargetScan,RNAhybrid |
| MCF.7 | LINC00324 | hsa-miR-1260b | 931 | 950 | -31.77 | miRanda,TargetScan,RNAhybrid |
| MCF.7 | SNHG4 | hsa-miR-3170 | 252 | 273 | -27.25 | miRanda,TargetScan,RNAhybrid |
| MCF.7 | GATA2-AS1 | hsa-miR-3176 | 61 | 79 | -27.53 | miRanda,TargetScan,RNAhybrid |
| MCF.7 | GATA2-AS1 | hsa-miR-3176 | 48 | 66 | -27.53 | miRanda,TargetScan,RNAhybrid |
| MCF.7 | SNHG12 | hsa-miR-3178 | 297 | 313 | -31.38 | miRanda,TargetScan,RNAhybrid |
| MCF.7 | SNHG12 | hsa-miR-3178 | 441 | 457 | -31.38 | miRanda,TargetScan,RNAhybrid |
| MCF.7 | SNHG12 | hsa-miR-3178 | 544 | 560 | -31.38 | miRanda,TargetScan,RNAhybrid |
| MCF.7 | SNHG12 | hsa-miR-3178 | 612 | 628 | -31.38 | miRanda,TargetScan,RNAhybrid |
| MCF.7 | SNHG12 | hsa-miR-3178 | 724 | 740 | -31.38 | miRanda,TargetScan,RNAhybrid |
| MCF.7 | SNHG12 | hsa-miR-3178 | 932 | 948 | -31.38 | miRanda,TargetScan,RNAhybrid |
| MCF.7 | SNHG12 | hsa-miR-3178 | 1255 | 1271 | -31.38 | miRanda,TargetScan,RNAhybrid |
| MCF.7 | SNHG12 | hsa-miR-3178 | 213 | 229 | -31.38 | miRanda,TargetScan,RNAhybrid |
| MCF.7 | SNHG12 | hsa-miR-3178 | 47 | 63 | -31.38 | miRanda,TargetScan,RNAhybrid |
| MCF.7 | SNHG12 | hsa-miR-3178 | 186 | 202 | -31.38 | miRanda,TargetScan,RNAhybrid |
| MCF.7 | SNHG12 | hsa-miR-3178 | 194 | 210 | -31.38 | miRanda,TargetScan,RNAhybrid |
| MCF.7 | SNHG12 | hsa-miR-3178 | 205 | 221 | -31.38 | miRanda,TargetScan,RNAhybrid |
| MCF.7 | SNHG12 | hsa-miR-3178 | 206 | 222 | -31.38 | miRanda,TargetScan,RNAhybrid |
| MCF.7 | SNHG12 | hsa-miR-3178 | 209 | 225 | -31.38 | miRanda,TargetScan,RNAhybrid |
| MCF.7 | SNHG12 | hsa-miR-3178 | 224 | 240 | -31.38 | miRanda,TargetScan,RNAhybrid |
| MCF.7 | SNHG12 | hsa-miR-3178 | 225 | 241 | -31.38 | miRanda,TargetScan,RNAhybrid |
| MCF.7 | SNHG12 | hsa-miR-3178 | 230 | 246 | -31.38 | miRanda,TargetScan,RNAhybrid |
| MCF.7 | SNHG12 | hsa-miR-3178 | 15 | 31 | -31.38 | miRanda,TargetScan,RNAhybrid |
| MCF.7 | SNHG12 | hsa-miR-3178 | 170 | 186 | -31.38 | miRanda,TargetScan,RNAhybrid |
| MCF.7 | SNHG12 | hsa-miR-3178 | 149 | 165 | -31.38 | miRanda,TargetScan,RNAhybrid |
| MCF.7 | SNHG12 | hsa-miR-3178 | 219 | 235 | -31.38 | miRanda,TargetScan,RNAhybrid |
| MCF.7 | SNHG12 | hsa-miR-3178 | 223 | 239 | -31.38 | miRanda,TargetScan,RNAhybrid |
| MCF.7 | AP4B1-AS1 | hsa-miR-3178 | 131 | 147 | -30.94 | miRanda,TargetScan,RNAhybrid |
| MCF.7 | CAHM | hsa-miR-3178 | 35 | 53 | -32.32 | miRanda,TargetScan,RNAhybrid |
| MCF.7 | NEBL-AS1 | hsa-miR-3178 | 29 | 45 | -32.38 | miRanda,TargetScan,RNAhybrid |
| MCF.7 | ZEB1-AS1 | hsa-miR-3178 | 260 | 276 | -31.96 | miRanda,TargetScan,RNAhybrid |
| MCF.7 | LINC00637 | hsa-miR-3178 | 456 | 472 | -32.07 | miRanda,TargetScan,RNAhybrid |
| MCF.7 | MMP25-AS1 | hsa-miR-3178 | 867 | 883 | -36.22 | miRanda,TargetScan,RNAhybrid |
| MCF.7 | MMP25-AS1 | hsa-miR-3178 | 840 | 856 | -36.22 | miRanda,TargetScan,RNAhybrid |
| MCF.7 | LINC00910 | hsa-miR-3178 | 517 | 533 | -28.76 | miRanda,TargetScan,RNAhybrid |
| MCF.7 | LINC00910 | hsa-miR-3178 | 542 | 558 | -28.76 | miRanda,TargetScan,RNAhybrid |
| MCF.7 | LINC00910 | hsa-miR-3178 | 857 | 873 | -28.76 | miRanda,TargetScan,RNAhybrid |
| MCF.7 | LINC00910 | hsa-miR-3178 | 648 | 664 | -28.76 | miRanda,TargetScan,RNAhybrid |
| MCF.7 | LINC00910 | hsa-miR-3178 | 577 | 593 | -28.76 | miRanda,TargetScan,RNAhybrid |
| MCF.7 | LINC00910 | hsa-miR-3178 | 506 | 522 | -28.76 | miRanda,TargetScan,RNAhybrid |
| MCF.7 | LINC00910 | hsa-miR-3178 | 529 | 545 | -28.76 | miRanda,TargetScan,RNAhybrid |
| MCF.7 | LINC00910 | hsa-miR-3178 | 257 | 273 | -28.76 | miRanda,TargetScan,RNAhybrid |
| MCF.7 | ASB16-AS1 | hsa-miR-3178 | 851 | 867 | -32.15 | miRanda,TargetScan,RNAhybrid |
| MCF.7 | PAX8-AS1 | hsa-miR-320e | 15 | 32 | -25.81 | miRanda,TargetScan,RNAhybrid |
| MCF.7 | ZMIZ1-AS1 | hsa-miR-3195 | 601 | 617 | -35.09 | miRanda,TargetScan,RNAhybrid |
| MCF.7 | NR2F2-AS1 | hsa-miR-3195 | 76 | 92 | -28.24 | miRanda,TargetScan,RNAhybrid |
| MCF.7 | NR2F2-AS1 | hsa-miR-3195 | 26 | 42 | -28.24 | miRanda,TargetScan,RNAhybrid |
| MCF.7 | NR2F2-AS1 | hsa-miR-3195 | 74 | 90 | -28.24 | miRanda,TargetScan,RNAhybrid |
| MCF.7 | NR2F2-AS1 | hsa-miR-3195 | 55 | 71 | -28.24 | miRanda,TargetScan,RNAhybrid |
| MCF.7 | NR2F2-AS1 | hsa-miR-3195 | 87 | 103 | -28.24 | miRanda,TargetScan,RNAhybrid |
| MCF.7 | SNAI3-AS1 | hsa-miR-3195 | 75 | 91 | -31.24 | miRanda,TargetScan,RNAhybrid |
| MCF.7 | GATA6-AS1 | hsa-miR-3195 | 119 | 136 | -35.28 | miRanda,TargetScan,RNAhybrid |
| MCF.7 | LINC01135 | hsa-miR-3197 | 134 | 157 | -34.05 | miRanda,TargetScan,RNAhybrid |
| MCF.7 | LINC01535 | hsa-miR-3197 | 484 | 506 | -33.16 | miRanda,TargetScan,RNAhybrid |
| MCF.7 | DIO3OS | hsa-miR-3198 | 71 | 92 | -37.9 | miRanda,TargetScan,RNAhybrid |
| MCF.7 | DIO3OS | hsa-miR-3198 | 152 | 173 | -37.9 | miRanda,TargetScan,RNAhybrid |
| MCF.7 | DIO3OS | hsa-miR-3198 | 270 | 291 | -37.9 | miRanda,TargetScan,RNAhybrid |
| MCF.7 | DIO3OS | hsa-miR-3198 | 281 | 302 | -37.9 | miRanda,TargetScan,RNAhybrid |
| MCF.7 | DIO3OS | hsa-miR-3198 | 636 | 657 | -37.9 | miRanda,TargetScan,RNAhybrid |
| MCF.7 | DIO3OS | hsa-miR-3198 | 722 | 743 | -37.9 | miRanda,TargetScan,RNAhybrid |
| MCF.7 | HOXA11-AS | hsa-miR-4286 | 208 | 224 | -25.92 | miRanda,TargetScan,RNAhybrid |
| MCF.7 | HOTAIR | hsa-miR-4286 | 275 | 291 | -22.5 | miRanda,TargetScan,RNAhybrid |
| MCF.7 | MMP25-AS1 | hsa-miR-4286 | 190 | 206 | -26.51 | miRanda,TargetScan,RNAhybrid |
| MCF.7 | LINC01239 | hsa-miR-331-3p | 603 | 623 | -29.48 | miRanda,TargetScan,RNAhybrid |
| MCF.7 | LINC01239 | hsa-miR-331-3p | 184 | 204 | -29.48 | miRanda,TargetScan,RNAhybrid |
| MCF.7 | LINC01239 | hsa-miR-331-3p | 126 | 146 | -29.48 | miRanda,TargetScan,RNAhybrid |
| MCF.7 | LINC01119 | hsa-miR-1303 | 77 | 98 | -37.75 | miRanda,TargetScan,RNAhybrid |
| MCF.7 | LINC01119 | hsa-miR-1303 | 60 | 81 | -37.75 | miRanda,TargetScan,RNAhybrid |
| NCI.H460 | LINC01270 | hsa-miR-326 | 60 | 79 | -34.19 | miRanda,TargetScan,RNAhybrid |
| NCI.H460 | LINC01270 | hsa-miR-326 | 49 | 68 | -34.19 | miRanda,TargetScan,RNAhybrid |
| NCI.H460 | LINC01270 | hsa-miR-326 | 20 | 39 | -34.19 | miRanda,TargetScan,RNAhybrid |
| NCI.H460 | MIR4435-2HG | hsa-miR-331-3p | 786 | 806 | -29.98 | miRanda,TargetScan,RNAhybrid |
| NCI.H460 | LINC00313 | hsa-miR-331-3p | 208 | 228 | -30.15 | miRanda,TargetScan,RNAhybrid |
| NCI.H460 | LINC00313 | hsa-miR-331-3p | 1138 | 1158 | -30.15 | miRanda,TargetScan,RNAhybrid |
| NCI.H460 | LINC00313 | hsa-miR-331-3p | 592 | 612 | -30.15 | miRanda,TargetScan,RNAhybrid |
| NCI.H460 | MAGI2-AS3 | hsa-miR-339-5p | 67 | 89 | -31.61 | miRanda,TargetScan,RNAhybrid |
| NCI.H460 | FEZF1-AS1 | hsa-miR-492 | 1360 | 1382 | -30.97 | miRanda,TargetScan,RNAhybrid |
| NCI.H460 | RAB11B-AS1 | hsa-miR-564 | 328 | 346 | -28.65 | miRanda,TargetScan,RNAhybrid |
| NCI.H460 | RAB11B-AS1 | hsa-miR-564 | 373 | 391 | -28.65 | miRanda,TargetScan,RNAhybrid |
| NCI.H460 | RAB11B-AS1 | hsa-miR-564 | 813 | 831 | -28.65 | miRanda,TargetScan,RNAhybrid |
| NCI.H460 | RAB11B-AS1 | hsa-miR-564 | 280 | 298 | -28.65 | miRanda,TargetScan,RNAhybrid |
| NCI.H460 | DGUOK-AS1 | hsa-miR-574-5p | 863 | 885 | -39.21 | miRanda,TargetScan,RNAhybrid |
| NCI.H460 | DGUOK-AS1 | hsa-miR-574-5p | 874 | 896 | -39.21 | miRanda,TargetScan,RNAhybrid |
| NCI.H460 | SNHG14 | hsa-miR-574-5p | 136 | 158 | -27.25 | miRanda,TargetScan,RNAhybrid |
| NCI.H460 | SNHG14 | hsa-miR-574-5p | 19 | 41 | -27.25 | miRanda,TargetScan,RNAhybrid |
| NCI.H460 | MIR222HG | hsa-miR-574-5p | 290 | 312 | -31.92 | miRanda,TargetScan,RNAhybrid |
| NCI.H460 | PTOV1-AS2 | hsa-miR-612 | 205 | 234 | -37.47 | miRanda,TargetScan,RNAhybrid |
| NCI.H460 | PTOV1-AS2 | hsa-miR-612 | 216 | 245 | -37.47 | miRanda,TargetScan,RNAhybrid |
| NCI.H460 | ENTPD3-AS1 | hsa-miR-632 | 122 | 140 | -24.91 | miRanda,TargetScan,RNAhybrid |
| NCI.H460 | LINC00313 | hsa-miR-650 | 1560 | 1580 | -34.19 | miRanda,TargetScan,RNAhybrid |
| NCI.H460 | LAMTOR5-AS1 | hsa-miR-761 | 242 | 263 | -32.26 | miRanda,TargetScan,RNAhybrid |
| NCI.H460 | LINC01515 | hsa-miR-761 | 370 | 392 | -23.56 | miRanda,TargetScan,RNAhybrid |
| NCI.H460 | LINC00339 | hsa-miR-765 | 135 | 155 | -32.07 | miRanda,TargetScan,RNAhybrid |
| NCI.H460 | A1BG-AS1 | hsa-miR-1204 | 67 | 89 | -32.56 | miRanda,TargetScan,RNAhybrid |
| NCI.H460 | A1BG-AS1 | hsa-miR-1204 | 81 | 101 | -23.54 | miRanda,TargetScan,RNAhybrid |
| NCI.H460 | SNHG4 | hsa-miR-3170 | 252 | 273 | -27.25 | miRanda,TargetScan,RNAhybrid |
| NCI.H460 | GATA2-AS1 | hsa-miR-3176 | 61 | 79 | -27.53 | miRanda,TargetScan,RNAhybrid |
| NCI.H460 | GATA2-AS1 | hsa-miR-3176 | 48 | 66 | -27.53 | miRanda,TargetScan,RNAhybrid |
| NCI.H460 | SNHG14 | hsa-miR-3176 | 313 | 331 | -32.49 | miRanda,TargetScan,RNAhybrid |
| NCI.H460 | SNHG14 | hsa-miR-3176 | 300 | 318 | -32.49 | miRanda,TargetScan,RNAhybrid |
| NCI.H460 | SNHG14 | hsa-miR-3176 | 30 | 48 | -26.12 | miRanda,TargetScan,RNAhybrid |
| NCI.H460 | SNHG12 | hsa-miR-3178 | 297 | 313 | -31.38 | miRanda,TargetScan,RNAhybrid |
| NCI.H460 | SNHG12 | hsa-miR-3178 | 441 | 457 | -31.38 | miRanda,TargetScan,RNAhybrid |
| NCI.H460 | SNHG12 | hsa-miR-3178 | 544 | 560 | -31.38 | miRanda,TargetScan,RNAhybrid |
| NCI.H460 | SNHG12 | hsa-miR-3178 | 612 | 628 | -31.38 | miRanda,TargetScan,RNAhybrid |
| NCI.H460 | SNHG12 | hsa-miR-3178 | 724 | 740 | -31.38 | miRanda,TargetScan,RNAhybrid |
| NCI.H460 | SNHG12 | hsa-miR-3178 | 932 | 948 | -31.38 | miRanda,TargetScan,RNAhybrid |
| NCI.H460 | SNHG12 | hsa-miR-3178 | 1255 | 1271 | -31.38 | miRanda,TargetScan,RNAhybrid |
| NCI.H460 | SNHG12 | hsa-miR-3178 | 213 | 229 | -31.38 | miRanda,TargetScan,RNAhybrid |
| NCI.H460 | SNHG12 | hsa-miR-3178 | 47 | 63 | -31.38 | miRanda,TargetScan,RNAhybrid |
| NCI.H460 | SNHG12 | hsa-miR-3178 | 186 | 202 | -31.38 | miRanda,TargetScan,RNAhybrid |
| NCI.H460 | SNHG12 | hsa-miR-3178 | 194 | 210 | -31.38 | miRanda,TargetScan,RNAhybrid |
| NCI.H460 | SNHG12 | hsa-miR-3178 | 205 | 221 | -31.38 | miRanda,TargetScan,RNAhybrid |
| NCI.H460 | SNHG12 | hsa-miR-3178 | 206 | 222 | -31.38 | miRanda,TargetScan,RNAhybrid |
| NCI.H460 | SNHG12 | hsa-miR-3178 | 209 | 225 | -31.38 | miRanda,TargetScan,RNAhybrid |
| NCI.H460 | SNHG12 | hsa-miR-3178 | 224 | 240 | -31.38 | miRanda,TargetScan,RNAhybrid |
| NCI.H460 | SNHG12 | hsa-miR-3178 | 225 | 241 | -31.38 | miRanda,TargetScan,RNAhybrid |
| NCI.H460 | SNHG12 | hsa-miR-3178 | 230 | 246 | -31.38 | miRanda,TargetScan,RNAhybrid |
| NCI.H460 | SNHG12 | hsa-miR-3178 | 15 | 31 | -31.38 | miRanda,TargetScan,RNAhybrid |
| NCI.H460 | SNHG12 | hsa-miR-3178 | 170 | 186 | -31.38 | miRanda,TargetScan,RNAhybrid |
| NCI.H460 | SNHG12 | hsa-miR-3178 | 149 | 165 | -31.38 | miRanda,TargetScan,RNAhybrid |
| NCI.H460 | SNHG12 | hsa-miR-3178 | 219 | 235 | -31.38 | miRanda,TargetScan,RNAhybrid |
| NCI.H460 | SNHG12 | hsa-miR-3178 | 223 | 239 | -31.38 | miRanda,TargetScan,RNAhybrid |
| NCI.H460 | CAHM | hsa-miR-3178 | 35 | 53 | -32.32 | miRanda,TargetScan,RNAhybrid |
| NCI.H460 | ZEB1-AS1 | hsa-miR-3178 | 260 | 276 | -31.96 | miRanda,TargetScan,RNAhybrid |
| NCI.H460 | MMP25-AS1 | hsa-miR-3178 | 867 | 883 | -36.22 | miRanda,TargetScan,RNAhybrid |
| NCI.H460 | MMP25-AS1 | hsa-miR-3178 | 840 | 856 | -36.22 | miRanda,TargetScan,RNAhybrid |
| NCI.H460 | LINC00910 | hsa-miR-3178 | 517 | 533 | -28.76 | miRanda,TargetScan,RNAhybrid |
| NCI.H460 | LINC00910 | hsa-miR-3178 | 542 | 558 | -28.76 | miRanda,TargetScan,RNAhybrid |
| NCI.H460 | LINC00910 | hsa-miR-3178 | 857 | 873 | -28.76 | miRanda,TargetScan,RNAhybrid |
| NCI.H460 | LINC00910 | hsa-miR-3178 | 648 | 664 | -28.76 | miRanda,TargetScan,RNAhybrid |
| NCI.H460 | LINC00910 | hsa-miR-3178 | 577 | 593 | -28.76 | miRanda,TargetScan,RNAhybrid |
| NCI.H460 | LINC00910 | hsa-miR-3178 | 506 | 522 | -28.76 | miRanda,TargetScan,RNAhybrid |
| NCI.H460 | LINC00910 | hsa-miR-3178 | 529 | 545 | -28.76 | miRanda,TargetScan,RNAhybrid |
| NCI.H460 | LINC00910 | hsa-miR-3178 | 257 | 273 | -28.76 | miRanda,TargetScan,RNAhybrid |
| NCI.H460 | ASB16-AS1 | hsa-miR-3178 | 851 | 867 | -32.15 | miRanda,TargetScan,RNAhybrid |
| NCI.H460 | LINC00899 | hsa-miR-3178 | 25 | 41 | -36.65 | miRanda,TargetScan,RNAhybrid |
| NCI.H460 | LINC00899 | hsa-miR-3178 | 10 | 26 | -36.65 | miRanda,TargetScan,RNAhybrid |
| NCI.H460 | LINC00899 | hsa-miR-3178 | 88 | 104 | -36.65 | miRanda,TargetScan,RNAhybrid |
| NCI.H460 | LINC00899 | hsa-miR-3178 | 53 | 69 | -36.65 | miRanda,TargetScan,RNAhybrid |
| NCI.H460 | PXN-AS1 | hsa-miR-3183 | 1013 | 1034 | -30.77 | miRanda,TargetScan,RNAhybrid |
| NCI.H460 | PXN-AS1 | hsa-miR-3183 | 1127 | 1148 | -30.77 | miRanda,TargetScan,RNAhybrid |
| NCI.H460 | PXN-AS1 | hsa-miR-3183 | 1110 | 1131 | -30.77 | miRanda,TargetScan,RNAhybrid |
| NCI.H460 | PXN-AS1 | hsa-miR-3183 | 1014 | 1035 | -30.77 | miRanda,TargetScan,RNAhybrid |
| NCI.H460 | PAX8-AS1 | hsa-miR-320e | 15 | 32 | -25.81 | miRanda,TargetScan,RNAhybrid |
| NCI.H460 | HOXA-AS2 | hsa-miR-3195 | 476 | 492 | -30.42 | miRanda,TargetScan,RNAhybrid |
| NCI.H460 | NR2F2-AS1 | hsa-miR-3195 | 76 | 92 | -28.24 | miRanda,TargetScan,RNAhybrid |
| NCI.H460 | NR2F2-AS1 | hsa-miR-3195 | 26 | 42 | -28.24 | miRanda,TargetScan,RNAhybrid |
| NCI.H460 | NR2F2-AS1 | hsa-miR-3195 | 74 | 90 | -28.24 | miRanda,TargetScan,RNAhybrid |
| NCI.H460 | NR2F2-AS1 | hsa-miR-3195 | 55 | 71 | -28.24 | miRanda,TargetScan,RNAhybrid |
| NCI.H460 | NR2F2-AS1 | hsa-miR-3195 | 87 | 103 | -28.24 | miRanda,TargetScan,RNAhybrid |
| NCI.H460 | SNAI3-AS1 | hsa-miR-3195 | 75 | 91 | -31.24 | miRanda,TargetScan,RNAhybrid |
| NCI.H460 | LINC00894 | hsa-miR-3195 | 908 | 924 | -33.3 | miRanda,TargetScan,RNAhybrid |
| NCI.H460 | DNAH17-AS1 | hsa-miR-3197 | 182 | 204 | -33.96 | miRanda,TargetScan,RNAhybrid |
| NCI.H460 | DNAH17-AS1 | hsa-miR-3197 | 169 | 191 | -33.96 | miRanda,TargetScan,RNAhybrid |
| NCI.H460 | DNAH17-AS1 | hsa-miR-3197 | 140 | 162 | -33.96 | miRanda,TargetScan,RNAhybrid |
| NCI.H460 | HOXA11-AS | hsa-miR-4286 | 208 | 224 | -25.92 | miRanda,TargetScan,RNAhybrid |
| NCI.H460 | MMP25-AS1 | hsa-miR-4286 | 190 | 206 | -26.51 | miRanda,TargetScan,RNAhybrid |
| NCI.H460 | RAB11B-AS1 | hsa-miR-4286 | 196 | 212 | -29.7 | miRanda,TargetScan,RNAhybrid |
| NHEK | LINC01270 | hsa-miR-326 | 60 | 79 | -34.19 | miRanda,TargetScan,RNAhybrid |
| NHEK | LINC01270 | hsa-miR-326 | 49 | 68 | -34.19 | miRanda,TargetScan,RNAhybrid |
| NHEK | LINC01270 | hsa-miR-326 | 20 | 39 | -34.19 | miRanda,TargetScan,RNAhybrid |
| NHEK | CDKN2B-AS1 | hsa-miR-331-3p | 603 | 623 | -29.48 | miRanda,TargetScan,RNAhybrid |
| NHEK | CDKN2B-AS1 | hsa-miR-331-3p | 184 | 204 | -29.48 | miRanda,TargetScan,RNAhybrid |
| NHEK | CDKN2B-AS1 | hsa-miR-331-3p | 126 | 146 | -29.48 | miRanda,TargetScan,RNAhybrid |
| NHEK | CASC2 | hsa-miR-331-3p | 42 | 61 | -30.94 | miRanda,TargetScan,RNAhybrid |
| NHEK | LINC00511 | hsa-miR-331-3p | 201 | 220 | -27.07 | miRanda,TargetScan,RNAhybrid |
| NHEK | LINC00511 | hsa-miR-331-3p | 334 | 353 | -27.07 | miRanda,TargetScan,RNAhybrid |
| NHEK | LINC00511 | hsa-miR-331-3p | 122 | 141 | -27.07 | miRanda,TargetScan,RNAhybrid |
| NHEK | LINC00511 | hsa-miR-331-3p | 473 | 492 | -27.07 | miRanda,TargetScan,RNAhybrid |
| NHEK | LINC00511 | hsa-miR-331-3p | 595 | 614 | -27.07 | miRanda,TargetScan,RNAhybrid |
| NHEK | SNHG14 | hsa-miR-574-5p | 136 | 158 | -27.25 | miRanda,TargetScan,RNAhybrid |
| NHEK | SNHG14 | hsa-miR-574-5p | 19 | 41 | -27.25 | miRanda,TargetScan,RNAhybrid |
| NHEK | PTOV1-AS2 | hsa-miR-612 | 205 | 234 | -37.47 | miRanda,TargetScan,RNAhybrid |
| NHEK | PTOV1-AS2 | hsa-miR-612 | 216 | 245 | -37.47 | miRanda,TargetScan,RNAhybrid |
| NHEK | LINC01515 | hsa-miR-761 | 370 | 392 | -23.56 | miRanda,TargetScan,RNAhybrid |
| NHEK | A1BG-AS1 | hsa-miR-1204 | 67 | 89 | -32.56 | miRanda,TargetScan,RNAhybrid |
| NHEK | A1BG-AS1 | hsa-miR-1204 | 81 | 101 | -23.54 | miRanda,TargetScan,RNAhybrid |
| NHEK | SNHG4 | hsa-miR-3170 | 252 | 273 | -27.25 | miRanda,TargetScan,RNAhybrid |
| NHEK | SNHG14 | hsa-miR-3176 | 313 | 331 | -32.49 | miRanda,TargetScan,RNAhybrid |
| NHEK | SNHG14 | hsa-miR-3176 | 300 | 318 | -32.49 | miRanda,TargetScan,RNAhybrid |
| NHEK | SNHG14 | hsa-miR-3176 | 30 | 48 | -26.12 | miRanda,TargetScan,RNAhybrid |
| NHEK | SNHG12 | hsa-miR-3178 | 297 | 313 | -31.38 | miRanda,TargetScan,RNAhybrid |
| NHEK | SNHG12 | hsa-miR-3178 | 441 | 457 | -31.38 | miRanda,TargetScan,RNAhybrid |
| NHEK | SNHG12 | hsa-miR-3178 | 544 | 560 | -31.38 | miRanda,TargetScan,RNAhybrid |
| NHEK | SNHG12 | hsa-miR-3178 | 612 | 628 | -31.38 | miRanda,TargetScan,RNAhybrid |
| NHEK | SNHG12 | hsa-miR-3178 | 724 | 740 | -31.38 | miRanda,TargetScan,RNAhybrid |
| NHEK | SNHG12 | hsa-miR-3178 | 932 | 948 | -31.38 | miRanda,TargetScan,RNAhybrid |
| NHEK | SNHG12 | hsa-miR-3178 | 1255 | 1271 | -31.38 | miRanda,TargetScan,RNAhybrid |
| NHEK | SNHG12 | hsa-miR-3178 | 213 | 229 | -31.38 | miRanda,TargetScan,RNAhybrid |
| NHEK | SNHG12 | hsa-miR-3178 | 47 | 63 | -31.38 | miRanda,TargetScan,RNAhybrid |
| NHEK | SNHG12 | hsa-miR-3178 | 186 | 202 | -31.38 | miRanda,TargetScan,RNAhybrid |
| NHEK | SNHG12 | hsa-miR-3178 | 194 | 210 | -31.38 | miRanda,TargetScan,RNAhybrid |
| NHEK | SNHG12 | hsa-miR-3178 | 205 | 221 | -31.38 | miRanda,TargetScan,RNAhybrid |
| NHEK | SNHG12 | hsa-miR-3178 | 206 | 222 | -31.38 | miRanda,TargetScan,RNAhybrid |
| NHEK | SNHG12 | hsa-miR-3178 | 209 | 225 | -31.38 | miRanda,TargetScan,RNAhybrid |
| NHEK | SNHG12 | hsa-miR-3178 | 224 | 240 | -31.38 | miRanda,TargetScan,RNAhybrid |
| NHEK | SNHG12 | hsa-miR-3178 | 225 | 241 | -31.38 | miRanda,TargetScan,RNAhybrid |
| NHEK | SNHG12 | hsa-miR-3178 | 230 | 246 | -31.38 | miRanda,TargetScan,RNAhybrid |
| NHEK | SNHG12 | hsa-miR-3178 | 15 | 31 | -31.38 | miRanda,TargetScan,RNAhybrid |
| NHEK | SNHG12 | hsa-miR-3178 | 170 | 186 | -31.38 | miRanda,TargetScan,RNAhybrid |
| NHEK | SNHG12 | hsa-miR-3178 | 149 | 165 | -31.38 | miRanda,TargetScan,RNAhybrid |
| NHEK | SNHG12 | hsa-miR-3178 | 219 | 235 | -31.38 | miRanda,TargetScan,RNAhybrid |
| NHEK | SNHG12 | hsa-miR-3178 | 223 | 239 | -31.38 | miRanda,TargetScan,RNAhybrid |
| NHEK | ZEB1-AS1 | hsa-miR-3178 | 260 | 276 | -31.96 | miRanda,TargetScan,RNAhybrid |
| NHEK | MMP25-AS1 | hsa-miR-3178 | 867 | 883 | -36.22 | miRanda,TargetScan,RNAhybrid |
| NHEK | MMP25-AS1 | hsa-miR-3178 | 840 | 856 | -36.22 | miRanda,TargetScan,RNAhybrid |
| NHEK | LINC00910 | hsa-miR-3178 | 517 | 533 | -28.76 | miRanda,TargetScan,RNAhybrid |
| NHEK | LINC00910 | hsa-miR-3178 | 542 | 558 | -28.76 | miRanda,TargetScan,RNAhybrid |
| NHEK | LINC00910 | hsa-miR-3178 | 857 | 873 | -28.76 | miRanda,TargetScan,RNAhybrid |
| NHEK | LINC00910 | hsa-miR-3178 | 648 | 664 | -28.76 | miRanda,TargetScan,RNAhybrid |
| NHEK | LINC00910 | hsa-miR-3178 | 577 | 593 | -28.76 | miRanda,TargetScan,RNAhybrid |
| NHEK | LINC00910 | hsa-miR-3178 | 506 | 522 | -28.76 | miRanda,TargetScan,RNAhybrid |
| NHEK | LINC00910 | hsa-miR-3178 | 529 | 545 | -28.76 | miRanda,TargetScan,RNAhybrid |
| NHEK | LINC00910 | hsa-miR-3178 | 257 | 273 | -28.76 | miRanda,TargetScan,RNAhybrid |
| NHEK | ASB16-AS1 | hsa-miR-3178 | 851 | 867 | -32.15 | miRanda,TargetScan,RNAhybrid |
| NHEK | ZNF582-AS1 | hsa-miR-3178 | 155 | 171 | -33.98 | miRanda,TargetScan,RNAhybrid |
| NHEK | LINC00899 | hsa-miR-3178 | 25 | 41 | -36.65 | miRanda,TargetScan,RNAhybrid |
| NHEK | LINC00899 | hsa-miR-3178 | 10 | 26 | -36.65 | miRanda,TargetScan,RNAhybrid |
| NHEK | LINC00899 | hsa-miR-3178 | 88 | 104 | -36.65 | miRanda,TargetScan,RNAhybrid |
| NHEK | LINC00899 | hsa-miR-3178 | 53 | 69 | -36.65 | miRanda,TargetScan,RNAhybrid |
| NHEK | PAX8-AS1 | hsa-miR-320e | 15 | 32 | -25.81 | miRanda,TargetScan,RNAhybrid |
| NHEK | HOXA-AS2 | hsa-miR-3195 | 476 | 492 | -30.42 | miRanda,TargetScan,RNAhybrid |
| NHEK | SNAI3-AS1 | hsa-miR-3195 | 75 | 91 | -31.24 | miRanda,TargetScan,RNAhybrid |
| NHEK | HOTAIR | hsa-miR-4286 | 275 | 291 | -22.5 | miRanda,TargetScan,RNAhybrid |
| NHEK | MMP25-AS1 | hsa-miR-4286 | 190 | 206 | -26.51 | miRanda,TargetScan,RNAhybrid |
| NHEK | LINC00511 | hsa-miR-4286 | 282 | 298 | -32.18 | miRanda,TargetScan,RNAhybrid |
| NHEK | LINC00511 | hsa-miR-4286 | 679 | 695 | -32.18 | miRanda,TargetScan,RNAhybrid |
| NHEK | LINC01239 | hsa-miR-331-3p | 603 | 623 | -29.48 | miRanda,TargetScan,RNAhybrid |
| NHEK | LINC01239 | hsa-miR-331-3p | 184 | 204 | -29.48 | miRanda,TargetScan,RNAhybrid |
| NHEK | LINC01239 | hsa-miR-331-3p | 126 | 146 | -29.48 | miRanda,TargetScan,RNAhybrid |
| NHEK | PWAR5 | hsa-miR-574-5p | 136 | 158 | -27.25 | miRanda,TargetScan,RNAhybrid |
| NHEK | PWAR5 | hsa-miR-574-5p | 19 | 41 | -27.25 | miRanda,TargetScan,RNAhybrid |
| NHEK | FAM66B | hsa-miR-612 | 146 | 170 | -33.24 | miRanda,TargetScan,RNAhybrid |
| NHEK | PWAR5 | hsa-miR-3176 | 313 | 331 | -32.49 | miRanda,TargetScan,RNAhybrid |
| NHEK | PWAR5 | hsa-miR-3176 | 300 | 318 | -32.49 | miRanda,TargetScan,RNAhybrid |
| NHEK | PWAR5 | hsa-miR-3176 | 30 | 48 | -26.12 | miRanda,TargetScan,RNAhybrid |
| SK.MEL.5 | LINC01270 | hsa-miR-326 | 60 | 79 | -34.19 | miRanda,TargetScan,RNAhybrid |
| SK.MEL.5 | LINC01270 | hsa-miR-326 | 49 | 68 | -34.19 | miRanda,TargetScan,RNAhybrid |
| SK.MEL.5 | LINC01270 | hsa-miR-326 | 20 | 39 | -34.19 | miRanda,TargetScan,RNAhybrid |
| SK.MEL.5 | CASC2 | hsa-miR-331-3p | 42 | 61 | -30.94 | miRanda,TargetScan,RNAhybrid |
| SK.MEL.5 | FEZF1-AS1 | hsa-miR-492 | 1360 | 1382 | -30.97 | miRanda,TargetScan,RNAhybrid |
| SK.MEL.5 | SNHG14 | hsa-miR-574-5p | 136 | 158 | -27.25 | miRanda,TargetScan,RNAhybrid |
| SK.MEL.5 | SNHG14 | hsa-miR-574-5p | 19 | 41 | -27.25 | miRanda,TargetScan,RNAhybrid |
| SK.MEL.5 | LINC01515 | hsa-miR-761 | 370 | 392 | -23.56 | miRanda,TargetScan,RNAhybrid |
| SK.MEL.5 | LINC00339 | hsa-miR-765 | 135 | 155 | -32.07 | miRanda,TargetScan,RNAhybrid |
| SK.MEL.5 | SPANXA2-OT1 | hsa-miR-940 | 359 | 379 | -32.32 | miRanda,TargetScan,RNAhybrid |
| SK.MEL.5 | SNHG4 | hsa-miR-3170 | 252 | 273 | -27.25 | miRanda,TargetScan,RNAhybrid |
| SK.MEL.5 | SNHG14 | hsa-miR-3176 | 313 | 331 | -32.49 | miRanda,TargetScan,RNAhybrid |
| SK.MEL.5 | SNHG14 | hsa-miR-3176 | 300 | 318 | -32.49 | miRanda,TargetScan,RNAhybrid |
| SK.MEL.5 | SNHG14 | hsa-miR-3176 | 30 | 48 | -26.12 | miRanda,TargetScan,RNAhybrid |
| SK.MEL.5 | SNHG12 | hsa-miR-3178 | 297 | 313 | -31.38 | miRanda,TargetScan,RNAhybrid |
| SK.MEL.5 | SNHG12 | hsa-miR-3178 | 441 | 457 | -31.38 | miRanda,TargetScan,RNAhybrid |
| SK.MEL.5 | SNHG12 | hsa-miR-3178 | 544 | 560 | -31.38 | miRanda,TargetScan,RNAhybrid |
| SK.MEL.5 | SNHG12 | hsa-miR-3178 | 612 | 628 | -31.38 | miRanda,TargetScan,RNAhybrid |
| SK.MEL.5 | SNHG12 | hsa-miR-3178 | 724 | 740 | -31.38 | miRanda,TargetScan,RNAhybrid |
| SK.MEL.5 | SNHG12 | hsa-miR-3178 | 932 | 948 | -31.38 | miRanda,TargetScan,RNAhybrid |
| SK.MEL.5 | SNHG12 | hsa-miR-3178 | 1255 | 1271 | -31.38 | miRanda,TargetScan,RNAhybrid |
| SK.MEL.5 | SNHG12 | hsa-miR-3178 | 213 | 229 | -31.38 | miRanda,TargetScan,RNAhybrid |
| SK.MEL.5 | SNHG12 | hsa-miR-3178 | 47 | 63 | -31.38 | miRanda,TargetScan,RNAhybrid |
| SK.MEL.5 | SNHG12 | hsa-miR-3178 | 186 | 202 | -31.38 | miRanda,TargetScan,RNAhybrid |
| SK.MEL.5 | SNHG12 | hsa-miR-3178 | 194 | 210 | -31.38 | miRanda,TargetScan,RNAhybrid |
| SK.MEL.5 | SNHG12 | hsa-miR-3178 | 205 | 221 | -31.38 | miRanda,TargetScan,RNAhybrid |
| SK.MEL.5 | SNHG12 | hsa-miR-3178 | 206 | 222 | -31.38 | miRanda,TargetScan,RNAhybrid |
| SK.MEL.5 | SNHG12 | hsa-miR-3178 | 209 | 225 | -31.38 | miRanda,TargetScan,RNAhybrid |
| SK.MEL.5 | SNHG12 | hsa-miR-3178 | 224 | 240 | -31.38 | miRanda,TargetScan,RNAhybrid |
| SK.MEL.5 | SNHG12 | hsa-miR-3178 | 225 | 241 | -31.38 | miRanda,TargetScan,RNAhybrid |
| SK.MEL.5 | SNHG12 | hsa-miR-3178 | 230 | 246 | -31.38 | miRanda,TargetScan,RNAhybrid |
| SK.MEL.5 | SNHG12 | hsa-miR-3178 | 15 | 31 | -31.38 | miRanda,TargetScan,RNAhybrid |
| SK.MEL.5 | SNHG12 | hsa-miR-3178 | 170 | 186 | -31.38 | miRanda,TargetScan,RNAhybrid |
| SK.MEL.5 | SNHG12 | hsa-miR-3178 | 149 | 165 | -31.38 | miRanda,TargetScan,RNAhybrid |
| SK.MEL.5 | SNHG12 | hsa-miR-3178 | 219 | 235 | -31.38 | miRanda,TargetScan,RNAhybrid |
| SK.MEL.5 | SNHG12 | hsa-miR-3178 | 223 | 239 | -31.38 | miRanda,TargetScan,RNAhybrid |
| SK.MEL.5 | MMP25-AS1 | hsa-miR-3178 | 867 | 883 | -36.22 | miRanda,TargetScan,RNAhybrid |
| SK.MEL.5 | MMP25-AS1 | hsa-miR-3178 | 840 | 856 | -36.22 | miRanda,TargetScan,RNAhybrid |
| SK.MEL.5 | LINC00910 | hsa-miR-3178 | 517 | 533 | -28.76 | miRanda,TargetScan,RNAhybrid |
| SK.MEL.5 | LINC00910 | hsa-miR-3178 | 542 | 558 | -28.76 | miRanda,TargetScan,RNAhybrid |
| SK.MEL.5 | LINC00910 | hsa-miR-3178 | 857 | 873 | -28.76 | miRanda,TargetScan,RNAhybrid |
| SK.MEL.5 | LINC00910 | hsa-miR-3178 | 648 | 664 | -28.76 | miRanda,TargetScan,RNAhybrid |
| SK.MEL.5 | LINC00910 | hsa-miR-3178 | 577 | 593 | -28.76 | miRanda,TargetScan,RNAhybrid |
| SK.MEL.5 | LINC00910 | hsa-miR-3178 | 506 | 522 | -28.76 | miRanda,TargetScan,RNAhybrid |
| SK.MEL.5 | LINC00910 | hsa-miR-3178 | 529 | 545 | -28.76 | miRanda,TargetScan,RNAhybrid |
| SK.MEL.5 | LINC00910 | hsa-miR-3178 | 257 | 273 | -28.76 | miRanda,TargetScan,RNAhybrid |
| SK.MEL.5 | ASB16-AS1 | hsa-miR-3178 | 851 | 867 | -32.15 | miRanda,TargetScan,RNAhybrid |
| SK.MEL.5 | HAS2-AS1 | hsa-miR-3182 | 113 | 129 | -22.1 | miRanda,TargetScan,RNAhybrid |
| SK.MEL.5 | HAS2-AS1 | hsa-miR-3182 | 111 | 127 | -22.1 | miRanda,TargetScan,RNAhybrid |
| SK.MEL.5 | PAX8-AS1 | hsa-miR-320e | 15 | 32 | -25.81 | miRanda,TargetScan,RNAhybrid |
| SK.MEL.5 | MAPKAPK5-AS1 | hsa-miR-3197 | 553 | 575 | -36.14 | miRanda,TargetScan,RNAhybrid |
| SK.MEL.5 | MMP25-AS1 | hsa-miR-4286 | 190 | 206 | -26.51 | miRanda,TargetScan,RNAhybrid |
| SK.MEL.5 | PWAR5 | hsa-miR-574-5p | 136 | 158 | -27.25 | miRanda,TargetScan,RNAhybrid |
| SK.MEL.5 | PWAR5 | hsa-miR-574-5p | 19 | 41 | -27.25 | miRanda,TargetScan,RNAhybrid |
| SK.MEL.5 | PWAR5 | hsa-miR-3176 | 313 | 331 | -32.49 | miRanda,TargetScan,RNAhybrid |
| SK.MEL.5 | PWAR5 | hsa-miR-3176 | 300 | 318 | -32.49 | miRanda,TargetScan,RNAhybrid |
| SK.MEL.5 | PWAR5 | hsa-miR-3176 | 30 | 48 | -26.12 | miRanda,TargetScan,RNAhybrid |
| SK.N.DZ | LINC01270 | hsa-miR-326 | 60 | 79 | -34.19 | miRanda,TargetScan,RNAhybrid |
| SK.N.DZ | LINC01270 | hsa-miR-326 | 49 | 68 | -34.19 | miRanda,TargetScan,RNAhybrid |
| SK.N.DZ | LINC01270 | hsa-miR-326 | 20 | 39 | -34.19 | miRanda,TargetScan,RNAhybrid |
| SK.N.DZ | MEG3 | hsa-miR-331-3p | 235 | 255 | -31.44 | miRanda,TargetScan,RNAhybrid |
| SK.N.DZ | MAGI2-AS3 | hsa-miR-339-5p | 67 | 89 | -31.61 | miRanda,TargetScan,RNAhybrid |
| SK.N.DZ | RAB11B-AS1 | hsa-miR-564 | 328 | 346 | -28.65 | miRanda,TargetScan,RNAhybrid |
| SK.N.DZ | RAB11B-AS1 | hsa-miR-564 | 373 | 391 | -28.65 | miRanda,TargetScan,RNAhybrid |
| SK.N.DZ | RAB11B-AS1 | hsa-miR-564 | 813 | 831 | -28.65 | miRanda,TargetScan,RNAhybrid |
| SK.N.DZ | RAB11B-AS1 | hsa-miR-564 | 280 | 298 | -28.65 | miRanda,TargetScan,RNAhybrid |
| SK.N.DZ | HOXD-AS2 | hsa-miR-574-5p | 284 | 306 | -17.66 | miRanda,TargetScan,RNAhybrid |
| SK.N.DZ | HOXD-AS2 | hsa-miR-574-5p | 859 | 881 | -17.66 | miRanda,TargetScan,RNAhybrid |
| SK.N.DZ | SNHG14 | hsa-miR-574-5p | 136 | 158 | -27.25 | miRanda,TargetScan,RNAhybrid |
| SK.N.DZ | SNHG14 | hsa-miR-574-5p | 19 | 41 | -27.25 | miRanda,TargetScan,RNAhybrid |
| SK.N.DZ | LINC01126 | hsa-miR-663a | 459 | 481 | -37.78 | miRanda,TargetScan,RNAhybrid |
| SK.N.DZ | GACAT3 | hsa-miR-657 | 191 | 213 | -31.17 | miRanda,TargetScan,RNAhybrid |
| SK.N.DZ | LAMTOR5-AS1 | hsa-miR-761 | 242 | 263 | -32.26 | miRanda,TargetScan,RNAhybrid |
| SK.N.DZ | LINC01515 | hsa-miR-761 | 370 | 392 | -23.56 | miRanda,TargetScan,RNAhybrid |
| SK.N.DZ | LINC01122 | hsa-miR-892b | 811 | 832 | -34.95 | miRanda,TargetScan,RNAhybrid |
| SK.N.DZ | LINC01122 | hsa-miR-892b | 651 | 672 | -34.95 | miRanda,TargetScan,RNAhybrid |
| SK.N.DZ | LINC01122 | hsa-miR-892b | 798 | 819 | -34.95 | miRanda,TargetScan,RNAhybrid |
| SK.N.DZ | LINC01122 | hsa-miR-892b | 542 | 563 | -34.95 | miRanda,TargetScan,RNAhybrid |
| SK.N.DZ | LINC01122 | hsa-miR-892b | 645 | 666 | -34.95 | miRanda,TargetScan,RNAhybrid |
| SK.N.DZ | LINC01122 | hsa-miR-892b | 695 | 716 | -34.95 | miRanda,TargetScan,RNAhybrid |
| SK.N.DZ | LINC01122 | hsa-miR-892b | 958 | 979 | -34.95 | miRanda,TargetScan,RNAhybrid |
| SK.N.DZ | LINC01122 | hsa-miR-892b | 577 | 598 | -34.95 | miRanda,TargetScan,RNAhybrid |
| SK.N.DZ | LINC01122 | hsa-miR-892b | 686 | 707 | -34.95 | miRanda,TargetScan,RNAhybrid |
| SK.N.DZ | LINC01122 | hsa-miR-892b | 733 | 754 | -34.95 | miRanda,TargetScan,RNAhybrid |
| SK.N.DZ | LINC01122 | hsa-miR-892b | 673 | 694 | -34.95 | miRanda,TargetScan,RNAhybrid |
| SK.N.DZ | LINC01122 | hsa-miR-892b | 556 | 577 | -34.95 | miRanda,TargetScan,RNAhybrid |
| SK.N.DZ | LINC01122 | hsa-miR-892b | 205 | 226 | -34.95 | miRanda,TargetScan,RNAhybrid |
| SK.N.DZ | FGF14-AS2 | hsa-miR-760 | 342 | 361 | -31.57 | miRanda,TargetScan,RNAhybrid |
| SK.N.DZ | FGF14-AS2 | hsa-miR-760 | 293 | 312 | -31.57 | miRanda,TargetScan,RNAhybrid |
| SK.N.DZ | FGF14-AS2 | hsa-miR-760 | 290 | 309 | -31.57 | miRanda,TargetScan,RNAhybrid |
| SK.N.DZ | LINC01250 | hsa-miR-3170 | 594 | 615 | -41.84 | miRanda,TargetScan,RNAhybrid |
| SK.N.DZ | LINC01250 | hsa-miR-3170 | 571 | 592 | -41.84 | miRanda,TargetScan,RNAhybrid |
| SK.N.DZ | SNHG4 | hsa-miR-3170 | 252 | 273 | -27.25 | miRanda,TargetScan,RNAhybrid |
| SK.N.DZ | GATA2-AS1 | hsa-miR-3176 | 61 | 79 | -27.53 | miRanda,TargetScan,RNAhybrid |
| SK.N.DZ | GATA2-AS1 | hsa-miR-3176 | 48 | 66 | -27.53 | miRanda,TargetScan,RNAhybrid |
| SK.N.DZ | SNHG14 | hsa-miR-3176 | 313 | 331 | -32.49 | miRanda,TargetScan,RNAhybrid |
| SK.N.DZ | SNHG14 | hsa-miR-3176 | 300 | 318 | -32.49 | miRanda,TargetScan,RNAhybrid |
| SK.N.DZ | SNHG14 | hsa-miR-3176 | 30 | 48 | -26.12 | miRanda,TargetScan,RNAhybrid |
| SK.N.DZ | SNHG12 | hsa-miR-3178 | 297 | 313 | -31.38 | miRanda,TargetScan,RNAhybrid |
| SK.N.DZ | SNHG12 | hsa-miR-3178 | 441 | 457 | -31.38 | miRanda,TargetScan,RNAhybrid |
| SK.N.DZ | SNHG12 | hsa-miR-3178 | 544 | 560 | -31.38 | miRanda,TargetScan,RNAhybrid |
| SK.N.DZ | SNHG12 | hsa-miR-3178 | 612 | 628 | -31.38 | miRanda,TargetScan,RNAhybrid |
| SK.N.DZ | SNHG12 | hsa-miR-3178 | 724 | 740 | -31.38 | miRanda,TargetScan,RNAhybrid |
| SK.N.DZ | SNHG12 | hsa-miR-3178 | 932 | 948 | -31.38 | miRanda,TargetScan,RNAhybrid |
| SK.N.DZ | SNHG12 | hsa-miR-3178 | 1255 | 1271 | -31.38 | miRanda,TargetScan,RNAhybrid |
| SK.N.DZ | SNHG12 | hsa-miR-3178 | 213 | 229 | -31.38 | miRanda,TargetScan,RNAhybrid |
| SK.N.DZ | SNHG12 | hsa-miR-3178 | 47 | 63 | -31.38 | miRanda,TargetScan,RNAhybrid |
| SK.N.DZ | SNHG12 | hsa-miR-3178 | 186 | 202 | -31.38 | miRanda,TargetScan,RNAhybrid |
| SK.N.DZ | SNHG12 | hsa-miR-3178 | 194 | 210 | -31.38 | miRanda,TargetScan,RNAhybrid |
| SK.N.DZ | SNHG12 | hsa-miR-3178 | 205 | 221 | -31.38 | miRanda,TargetScan,RNAhybrid |
| SK.N.DZ | SNHG12 | hsa-miR-3178 | 206 | 222 | -31.38 | miRanda,TargetScan,RNAhybrid |
| SK.N.DZ | SNHG12 | hsa-miR-3178 | 209 | 225 | -31.38 | miRanda,TargetScan,RNAhybrid |
| SK.N.DZ | SNHG12 | hsa-miR-3178 | 224 | 240 | -31.38 | miRanda,TargetScan,RNAhybrid |
| SK.N.DZ | SNHG12 | hsa-miR-3178 | 225 | 241 | -31.38 | miRanda,TargetScan,RNAhybrid |
| SK.N.DZ | SNHG12 | hsa-miR-3178 | 230 | 246 | -31.38 | miRanda,TargetScan,RNAhybrid |
| SK.N.DZ | SNHG12 | hsa-miR-3178 | 15 | 31 | -31.38 | miRanda,TargetScan,RNAhybrid |
| SK.N.DZ | SNHG12 | hsa-miR-3178 | 170 | 186 | -31.38 | miRanda,TargetScan,RNAhybrid |
| SK.N.DZ | SNHG12 | hsa-miR-3178 | 149 | 165 | -31.38 | miRanda,TargetScan,RNAhybrid |
| SK.N.DZ | SNHG12 | hsa-miR-3178 | 219 | 235 | -31.38 | miRanda,TargetScan,RNAhybrid |
| SK.N.DZ | SNHG12 | hsa-miR-3178 | 223 | 239 | -31.38 | miRanda,TargetScan,RNAhybrid |
| SK.N.DZ | CAHM | hsa-miR-3178 | 35 | 53 | -32.32 | miRanda,TargetScan,RNAhybrid |
| SK.N.DZ | MMP25-AS1 | hsa-miR-3178 | 867 | 883 | -36.22 | miRanda,TargetScan,RNAhybrid |
| SK.N.DZ | MMP25-AS1 | hsa-miR-3178 | 840 | 856 | -36.22 | miRanda,TargetScan,RNAhybrid |
| SK.N.DZ | ASB16-AS1 | hsa-miR-3178 | 851 | 867 | -32.15 | miRanda,TargetScan,RNAhybrid |
| SK.N.DZ | ZNF582-AS1 | hsa-miR-3178 | 155 | 171 | -33.98 | miRanda,TargetScan,RNAhybrid |
| SK.N.DZ | LINC00894 | hsa-miR-3195 | 908 | 924 | -33.3 | miRanda,TargetScan,RNAhybrid |
| SK.N.DZ | MMP25-AS1 | hsa-miR-4286 | 190 | 206 | -26.51 | miRanda,TargetScan,RNAhybrid |
| SK.N.DZ | RAB11B-AS1 | hsa-miR-4286 | 196 | 212 | -29.7 | miRanda,TargetScan,RNAhybrid |
| SK.N.DZ | MEG8 | hsa-miR-331-3p | 235 | 255 | -31.44 | miRanda,TargetScan,RNAhybrid |
| SK.N.SH | LINC01600 | hsa-miR-326 | 685 | 704 | -34.52 | miRanda,TargetScan,RNAhybrid |
| SK.N.SH | LINC01600 | hsa-miR-326 | 622 | 641 | -34.52 | miRanda,TargetScan,RNAhybrid |
| SK.N.SH | KRTAP5-AS1 | hsa-miR-326 | 532 | 551 | -37.75 | miRanda,TargetScan,RNAhybrid |
| SK.N.SH | KRTAP5-AS1 | hsa-miR-326 | 391 | 410 | -37.75 | miRanda,TargetScan,RNAhybrid |
| SK.N.SH | LINC01270 | hsa-miR-326 | 60 | 79 | -34.19 | miRanda,TargetScan,RNAhybrid |
| SK.N.SH | LINC01270 | hsa-miR-326 | 49 | 68 | -34.19 | miRanda,TargetScan,RNAhybrid |
| SK.N.SH | LINC01270 | hsa-miR-326 | 20 | 39 | -34.19 | miRanda,TargetScan,RNAhybrid |
| SK.N.SH | DARS-AS1 | hsa-miR-331-3p | 199 | 219 | -25.99 | miRanda,TargetScan,RNAhybrid |
| SK.N.SH | DARS-AS1 | hsa-miR-331-3p | 172 | 192 | -25.99 | miRanda,TargetScan,RNAhybrid |
| SK.N.SH | DARS-AS1 | hsa-miR-331-3p | 171 | 191 | -25.99 | miRanda,TargetScan,RNAhybrid |
| SK.N.SH | DARS-AS1 | hsa-miR-331-3p | 155 | 175 | -25.99 | miRanda,TargetScan,RNAhybrid |
| SK.N.SH | DARS-AS1 | hsa-miR-331-3p | 129 | 149 | -25.99 | miRanda,TargetScan,RNAhybrid |
| SK.N.SH | DARS-AS1 | hsa-miR-331-3p | 14 | 34 | -25.99 | miRanda,TargetScan,RNAhybrid |
| SK.N.SH | FAM85B | hsa-miR-331-3p | 59 | 79 | -33.94 | miRanda,TargetScan,RNAhybrid |
| SK.N.SH | FAM85B | hsa-miR-331-3p | 10 | 30 | -33.94 | miRanda,TargetScan,RNAhybrid |
| SK.N.SH | LINC00310 | hsa-miR-331-3p | 429 | 449 | -30.57 | miRanda,TargetScan,RNAhybrid |
| SK.N.SH | LINC00310 | hsa-miR-331-3p | 808 | 828 | -30.57 | miRanda,TargetScan,RNAhybrid |
| SK.N.SH | LINC00310 | hsa-miR-331-3p | 788 | 808 | -30.57 | miRanda,TargetScan,RNAhybrid |
| SK.N.SH | LINC00310 | hsa-miR-331-3p | 792 | 812 | -30.57 | miRanda,TargetScan,RNAhybrid |
| SK.N.SH | LINC00313 | hsa-miR-331-3p | 208 | 228 | -30.15 | miRanda,TargetScan,RNAhybrid |
| SK.N.SH | LINC00313 | hsa-miR-331-3p | 1138 | 1158 | -30.15 | miRanda,TargetScan,RNAhybrid |
| SK.N.SH | LINC00313 | hsa-miR-331-3p | 592 | 612 | -30.15 | miRanda,TargetScan,RNAhybrid |
| SK.N.SH | PCAT14 | hsa-miR-331-3p | 772 | 792 | -31.91 | miRanda,TargetScan,RNAhybrid |
| SK.N.SH | MAGI2-AS3 | hsa-miR-339-5p | 67 | 89 | -31.61 | miRanda,TargetScan,RNAhybrid |
| SK.N.SH | SNHG14 | hsa-miR-574-5p | 136 | 158 | -27.25 | miRanda,TargetScan,RNAhybrid |
| SK.N.SH | SNHG14 | hsa-miR-574-5p | 19 | 41 | -27.25 | miRanda,TargetScan,RNAhybrid |
| SK.N.SH | MIR222HG | hsa-miR-574-5p | 290 | 312 | -31.92 | miRanda,TargetScan,RNAhybrid |
| SK.N.SH | PTOV1-AS2 | hsa-miR-612 | 205 | 234 | -37.47 | miRanda,TargetScan,RNAhybrid |
| SK.N.SH | PTOV1-AS2 | hsa-miR-612 | 216 | 245 | -37.47 | miRanda,TargetScan,RNAhybrid |
| SK.N.SH | SEMA3F-AS1 | hsa-miR-650 | 102 | 122 | -27.92 | miRanda,TargetScan,RNAhybrid |
| SK.N.SH | LINC00313 | hsa-miR-650 | 1560 | 1580 | -34.19 | miRanda,TargetScan,RNAhybrid |
| SK.N.SH | LAMTOR5-AS1 | hsa-miR-761 | 242 | 263 | -32.26 | miRanda,TargetScan,RNAhybrid |
| SK.N.SH | A1BG-AS1 | hsa-miR-1204 | 67 | 89 | -32.56 | miRanda,TargetScan,RNAhybrid |
| SK.N.SH | A1BG-AS1 | hsa-miR-1204 | 81 | 101 | -23.54 | miRanda,TargetScan,RNAhybrid |
| SK.N.SH | LINC01118 | hsa-miR-1303 | 77 | 98 | -37.75 | miRanda,TargetScan,RNAhybrid |
| SK.N.SH | LINC01118 | hsa-miR-1303 | 60 | 81 | -37.75 | miRanda,TargetScan,RNAhybrid |
| SK.N.SH | LINC01250 | hsa-miR-3170 | 594 | 615 | -41.84 | miRanda,TargetScan,RNAhybrid |
| SK.N.SH | LINC01250 | hsa-miR-3170 | 571 | 592 | -41.84 | miRanda,TargetScan,RNAhybrid |
| SK.N.SH | SNHG4 | hsa-miR-3170 | 252 | 273 | -27.25 | miRanda,TargetScan,RNAhybrid |
| SK.N.SH | GATA2-AS1 | hsa-miR-3176 | 61 | 79 | -27.53 | miRanda,TargetScan,RNAhybrid |
| SK.N.SH | GATA2-AS1 | hsa-miR-3176 | 48 | 66 | -27.53 | miRanda,TargetScan,RNAhybrid |
| SK.N.SH | SNHG14 | hsa-miR-3176 | 313 | 331 | -32.49 | miRanda,TargetScan,RNAhybrid |
| SK.N.SH | SNHG14 | hsa-miR-3176 | 300 | 318 | -32.49 | miRanda,TargetScan,RNAhybrid |
| SK.N.SH | SNHG14 | hsa-miR-3176 | 30 | 48 | -26.12 | miRanda,TargetScan,RNAhybrid |
| SK.N.SH | SNHG12 | hsa-miR-3178 | 297 | 313 | -31.38 | miRanda,TargetScan,RNAhybrid |
| SK.N.SH | SNHG12 | hsa-miR-3178 | 441 | 457 | -31.38 | miRanda,TargetScan,RNAhybrid |
| SK.N.SH | SNHG12 | hsa-miR-3178 | 544 | 560 | -31.38 | miRanda,TargetScan,RNAhybrid |
| SK.N.SH | SNHG12 | hsa-miR-3178 | 612 | 628 | -31.38 | miRanda,TargetScan,RNAhybrid |
| SK.N.SH | SNHG12 | hsa-miR-3178 | 724 | 740 | -31.38 | miRanda,TargetScan,RNAhybrid |
| SK.N.SH | SNHG12 | hsa-miR-3178 | 932 | 948 | -31.38 | miRanda,TargetScan,RNAhybrid |
| SK.N.SH | SNHG12 | hsa-miR-3178 | 1255 | 1271 | -31.38 | miRanda,TargetScan,RNAhybrid |
| SK.N.SH | SNHG12 | hsa-miR-3178 | 213 | 229 | -31.38 | miRanda,TargetScan,RNAhybrid |
| SK.N.SH | SNHG12 | hsa-miR-3178 | 47 | 63 | -31.38 | miRanda,TargetScan,RNAhybrid |
| SK.N.SH | SNHG12 | hsa-miR-3178 | 186 | 202 | -31.38 | miRanda,TargetScan,RNAhybrid |
| SK.N.SH | SNHG12 | hsa-miR-3178 | 194 | 210 | -31.38 | miRanda,TargetScan,RNAhybrid |
| SK.N.SH | SNHG12 | hsa-miR-3178 | 205 | 221 | -31.38 | miRanda,TargetScan,RNAhybrid |
| SK.N.SH | SNHG12 | hsa-miR-3178 | 206 | 222 | -31.38 | miRanda,TargetScan,RNAhybrid |
| SK.N.SH | SNHG12 | hsa-miR-3178 | 209 | 225 | -31.38 | miRanda,TargetScan,RNAhybrid |
| SK.N.SH | SNHG12 | hsa-miR-3178 | 224 | 240 | -31.38 | miRanda,TargetScan,RNAhybrid |
| SK.N.SH | SNHG12 | hsa-miR-3178 | 225 | 241 | -31.38 | miRanda,TargetScan,RNAhybrid |
| SK.N.SH | SNHG12 | hsa-miR-3178 | 230 | 246 | -31.38 | miRanda,TargetScan,RNAhybrid |
| SK.N.SH | SNHG12 | hsa-miR-3178 | 15 | 31 | -31.38 | miRanda,TargetScan,RNAhybrid |
| SK.N.SH | SNHG12 | hsa-miR-3178 | 170 | 186 | -31.38 | miRanda,TargetScan,RNAhybrid |
| SK.N.SH | SNHG12 | hsa-miR-3178 | 149 | 165 | -31.38 | miRanda,TargetScan,RNAhybrid |
| SK.N.SH | SNHG12 | hsa-miR-3178 | 219 | 235 | -31.38 | miRanda,TargetScan,RNAhybrid |
| SK.N.SH | SNHG12 | hsa-miR-3178 | 223 | 239 | -31.38 | miRanda,TargetScan,RNAhybrid |
| SK.N.SH | CAHM | hsa-miR-3178 | 35 | 53 | -32.32 | miRanda,TargetScan,RNAhybrid |
| SK.N.SH | MMP25-AS1 | hsa-miR-3178 | 867 | 883 | -36.22 | miRanda,TargetScan,RNAhybrid |
| SK.N.SH | MMP25-AS1 | hsa-miR-3178 | 840 | 856 | -36.22 | miRanda,TargetScan,RNAhybrid |
| SK.N.SH | LINC00910 | hsa-miR-3178 | 517 | 533 | -28.76 | miRanda,TargetScan,RNAhybrid |
| SK.N.SH | LINC00910 | hsa-miR-3178 | 542 | 558 | -28.76 | miRanda,TargetScan,RNAhybrid |
| SK.N.SH | LINC00910 | hsa-miR-3178 | 857 | 873 | -28.76 | miRanda,TargetScan,RNAhybrid |
| SK.N.SH | LINC00910 | hsa-miR-3178 | 648 | 664 | -28.76 | miRanda,TargetScan,RNAhybrid |
| SK.N.SH | LINC00910 | hsa-miR-3178 | 577 | 593 | -28.76 | miRanda,TargetScan,RNAhybrid |
| SK.N.SH | LINC00910 | hsa-miR-3178 | 506 | 522 | -28.76 | miRanda,TargetScan,RNAhybrid |
| SK.N.SH | LINC00910 | hsa-miR-3178 | 529 | 545 | -28.76 | miRanda,TargetScan,RNAhybrid |
| SK.N.SH | LINC00910 | hsa-miR-3178 | 257 | 273 | -28.76 | miRanda,TargetScan,RNAhybrid |
| SK.N.SH | ASB16-AS1 | hsa-miR-3178 | 851 | 867 | -32.15 | miRanda,TargetScan,RNAhybrid |
| SK.N.SH | ZNF582-AS1 | hsa-miR-3178 | 155 | 171 | -33.98 | miRanda,TargetScan,RNAhybrid |
| SK.N.SH | PXN-AS1 | hsa-miR-3183 | 1013 | 1034 | -30.77 | miRanda,TargetScan,RNAhybrid |
| SK.N.SH | PXN-AS1 | hsa-miR-3183 | 1127 | 1148 | -30.77 | miRanda,TargetScan,RNAhybrid |
| SK.N.SH | PXN-AS1 | hsa-miR-3183 | 1110 | 1131 | -30.77 | miRanda,TargetScan,RNAhybrid |
| SK.N.SH | PXN-AS1 | hsa-miR-3183 | 1014 | 1035 | -30.77 | miRanda,TargetScan,RNAhybrid |
| SK.N.SH | ZMIZ1-AS1 | hsa-miR-3195 | 601 | 617 | -35.09 | miRanda,TargetScan,RNAhybrid |
| SK.N.SH | NR2F2-AS1 | hsa-miR-3195 | 76 | 92 | -28.24 | miRanda,TargetScan,RNAhybrid |
| SK.N.SH | NR2F2-AS1 | hsa-miR-3195 | 26 | 42 | -28.24 | miRanda,TargetScan,RNAhybrid |
| SK.N.SH | NR2F2-AS1 | hsa-miR-3195 | 74 | 90 | -28.24 | miRanda,TargetScan,RNAhybrid |
| SK.N.SH | NR2F2-AS1 | hsa-miR-3195 | 55 | 71 | -28.24 | miRanda,TargetScan,RNAhybrid |
| SK.N.SH | NR2F2-AS1 | hsa-miR-3195 | 87 | 103 | -28.24 | miRanda,TargetScan,RNAhybrid |
| SK.N.SH | SNAI3-AS1 | hsa-miR-3195 | 75 | 91 | -31.24 | miRanda,TargetScan,RNAhybrid |
| SK.N.SH | LINC01535 | hsa-miR-3197 | 484 | 506 | -33.16 | miRanda,TargetScan,RNAhybrid |
| SK.N.SH | POT1-AS1 | hsa-miR-4286 | 897 | 913 | -26.71 | miRanda,TargetScan,RNAhybrid |
| SK.N.SH | MMP25-AS1 | hsa-miR-4286 | 190 | 206 | -26.51 | miRanda,TargetScan,RNAhybrid |
| SK.N.SH | LINC01239 | hsa-miR-331-3p | 603 | 623 | -29.48 | miRanda,TargetScan,RNAhybrid |
| SK.N.SH | LINC01239 | hsa-miR-331-3p | 184 | 204 | -29.48 | miRanda,TargetScan,RNAhybrid |
| SK.N.SH | LINC01239 | hsa-miR-331-3p | 126 | 146 | -29.48 | miRanda,TargetScan,RNAhybrid |
| SK.N.SH | PWAR5 | hsa-miR-574-5p | 136 | 158 | -27.25 | miRanda,TargetScan,RNAhybrid |
| SK.N.SH | PWAR5 | hsa-miR-574-5p | 19 | 41 | -27.25 | miRanda,TargetScan,RNAhybrid |
| SK.N.SH | PWAR5 | hsa-miR-3176 | 313 | 331 | -32.49 | miRanda,TargetScan,RNAhybrid |
| SK.N.SH | PWAR5 | hsa-miR-3176 | 300 | 318 | -32.49 | miRanda,TargetScan,RNAhybrid |
| SK.N.SH | PWAR5 | hsa-miR-3176 | 30 | 48 | -26.12 | miRanda,TargetScan,RNAhybrid |

**Table S6 Primers designed for reverse-transcription, qPCR, ChIP and plasmid construction.**

| Genes | Primer sequences (5’ to 3’) | Usage |
| --- | --- | --- |
| ssc-miR-375 | CTCAACTGGTGTCGTGGAGTCGGCAATTCAGTTGAGTCACGCGA | Reverse-transcription |
| ssc-miR-378 | CTCAACTGGTGTCGTGGAGTCGGCAATTCAGTTGAGGCCTTCTG | Reverse-transcription |
| ssc-miR-144 | CTCAACTGGTGTCGTGGAGTCGGCAATTCAGTTGAGGTACATC | Reverse-transcription |
| ssc-miR-339 | CTCAACTGGTGTCGTGGAGTCGGCAATTCAGTTGAGGTGAGCTC | Reverse-transcription |
| hsa-miR-24 | CTCAACTGGTGTCGTGGAGTCGGCAATTCAGTTGAGCTGTGTTT | Reverse-transcription |
| hsa-miR-29b | CTCAACTGGTGTCGTGGAGTCGGCAATTCAGTTGAGTCTAAACCA | Reverse-transcription |
| hsa-miR-26 | CTCAACTGGTGTCGTGGAGTCGGCAATTCAGTTGAGTGCCTATC | Reverse-transcription |
| hsa-miR-373 | CTCAACTGGTGTCGTGGAGTCGGCAATTCAGTTGAGGGAAAGCG | Reverse-transcription |
| ssc-miR-375 | F: GCCGAGTTTGTTCGTTCGGC R: CTCAACTGGTGTCGTGGA | qPCR |
| ssc-miR-378 | F: GCCGAGACTGGACTTGGAGT R: CTCAACTGGTGTCGTGGA | qPCR |
| ssc-miR-144 | F: GCCGAGTACAGTATAGAT R: CTCAACTGGTGTCGTGGA | qPCR |
| ssc-miR-339 | F: GCCGAGTCCCTGTCCTCCAG R: CTCAACTGGTGTCGTGGA | qPCR |
| hsa-miR-24 | F: GCCGAGTGCCTACTGAGCTG R: CTCAACTGGTGTCGTGGA | qPCR |
| hsa-miR-29b | F: GCCGAGGCTGGTTTCATATGG R: CTCAACTGGTGTCGTGGA | qPCR |
| hsa-miR-26 | F: GCCGAGTTCAAGTAATCCAG R: CTCAACTGGTGTCGTGGA | qPCR |
| hsa-miR-373 | F: GCCGAGACTCAAAATGGGGG R: CTCAACTGGTGTCGTGGA | qPCR |
| LOC102167708 | F: GTCCCCTCCTCTTGTGGTCAT R: ACGTGTGATGAAGTGTTTCAAACC | qPCR |
| LOC102160522 | F: GCAGACTGTTGCTTTGGGAG R: CCTTCATCTGGCTCAGTGGC | qPCR |
| LOC100512907 | F: TGTGCTTCTCTAGGACTGATTGT R: GGTATCCCGCACCTCTATCTA | qPCR |
| LOC100626841 | F: AGGGGAAGCAGGACCAGAGA R: CGGGCTAAGAGAAGAAGGCA | qPCR |
| NORSF | F: ACAGGGAAGCGAGTGGATGA R: CGCCTCGGCTTCCTACTAAAT | qPCR |
| CYP19A1 | F: GCTGCTCATTGGCTTAC R: TCCACCTATCCAGACCC | qPCR |
| MALAT1 | F: AAAGCAAGGTCTCCCCACAAG R: GGTCTGTGCTAGATCAAAAGGCA | qPCR |
| NEAT1 | F: GGTGGCAGTGCTCCTTTTGG R: CACCATTACCAACAATACCGACTC | qPCR |
| U6 for pig | F: CGCTTCGGCAGCACATATAC R: TTCACGAATTTGCGTGTCAT | qPCR |
| GAPDH for pig | F: GGACTCATGACCACGGTCCAT R: TCAGATCCACAACCGACACGT | qPCR |
| U6 for human | F: CTCGCTTCGGCAGCACATATACT R: ATTTGCGTGTCATCCTTGCGCA | qPCR |
| GAPDH for human | F: GAACGGGAAGCTCACTGG R: GCCTGCTTCACCACCTTCT | qPCR |
| CYP19A1 | F: AAGGAGTTAGCTGCTGGGGG R: GAGAAAGAGAAGTAATGGGGGC | ChIP |
| NORSF | F: CCCTCGAGGCTTATCGCTAAGCATTGTTCT R: TGCTCTAGA CAAGAAAGCTCATAATGTTTCCA | Overexpression vector construction |

**Table S7 Oligonucleotide sequences used in this study**

| Name | Sequence (5’ to 3’) |
| --- | --- |
| mimics NC | UUGUACUACACAAAAGUACUG |
| miR-339 mimics | UCCCUGUCCUCCAGGAGCUCAC |
| NC-siRNA | Sense:UUCUCCGAACGUGUCACGUTT  Anti-Sense:ACGUGACACGUUCGGAGAATT |
| NORSF-siRNA | Sense:GUCAGAAUGUGCAGUGACUTT  Anti-Sense:AGUCACUGCACAUUCUGACTT |
| NC-siRNA | Sense:UUCUCCGAACGUGUCACGUTT  Anti-Sense:ACGUGACACGUUCGGAGAATT |
| CYP19A1-siRNA | Sense:GCAGUGCCUGCAACUACUATT  Anti-Sense:UAGUAGUUGCAGGCACUGCTT |

**Table S8 Biotin-labeled antisense probes.**

| Genes | Antisense probe sequence (5’ to 3’) |
| --- | --- |
| LOC102160522 | ACTTTTGAAGCACGATCCTT-Biotin |
| LOC100626841 | GAAGAAGGCAGTTGGGATCT-Biotin |
| LOC100512907 | GTCTCAAAGTCATCTGGGAT-Biotin |
| NORSF | CATGGTAGAGAATCCCATCA-Biotin |
| MALAT1 | CTTGGAAAACGCCTCAATCC-Biotin |
| NEAT1 | GAACTTCTCCGAGAAACGCA-Biotin |
