# Supplementary material for: Nuclear lncRNA NORSF reduces E2 release in granulosa cells by sponging the endogenous small activating RNA miR-339
Source: BMC Biol. 2023 Oct 20;21:221. doi: 10.1186/s12915-023-01731-x (PMC10588145; doi:10.1186/s12915-023-01731-x)
Supplement: Supplementary file 2 — Additional file 2: Fig. S1. The RNA expression profile of five DELs in sow follicular theca cells and GCs. Fig. S2. Heatmap of miR-144, miR-378, miR-339 and miR-375 signals in sow follicles and GCs. Fig. S3. Other two potential MREs of miR-339 in LOC102164325. Fig. S4. Identification of lncRNA NORSF. Fig. S5. NORSF negatively regulates CYP19A1 expression in GCs. Fig. S6. Percentage of lncRNA nucleoplasm in multiple human cell types. Fig. S7. Interaction networks of lncRNAs and miRNAs in the nucleus of multiple human cell types. Fig. S8. Nuclear lncRNAs MALAT1 and NEAT1 interact with multiple miRNAs in the nucleus of human GCs. Fig. S9. miR-339 mature sequences are highly conserved among vertebrates. Fig. S10. The MRE motifs of miR-339 in human LOC124906953. [file 12915_2023_1731_MOESM2_ESM.docx]

**Supporting information**


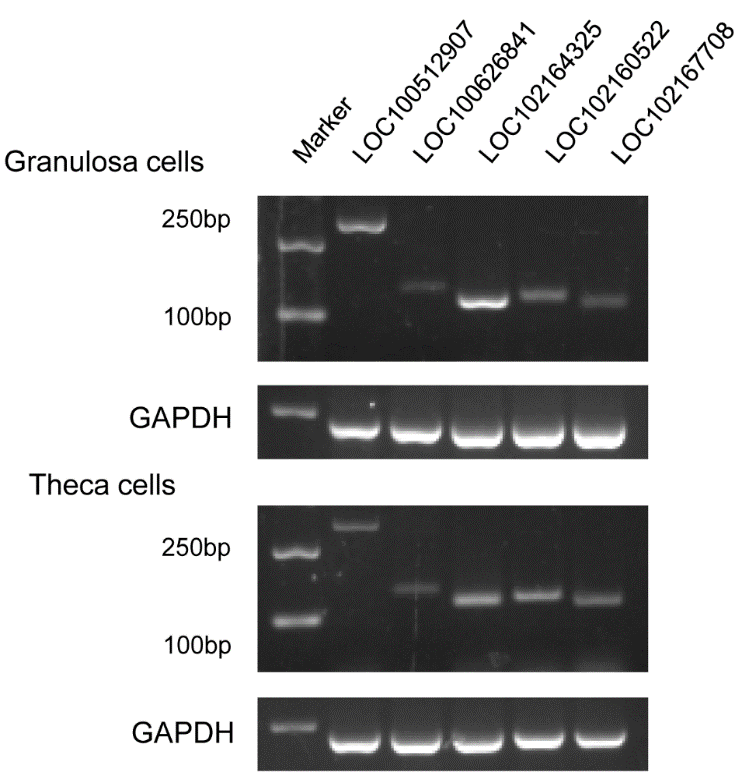


**Fig S1. The RNA expression profile of five DELs in sow follicular theca cells and GCs.**

**
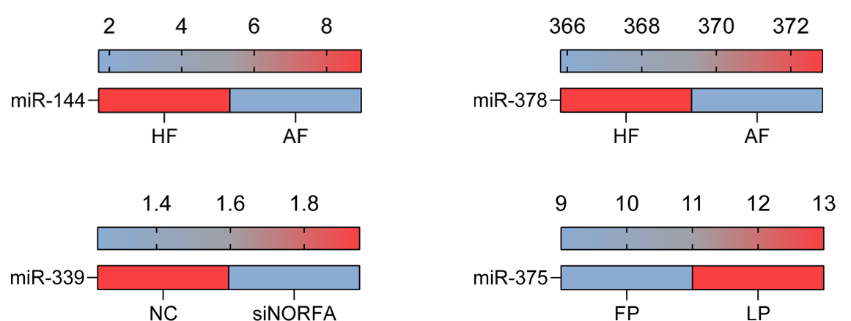
**

**Fig S2. Heatmap of miR-144, miR-378, miR-339 and miR-375 signals in sow follicles and GCs.**

miR-144 and miR-378 were differentially expressed in healthy follicle (HF) and early atretic follicle (AF) by using RNA-seq and microarray. miR-339 was differentially expressed in GCs treated with or without a siRNA for NORFA, an anti-apoptotic lncRNA. miR-375 was differentially expressed in ovaries of the follicular phase (FP) and luteal phase (LP) by using RNA-seq.


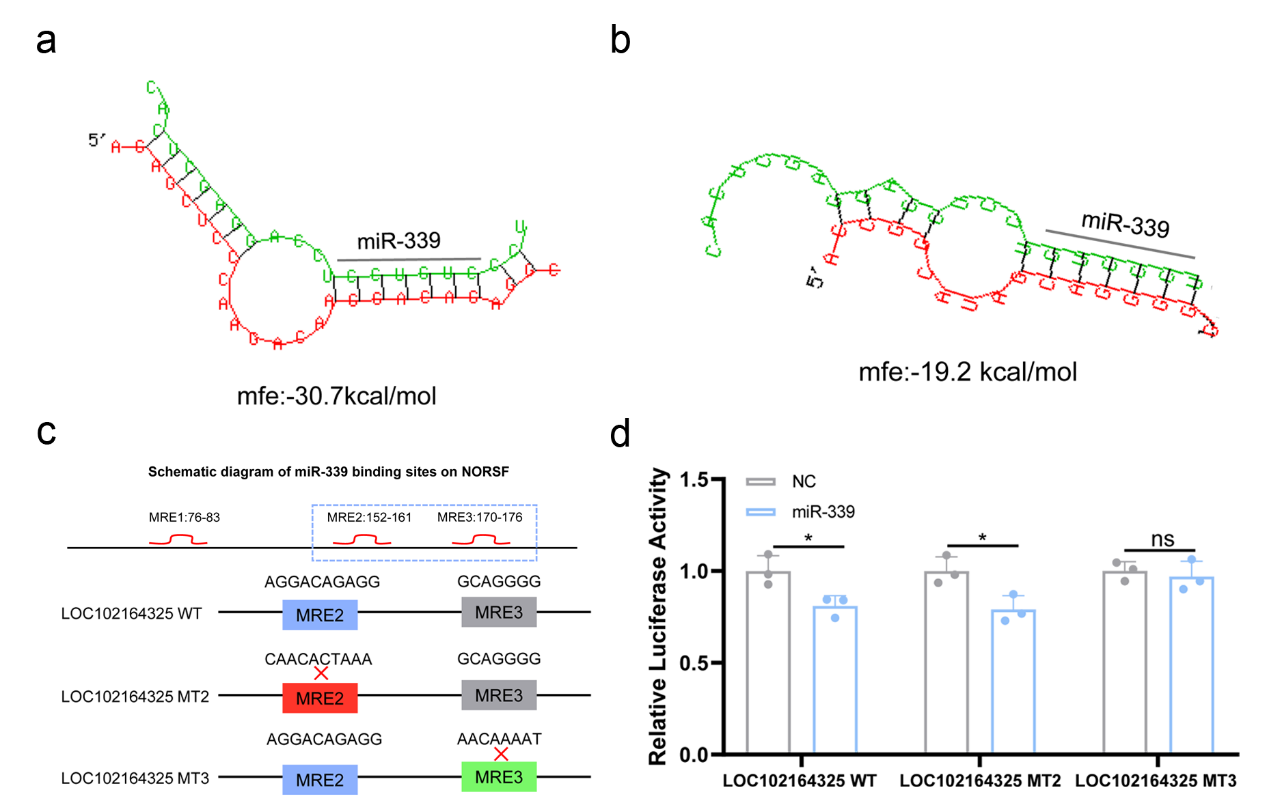


**Fig S3. Other two potential MREs of miR-339 in LOC102164325.**

**a-b** MRE2 (a) and MRE3 (b) motifs of miR-339 in LOC102164325 were predicted by RNAhybrid. mfe, minimum free energy. **c** Diagram of reporter vectors of LOC102164325 with MRE2 and MRE3 motifs. WT, wild-typed MRE2 and MRE3 of miR-339. MT2, mutant-typed MRE2 of miR-339. MT3, mutant-typed MRE3 of miR-339. **d** Luciferase assay. GC line KGN were co-transfected with reporter vectors and miR-339 mimics, and luciferase activity was detected. n=3. Values are means ± SEM. *, P<0.05; ns, no significant.


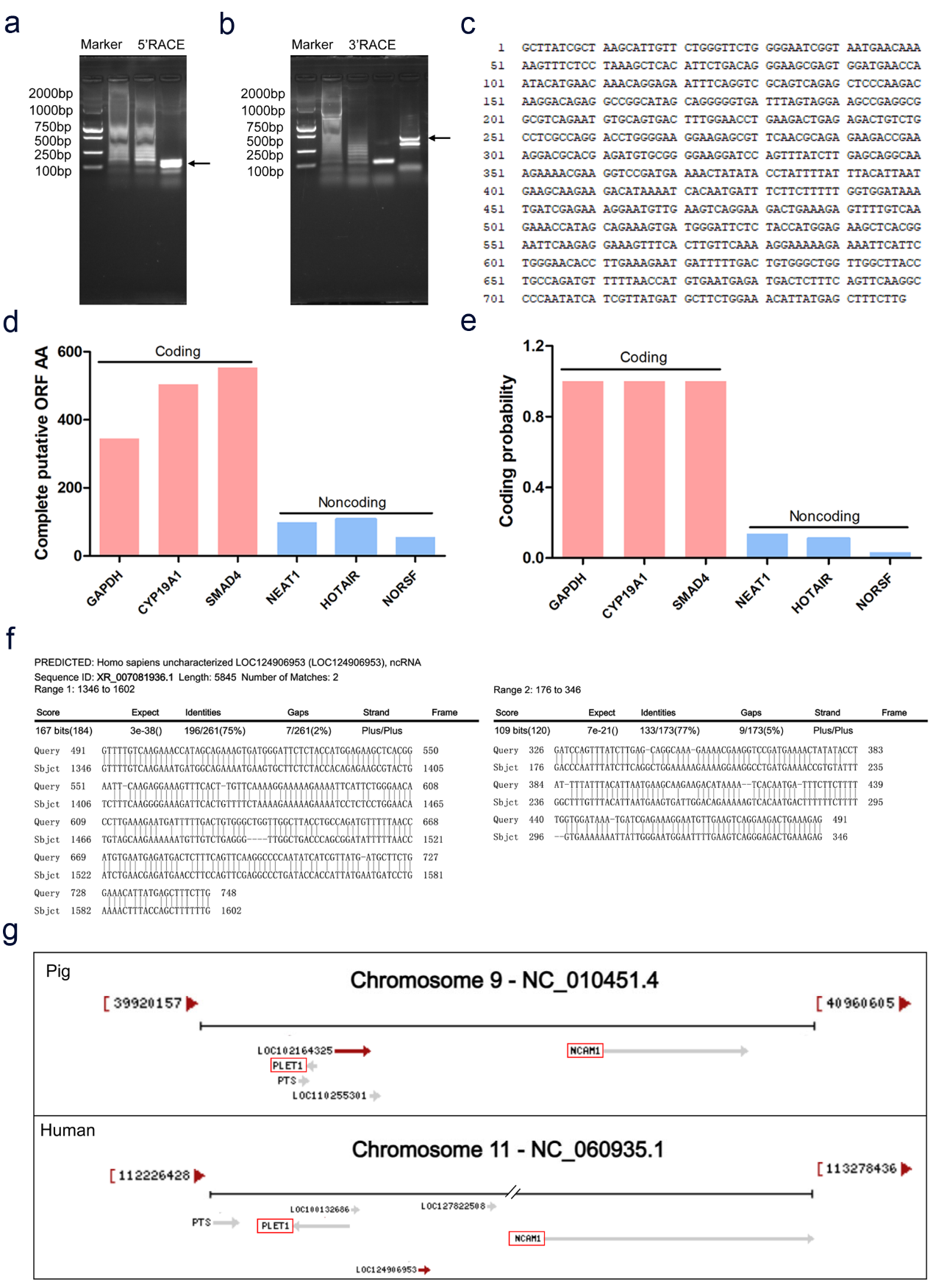


**Fig S4. Identification of lncRNA NORSF.**

**a-b** Gel images showing the products of 5′-RACE (a) and 3′-RACE (b). Arrows indicate the target fragments. **c** The sequence of lncRNA NORSF transcript. **d-e** Prediction of the coding potential of NORSF transcript by using two tools CPAT (d) and CPC (e). **f** Alignment of partial sequences of NORSF with human LOC124906953. **g** Genomic location of the porcine NORSF and human LOC124906953.


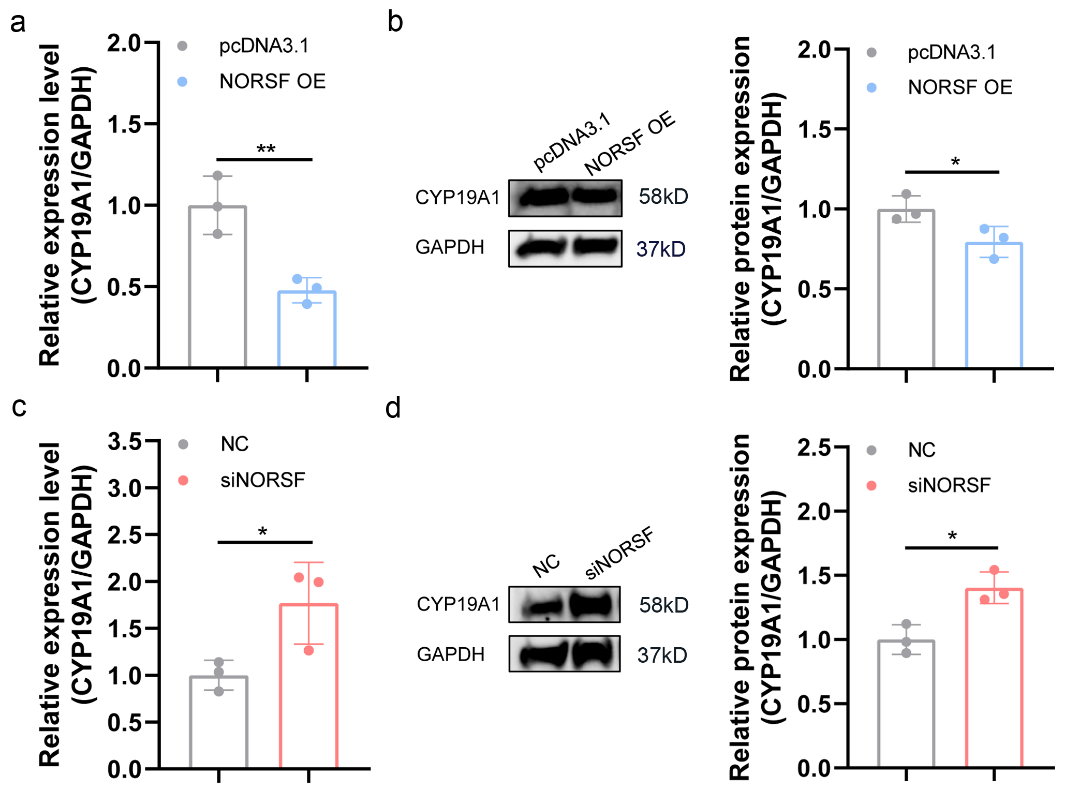


**Fig S5. NORSF negatively regulates CYP19A1 expression in GCs.**

**a-b** Overexpression of NORSF reduces CYP19A1 levels. GCs were treated with pcDNA3.1-NORSF, CYP19A1 mRNA (a)and protein (b) levels were detected by qPCR and western blotting, and normalized by GAPDH mRNA and protein levels, respectively. n=3. **c-d** Silencing of NORSF enhances CYP19A1 levels. GCs were treated with NORSF-siRNA, CYP19A1 mRNA (c)and protein (d) levels were detected by qPCR and western blotting, and normalized by GAPDH mRNA and protein levels, respectively. n=3. Values are means ± SEM. *, P<0.05; **, P<0.01.


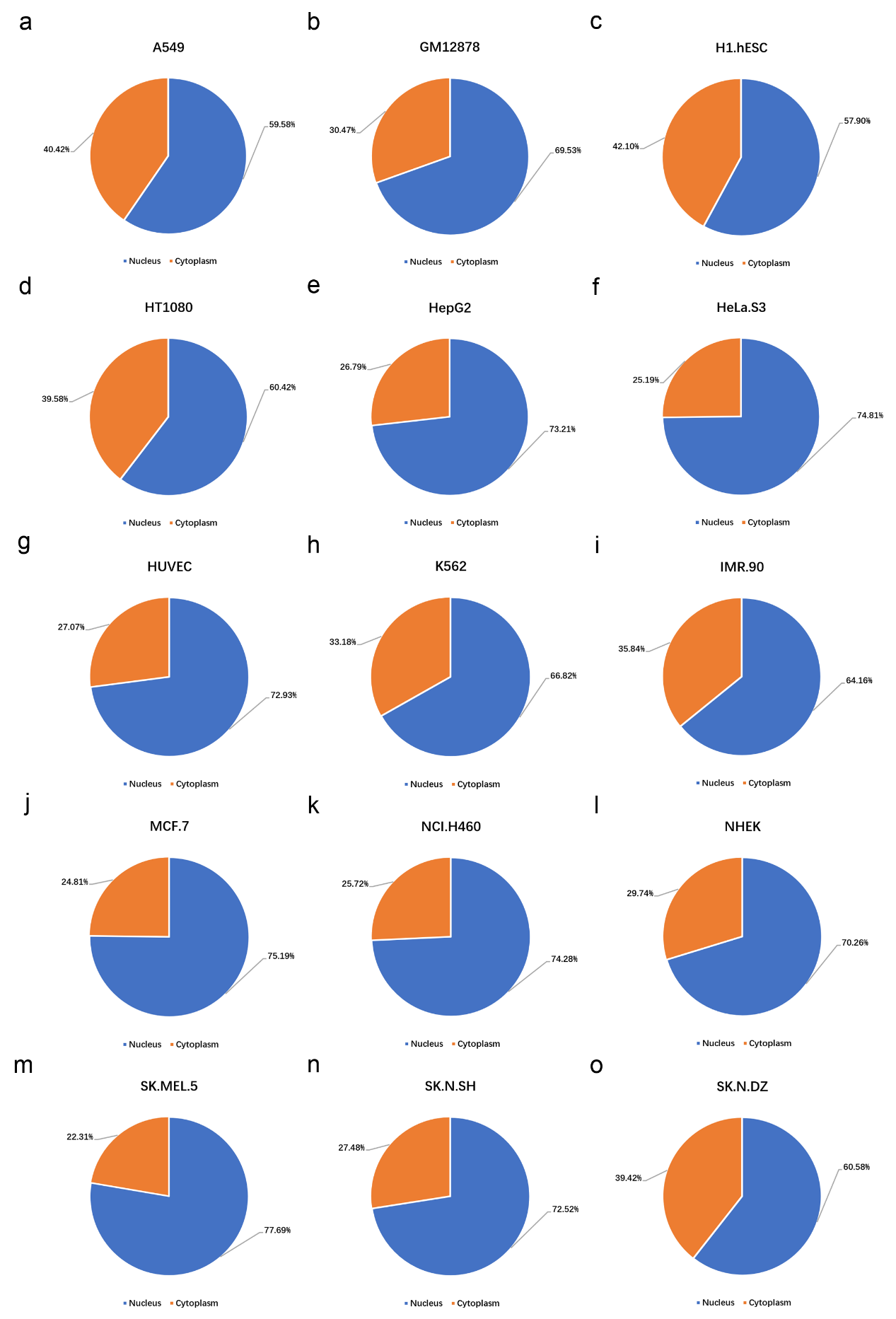


**Fig S6. Percentage of lncRNA nucleoplasm in multiple human cell types.**

Human cell types include A549 (a), GM12878 (b), H1.hESC (c), HT1080 (d), HepG2 (e), HeLa.S3 (f), HUVEC (g), K562 (h), IMR.90 (i), MCF.7 (j), NCI.H460 (k), NHEK (l), SK.MEL.5 (m), SK.N.SH (n), SK.N.DZ (o). Data were obtained from lncATLAS database (<https://lncatlas.crg.eu/>).


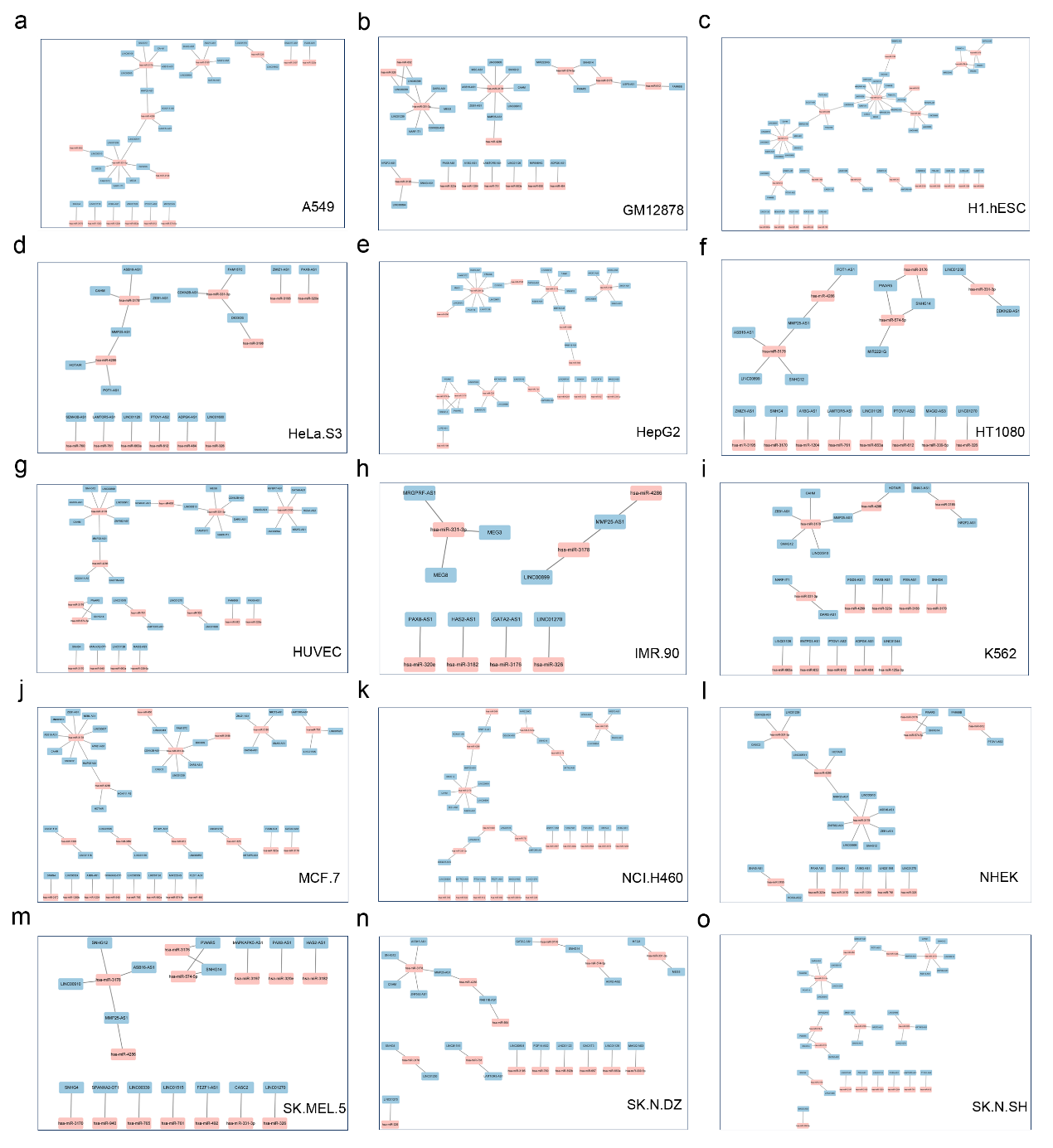


**Fig S7. Interaction networks of lncRNAs and miRNAs in the nucleus of multiple human cell types.**

Human cell types include A549 (a), GM12878 (b), H1.hESC (c), HeLa.S3 (d), HepG2 (e), HT1080 (f), HUVEC (g), IMR.90 (h), K562 (i), MCF.7 (j), NCI.H460 (k), NHEK (l), SK.MEL.5 (m), SK.N.DZ (n), SK.N.SH (o). Data for subcellular localization of miRNAs were obtained from RNALOCATE (http://www.rna-society.org/rna-locate/), and interaction networks were constructed by Cytoscape.

**
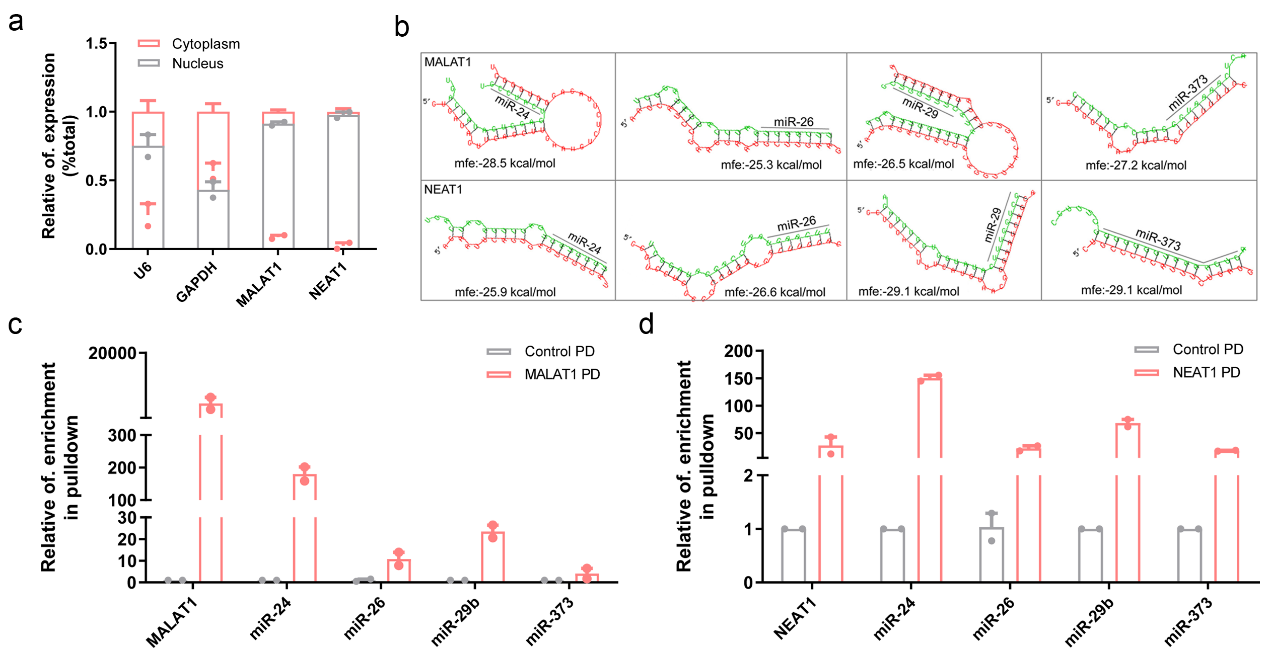
**

**Fig S8. Nuclear lncRNAs MALAT1 and NEAT1 interact with multiple miRNAs in the nucleus of human GCs.**

**a** Subcellular localization of MALAT1 and NEAT1 in human GCs. Levels of MALAT1 and NEAT1 in nuclear and cytoplasm fraction isolated from human GC line KGN were detected by qPCR. n=3. GAPDH and U6 were used as marker genes in the cytoplasm and nucleus, respectively. **b** MREs of four miRNAs in both MALAT1 and NEAT1 were predicted by RNAhybrid. **c-d** RNA pull-down assay. MALAT1-RNA complexes or NEAT1-RNA complexes were pulled down from nuclear RNAs in human ovarian GCs by using biotinylated probes for MALAT1 or NEAT1, levels of MALAT1 (c) or NEAT1 (d), and miR-24, miR-26, miR-29b and miR-373 were determined by qPCR, respectively. PD, pull-down. n=2. Values are means ± SEM.


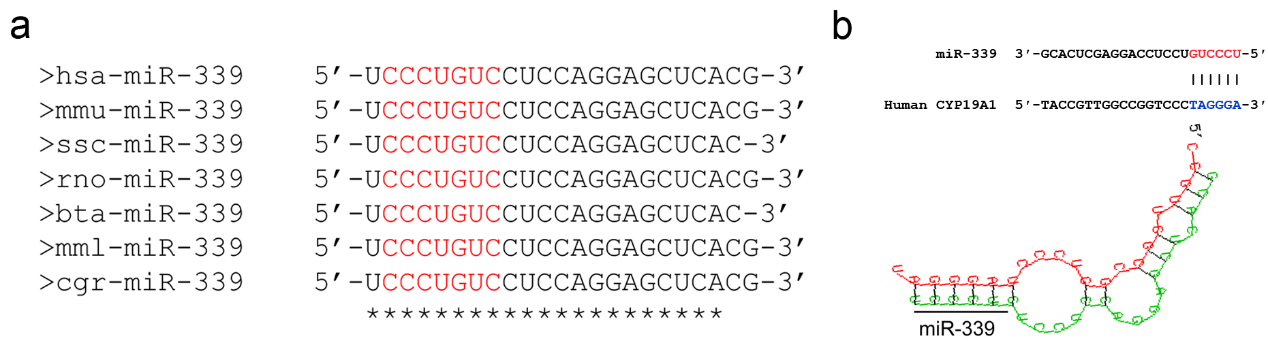


**Fig S9. miR-339 mature sequences are highly conserved among vertebrates.**

**a** miR-339 mature sequences. Asterisks indicate same sequence. has, *Homo sapiens*. mmu, *Mus musculus*. ssc, *Sus scrofa*. bta, *Bos taurus*. mml, *Macaca mulatta*. cgr, *Cricetulus griseus*. **b** MREs of miR-339 in the ovary-specific promoter of human CYP19A1 gene were predicted by RNAhybrid.


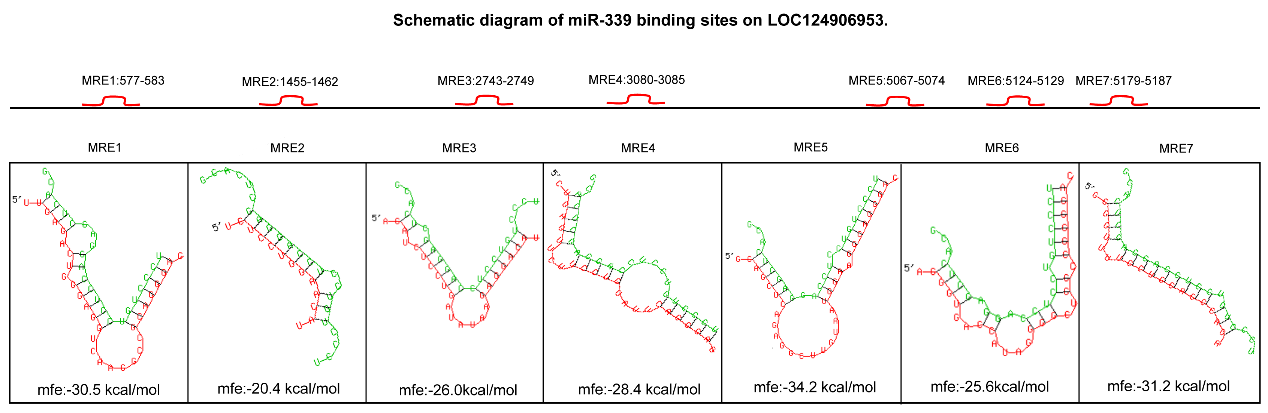


**Fig S10. The MRE motifs of miR-339 in human LOC124906953.**
