# Supplementary material for: Nuclear lncRNA NORSF reduces E2 release in granulosa cells by sponging the endogenous small activating RNA miR-339
Source: BMC Biol. 2023 Oct 20;21:221. doi: 10.1186/s12915-023-01731-x (PMC10588145; doi:10.1186/s12915-023-01731-x)
Supplement: Supplementary file 4 — Additional file 4: Original image 1. Electrophoresis original images. Original image 2. RACE original image. Original image 3. Western blotting original images. Original image 4. CHIP original images. [file 12915_2023_1731_MOESM4_ESM.doc]

**Original images 1**. Electrophoresis original images

Fig S1


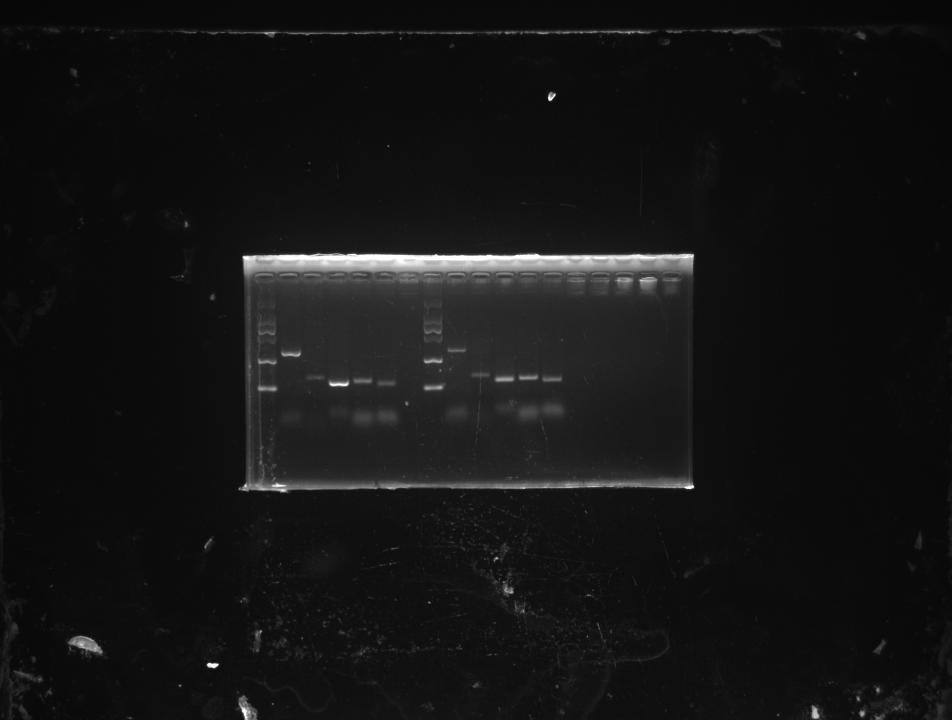
 lncRNAs


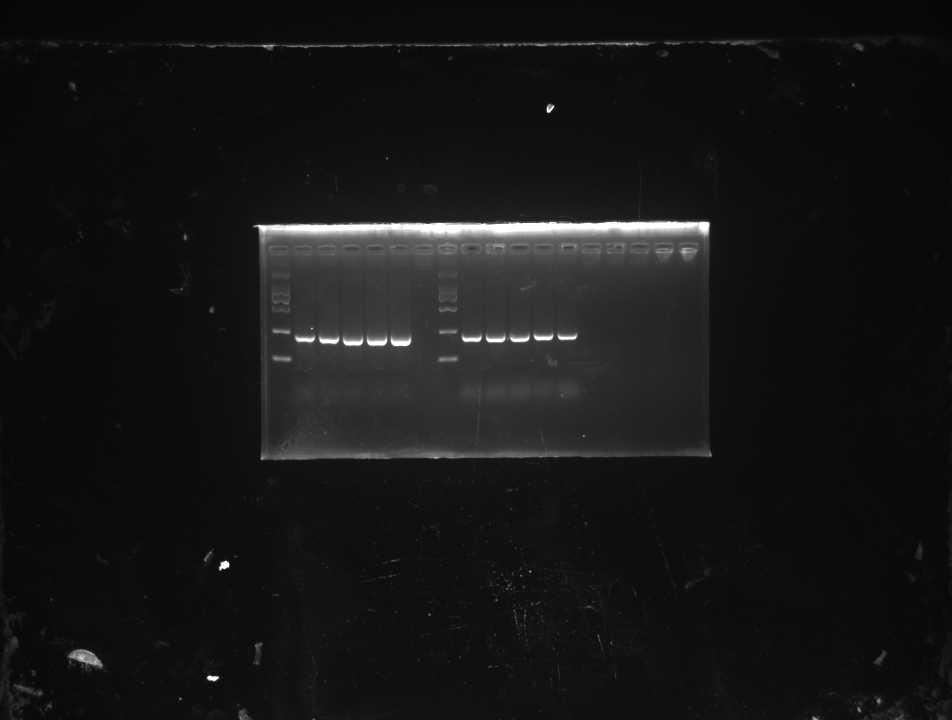
 GAPDH

**Original images 2**. RACE original image

Fig S4a-b


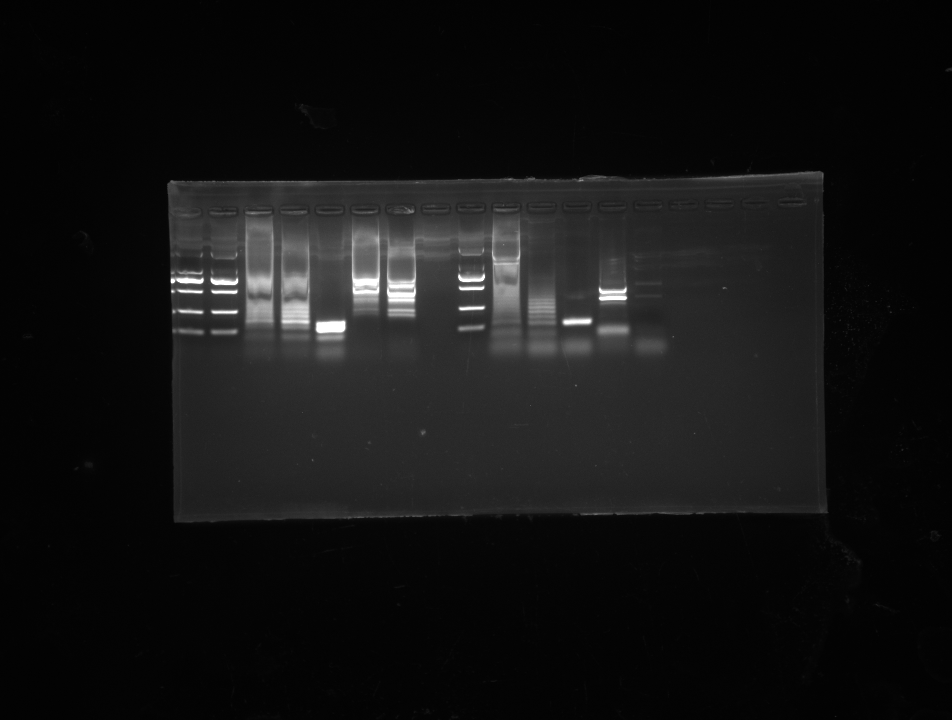


**Original images 3**. Western blotting original images

Fig S5b

pcDNA3.1 + - + - + -

NORSF OE - + - + - +


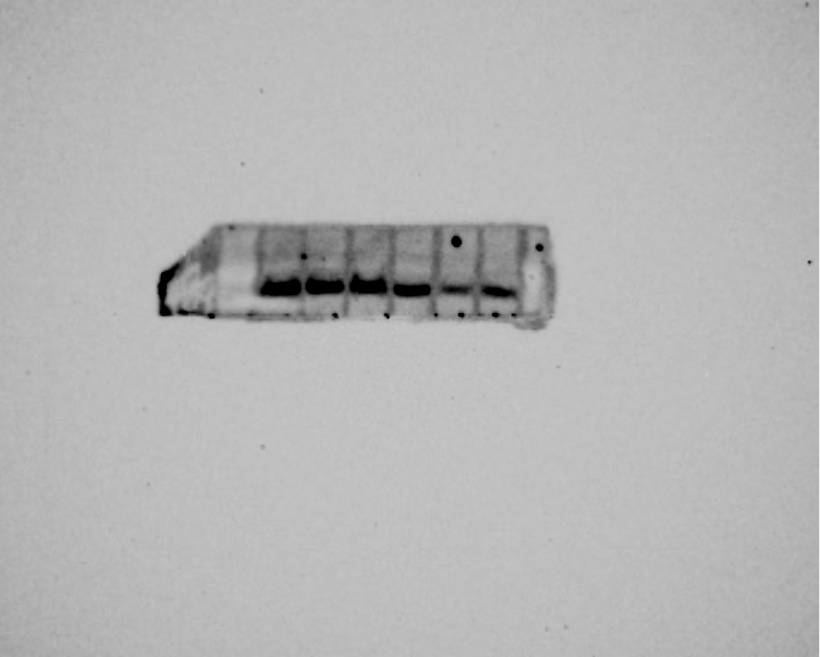
 CYP19A1


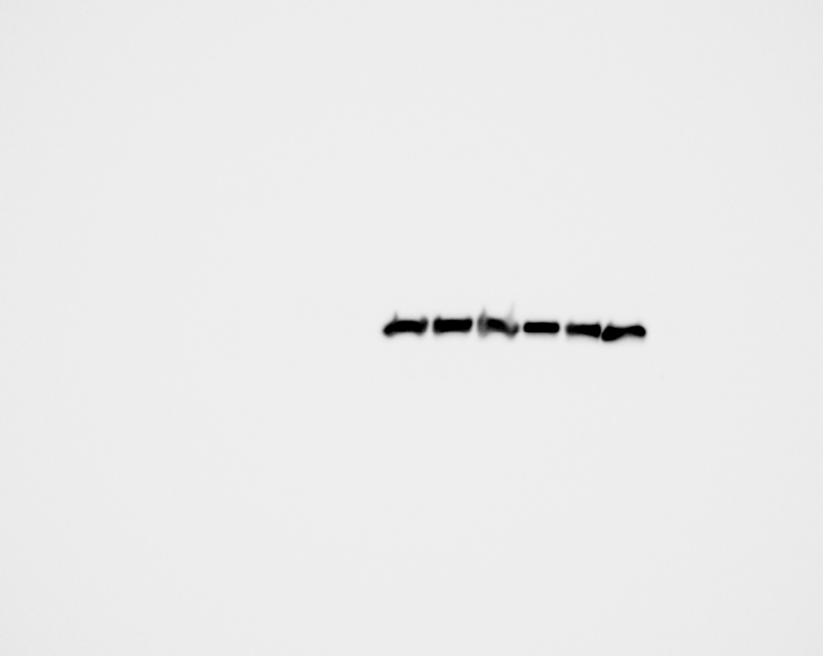
 GAPDH

Fig S5d

NC + - + - + -

siNORSF - + - + - +


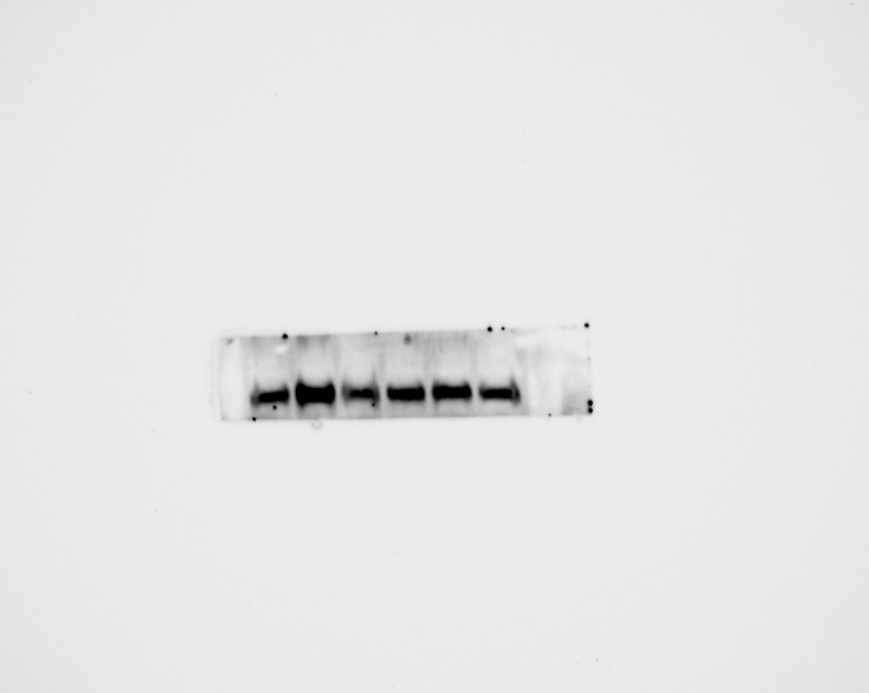
 CYP19A1


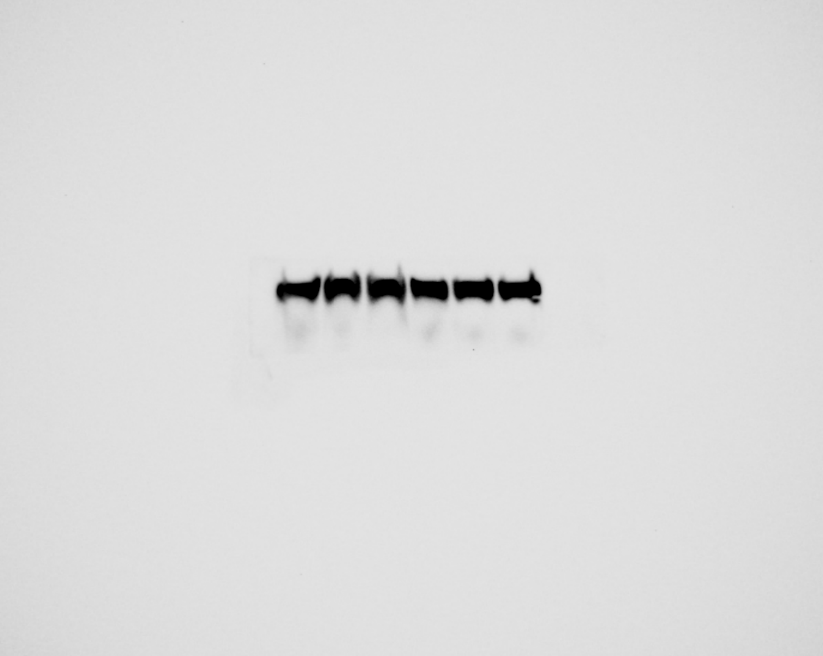
 GAPDH

Fig 3i

NC + + - + + - + + -

miR-339 - - + - - + - - +

pcDNA3.1 + + - + + - + + -

NORSF OE - + + - + + - + +


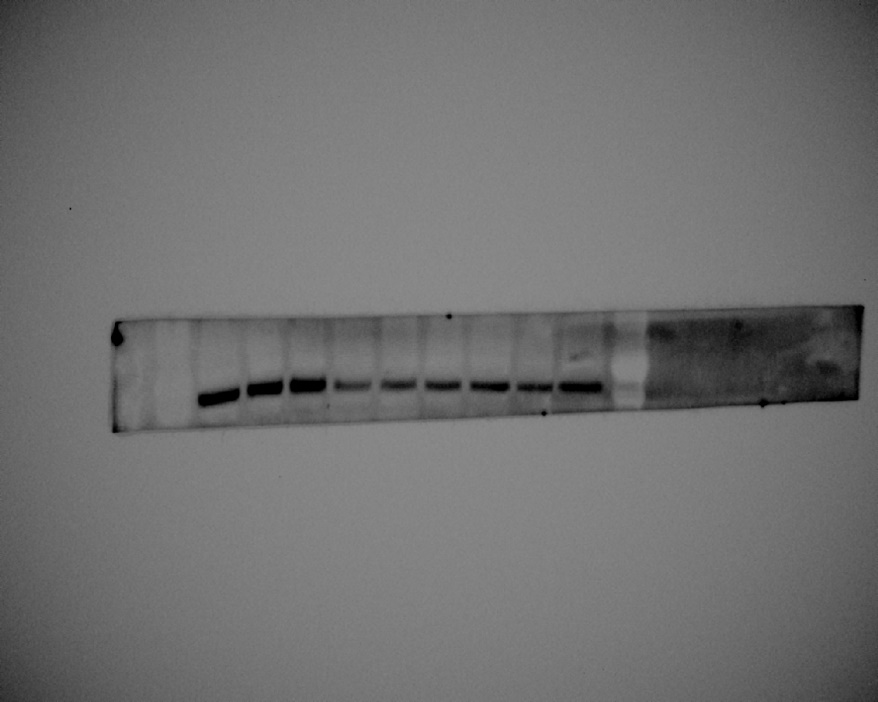
CYP19A1


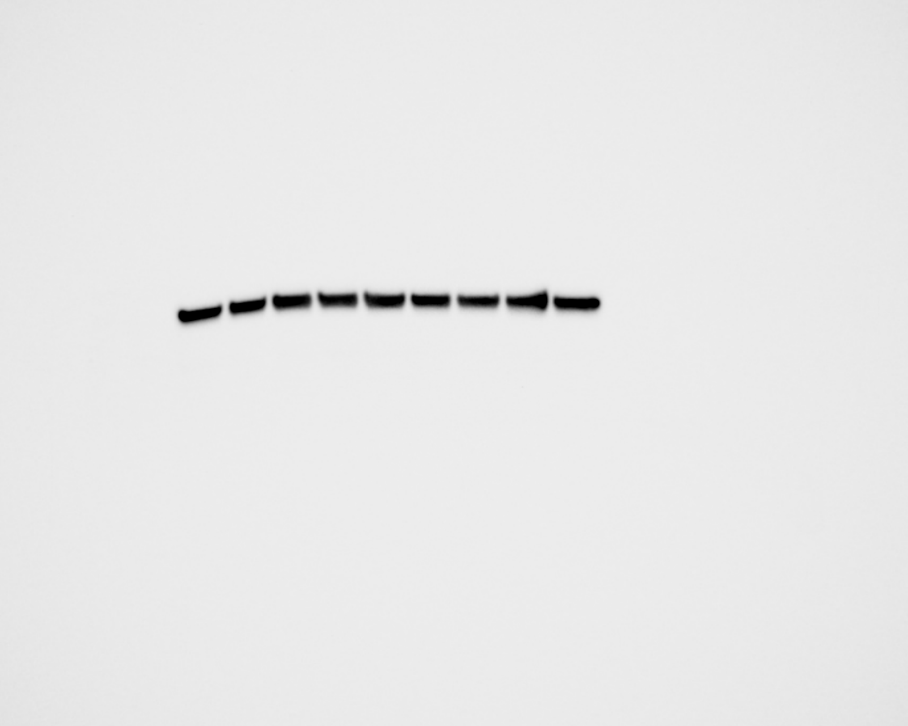
 GAPDH

Fig 4c

NC + - + - + - 24h

miR-339 - + - + - +


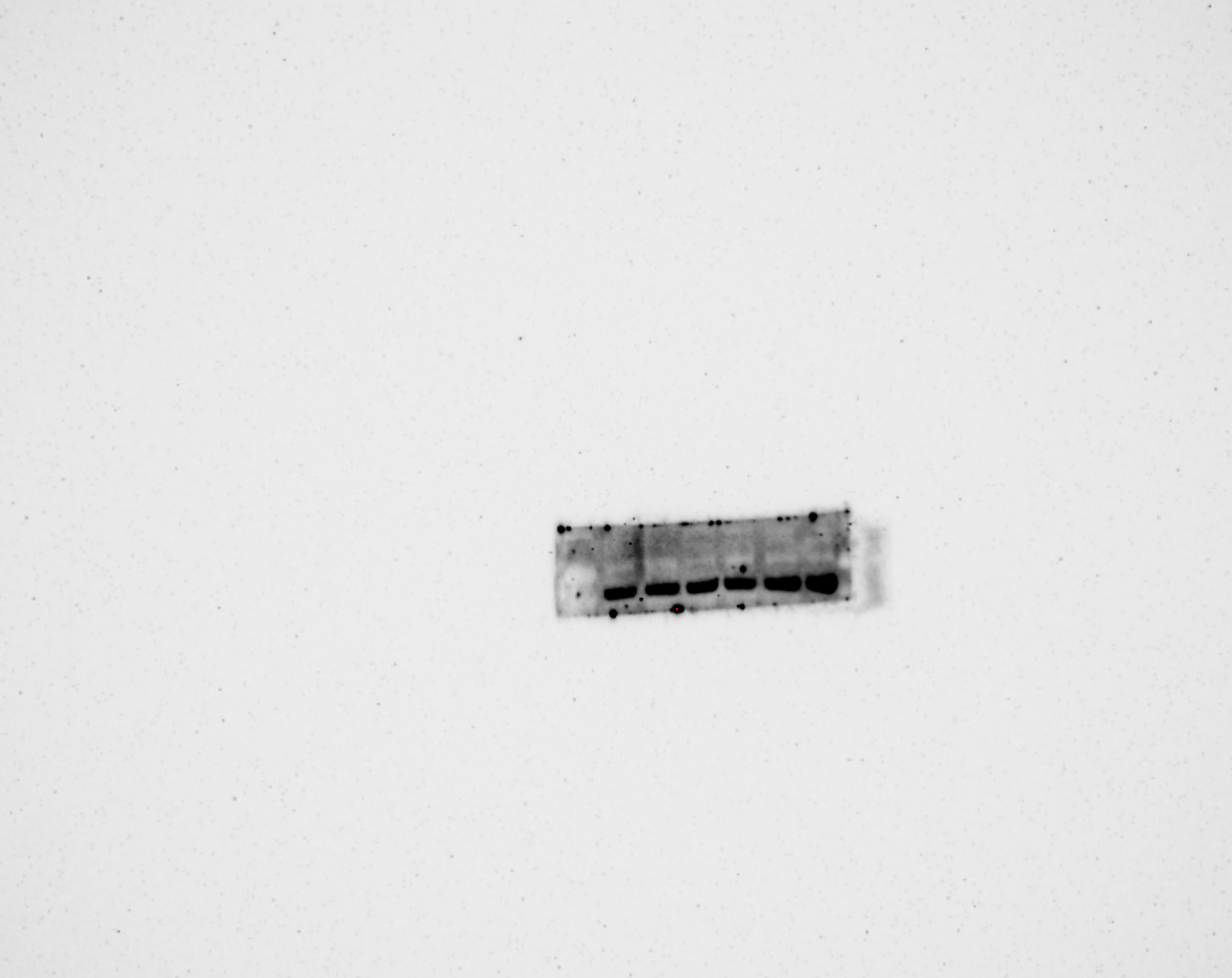
 CYP19A1


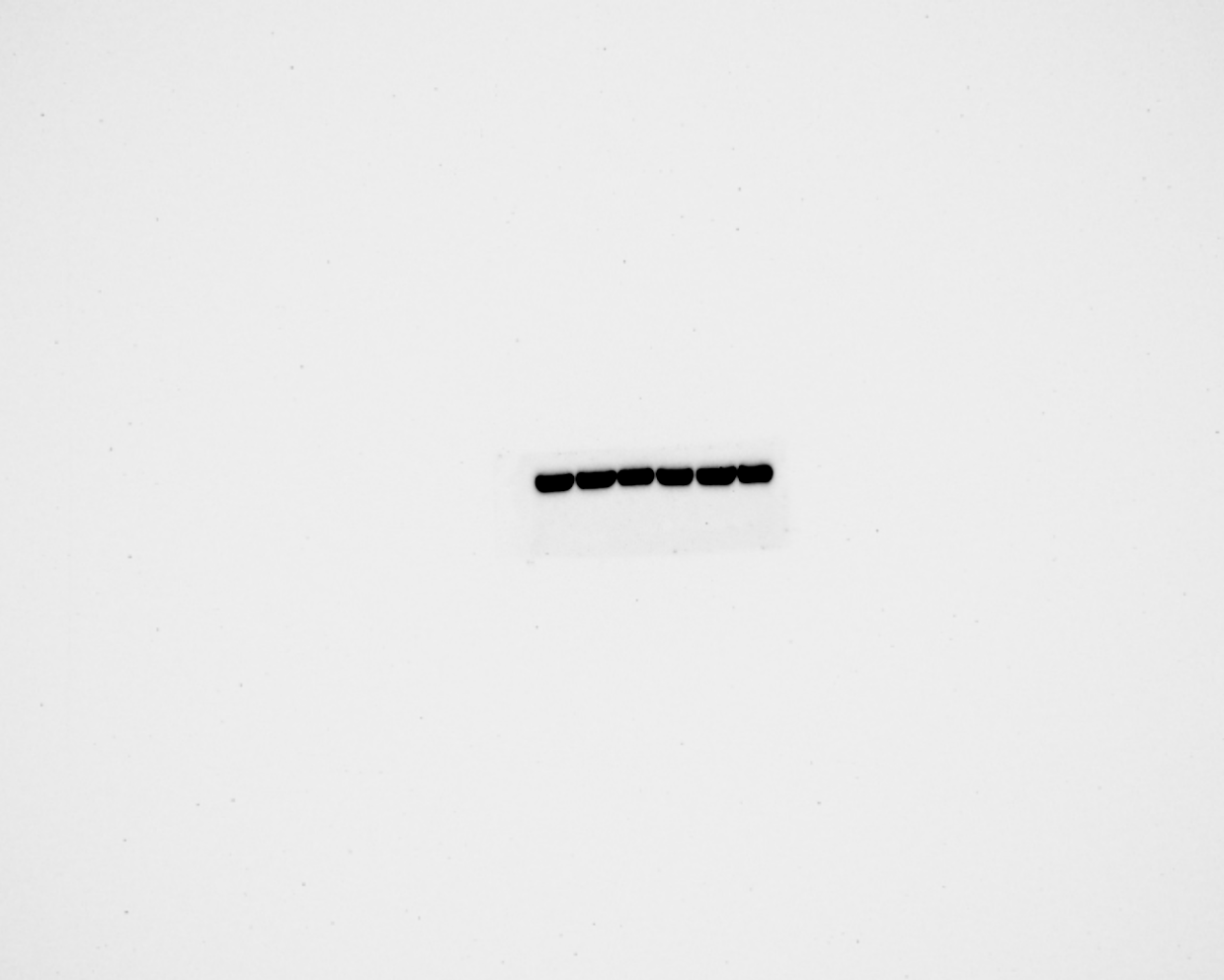
 GAPDH

Fig 4d

NC + - + - + - 48h

miR-339 - + - + - +


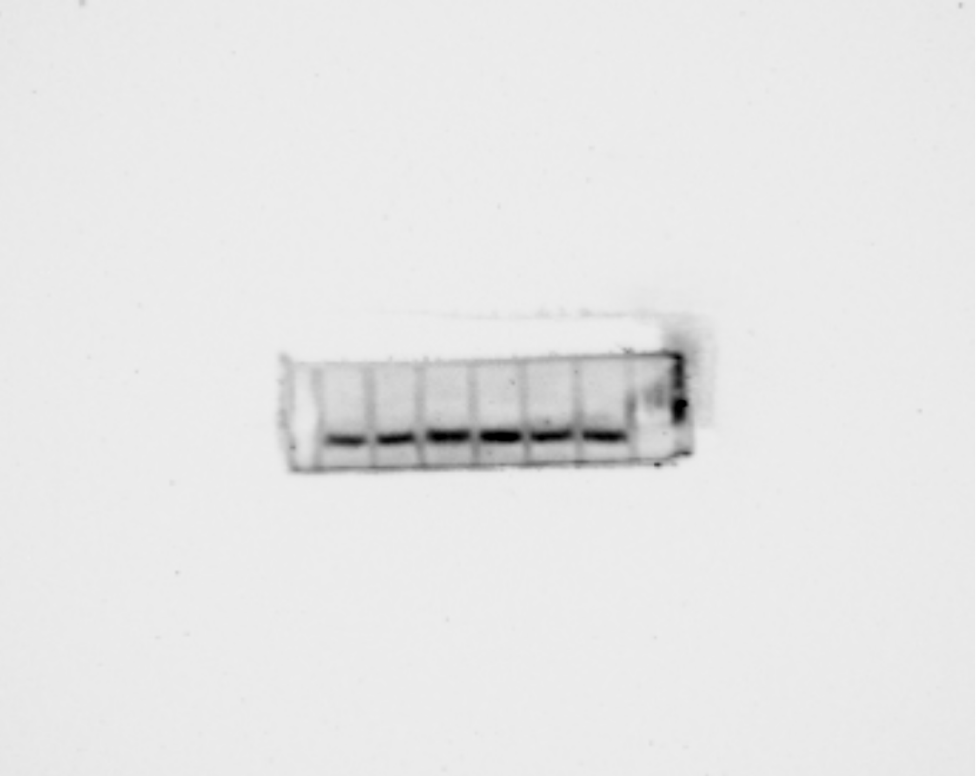
 CYP19A1


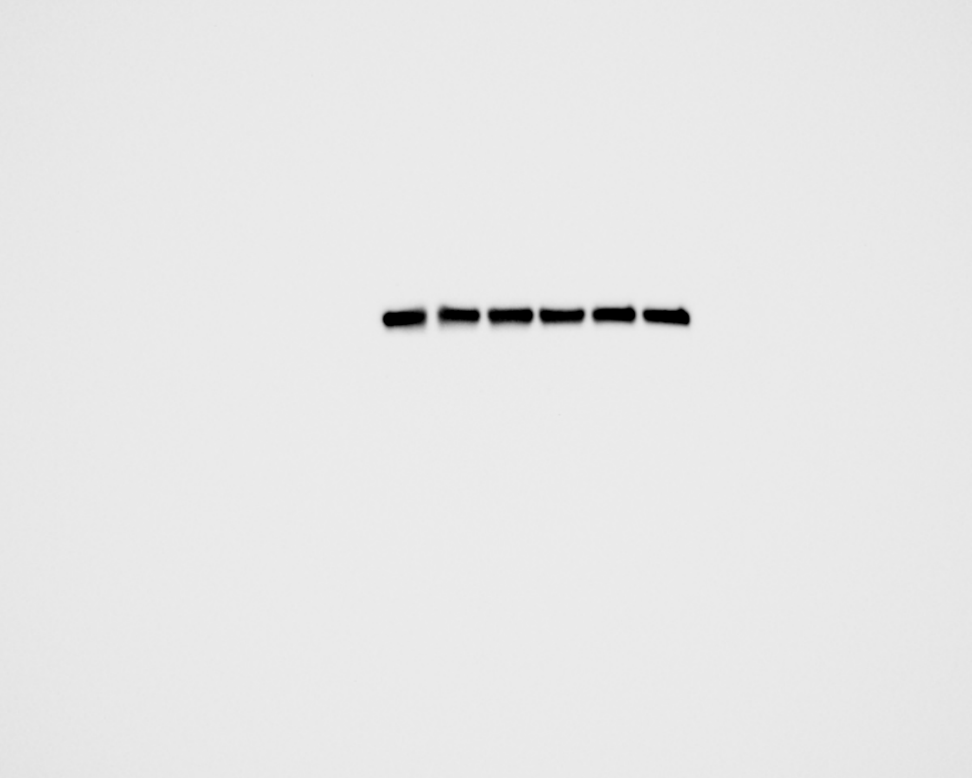
 GAPDH

**Original images 4**. CHIP original images

Fig 4f


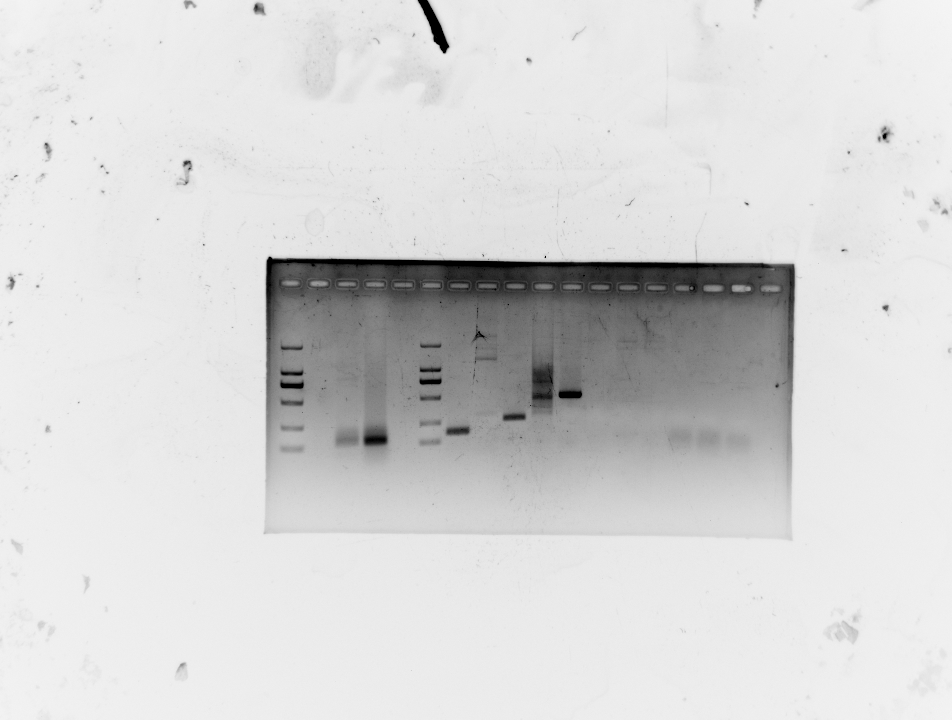


Fig 4g


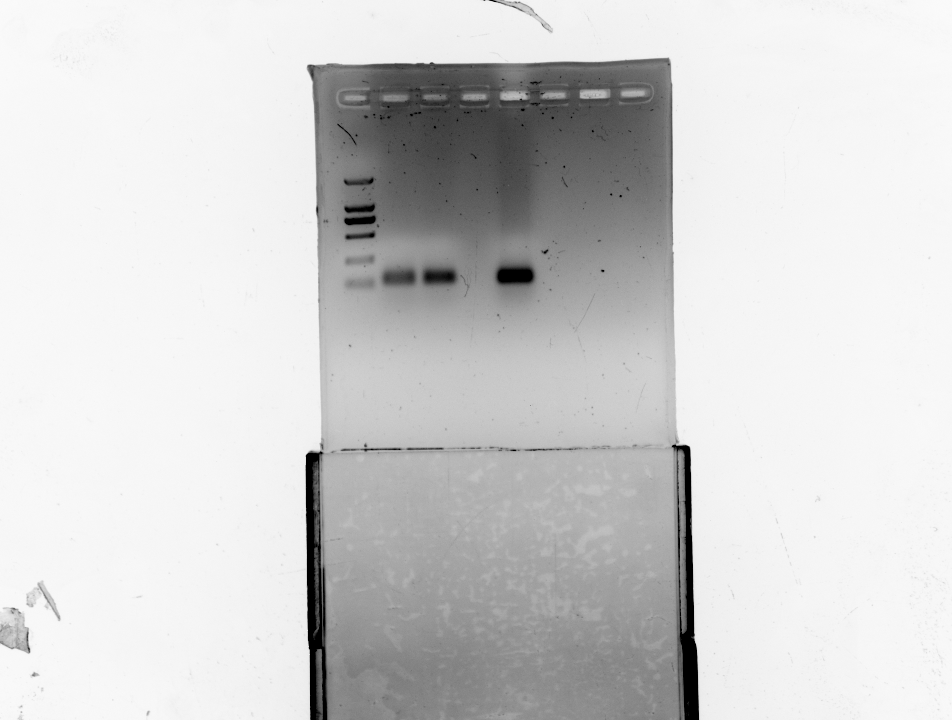
 H3K4me3


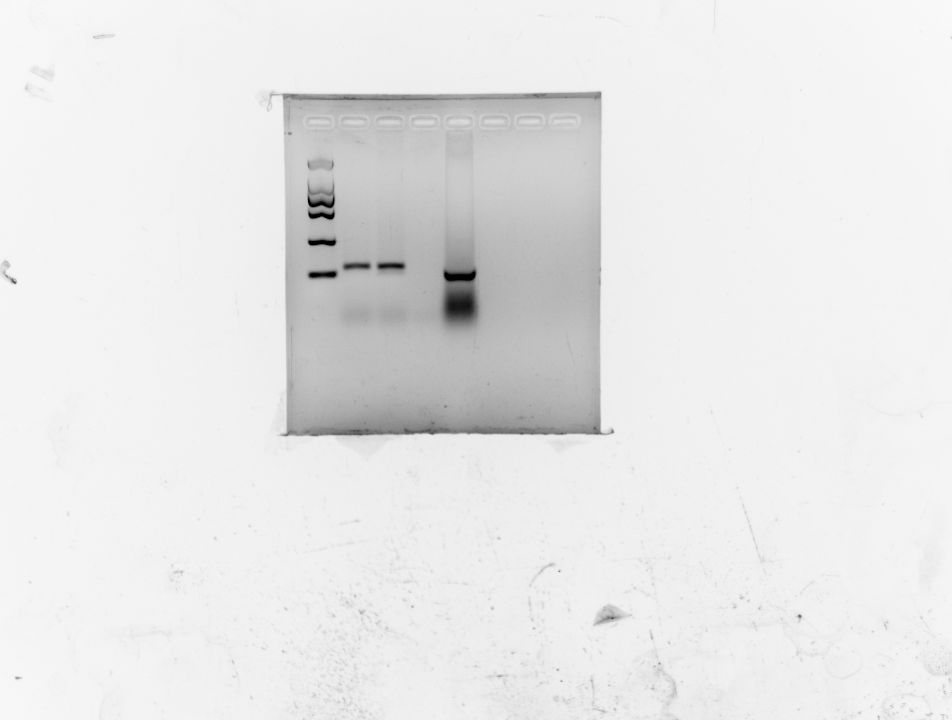
 H3K9ac


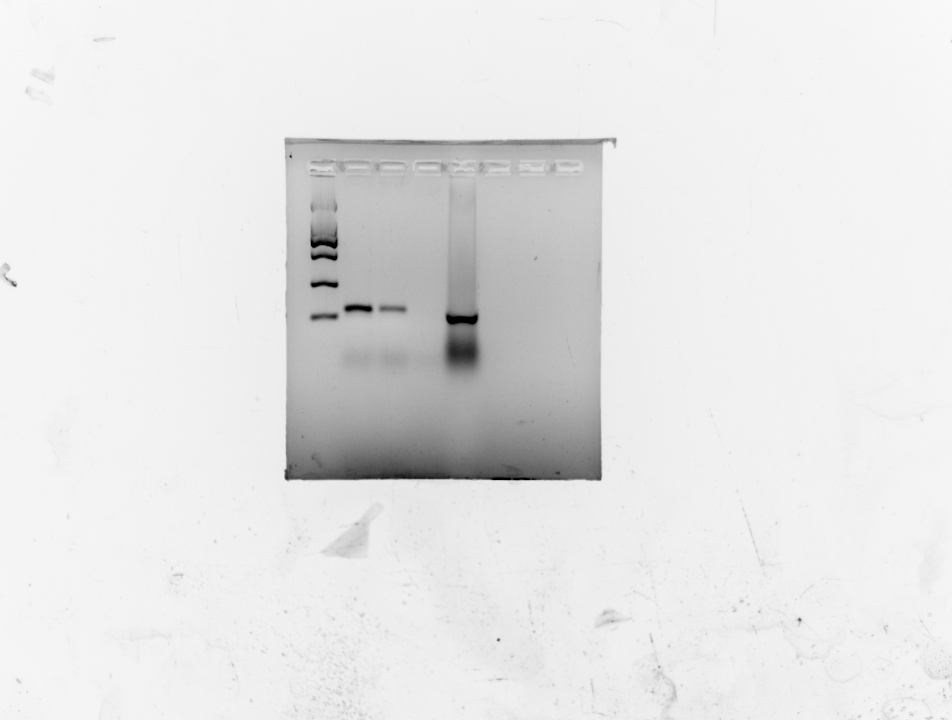
 Input
